# A topological solution to object segmentation and tracking

**DOI:** 10.1073/pnas.2204248119

**Published:** 2022-10-06

**Authors:** Thomas Tsao, Doris Y. Tsao

**Affiliations:** ^a^OpticArray Technologies, Rockville, MD 20850;; ^b^Department of Molecular & Cell Biology, Helen Wills Neuroscience Institute, Berkeley, CA 94704;; ^c^HHMI, Berkeley, CA 94704

**Keywords:** surface representation, segmentation, tracking, binding problem, symbolic representation

## Abstract

The world is composed of objects, the ground, and the sky. Visual perception of objects requires solving two fundamental challenges: 1) segmenting visual input into discrete units and 2) tracking identities of these units despite appearance changes due to object deformation, changing perspective, and dynamic occlusion. Current computer vision approaches to segmentation and tracking that approach human performance all require learning, raising the question, Can objects be segmented and tracked without learning? Here, we show that the mathematical structure of light rays reflected from environment surfaces yields a natural representation of persistent surfaces, and this surface representation provides a solution to both the segmentation and tracking problems. We describe how to generate this surface representation from continuous visual input and demonstrate that our approach can segment and invariantly track objects in cluttered synthetic video despite severe appearance changes, without requiring learning.

## Significance

We address a question at the foundation of natural and artificial vision: how can a visual system segment and track objects? In the real world, objects can undergo drastic changes in appearance due to deformation, perspective change, or dynamic occlusion. For example, an animal moving behind a fence will split into multiple pieces. How can a visual system apply a common label to all the pieces of the same object across space and time? Here, we prove that this can be solved using purely geometric mechanisms and furthermore demonstrate the approach on cluttered synthetic video. By enabling the automatic transformation of visual information from a sensory to symbolic format, the mechanism described here provides a springboard from sensation to intelligent symbolic reasoning.

Through a process of perceptual organization that is still not well understood, the primate visual system transforms visual input consisting of a stream of retinal images into a percept of stable, discrete objects. This process has traditionally been broken down into two separate problems: the “segmentation problem,” which addresses how visual pixels can be grouped into distinct objects within a single image (1), and the “tracking problem,” which addresses how objects can be identified across images despite changing appearance (2).

**Fig. 1.**
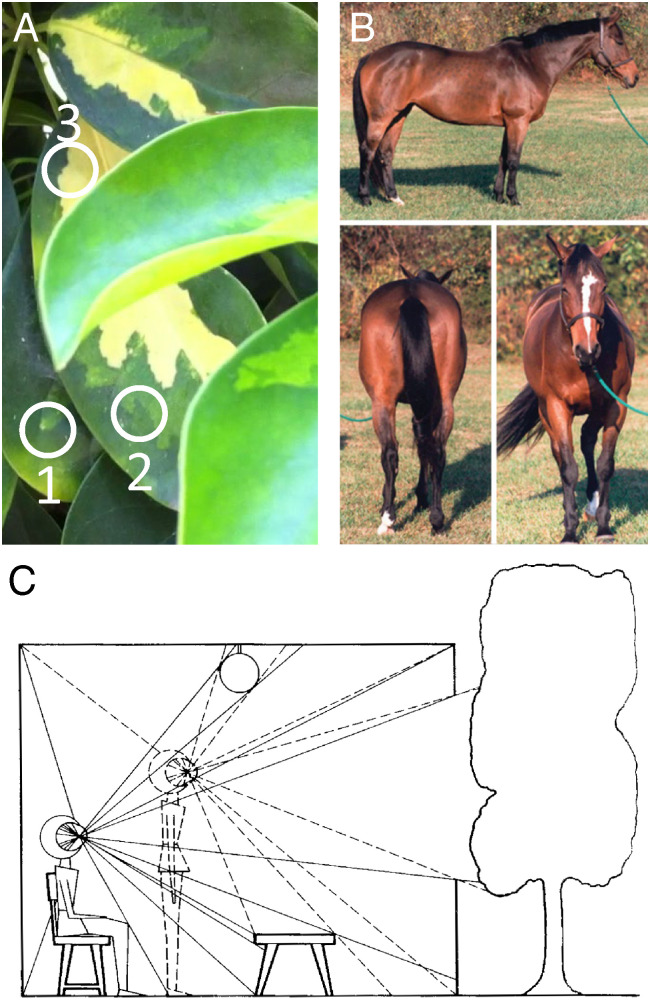
The challenge of object segmentation and tracking and Gibson’s proposed framework for solution. (*A*) The challenge of segmentation: Points 1 and 2 are nearby and have the same color but belong to different objects, while points 2 and 3 are distant and different in color, but belong to the same object. (*B*) The challenge of invariant tracking: The three views of the horse are very different in shape and pixel composition yet represent the same object. (*C*) Gibson’s ecological approach to visual perception. An array of light rays from objects in the environment is sensed at each point in the observation space (two are illustrated). Gibson asserted that transformations between these arrays contain all the information necessary to solve the segmentation and invariant tracking problems (reproduced from ref. 20).

Both problems are highly challenging. Segmentation is difficult because distant pixels of different color/texture can belong to the same object, while neighboring pixels of the same color/texture can belong to different objects (Fig. 1*A*). Tracking is difficult because the appearance of the same object can change drastically due to object deformation, changing perspective, or dynamic occlusion (Fig. 1*B*). The segmentation problem has classically been tackled through intensity-, color-, and texture-based region-growing approaches relying upon properties extracted from single images (3), and more recently through deep learning approaches. The tracking problem has been approached through probabilistic dynamical modeling (4) or “tracking by detection” (5–8), with recent methods incorporating deep learning (9–15). While earlier learning approaches to segmentation and tracking were supervised (10, 16), requiring large labeled training sets, more recently unsupervised approaches have emerged (15, 17). In this paper, we explore the computational origin of the ability to segment and invariantly track objects and show that this problem can in principle be solved without learning, supervised or unsupervised.

Complementing image-based approaches to segmentation and tracking, a geometry-based approach considers vision as an inverse graphics problem (18). In this framework, the visual system infers three-dimensional (3D) surfaces from images by inverting a 3D graphics model. However, because the third dimension is lost during perspective projection onto the retina, this inverse inference process is considered to be not fully constrained (19), implying that extensive learning from experience is necessary. In this paper, we show that the problem of inferring 3D surfaces from images is in fact fully constrained, if the input is in the form of a sequence of images of a scene in which either the observer or objects are moving. We demonstrate through both mathematical analysis and computational experiments that with only two natural assumptions, namely, 1) the world is composed of objects, that is, a discrete set of smooth textured surfaces with locally constant lighting, and 2) animals view the world from a moving observation point, it is possible to solve the problem of segmenting and invariantly tracking each discrete surface in the environment without requiring learning. Our computational experiments are limited to synthetic video, and we assume access to high-quality images, but as we argue below, our approach should be readily extendable to natural conditions.

Our paper is essentially a mathematical translation of the “ecological approach to visual perception” developed by the psychologist J. J. Gibson (20). Gibson pointed out that the key to understanding human vision is to insert between the 3D environment and the eye a new item, the field of ambient optic arrays. The ambient optic array at one point in space consists of the 2D distribution of light rays passing the point from illuminated surfaces in the environment (Fig. 1*C*). Gibson pointed out that the field of ambient optic arrays is governed by a set of laws which he dubbed “ecological optics,” and these laws can explain much of visual perception: “Instead of making the nervous system carry the whole burden of explaining perception, I wish to assign part of this burden to light itself. Ecological optics is my way of doing so” (20). In the decades since Gibson proposed his ecological optics approach to vision, this important concept has attracted growing attention in the computer vision community (21, 22).

We explain how Gibson’s theory can be formulated in precise mathematical terms and be implemented computationally. Mathematical analysis shows that object surface information is redundantly represented by the field of ambient optic arrays through two of its topological structures: the pseudogroup of stereo diffeomorphisms and the set of infinitesimal accretion borders. Formulated in terms of ecological optics, vision is a fully constrained, well-posed problem. Complete information for perception of objects as discrete, persistent units is contained in the visual environment itself within the field of ambient optic arrays.

The main paper has three parts. In the first part, we give a broad overview of our approach. In the second part, we present the mathematical theory of ecological optics (this part heavily references *SI Appendix* and may be skipped without loss of comprehension of the remainder of the paper). In the third part, we show how to exploit ecological optics computationally to solve the segmentation and invariant tracking problems. In addition, *SI Appendix* provides a self-contained and expanded exposition of the ideas.

## 1. Surface Representation: Overview

Unlike taste and touch, vision allows an animal to experience the environment without immediate contact. In vision, the link between the distal stimulus (objects in the environment) and proximal stimulus (light impinging on the retina) is the light reflected from environment surfaces, which at each point of observation forms what Gibson called the “optic array.” We will prove, in the next section, that information sufficient to both segment and track surfaces is faithfully represented in the field of optic arrays by transformations between visual images across a sequence of observation points. We will then demonstrate, in *Section 3*, how to compute these transformations and use them to perform object segmentation and tracking. While understanding the proofs requires a basic understanding of differential topology, the essential ideas, which we summarize in this section, are highly intuitive.

**Fig. 2.**
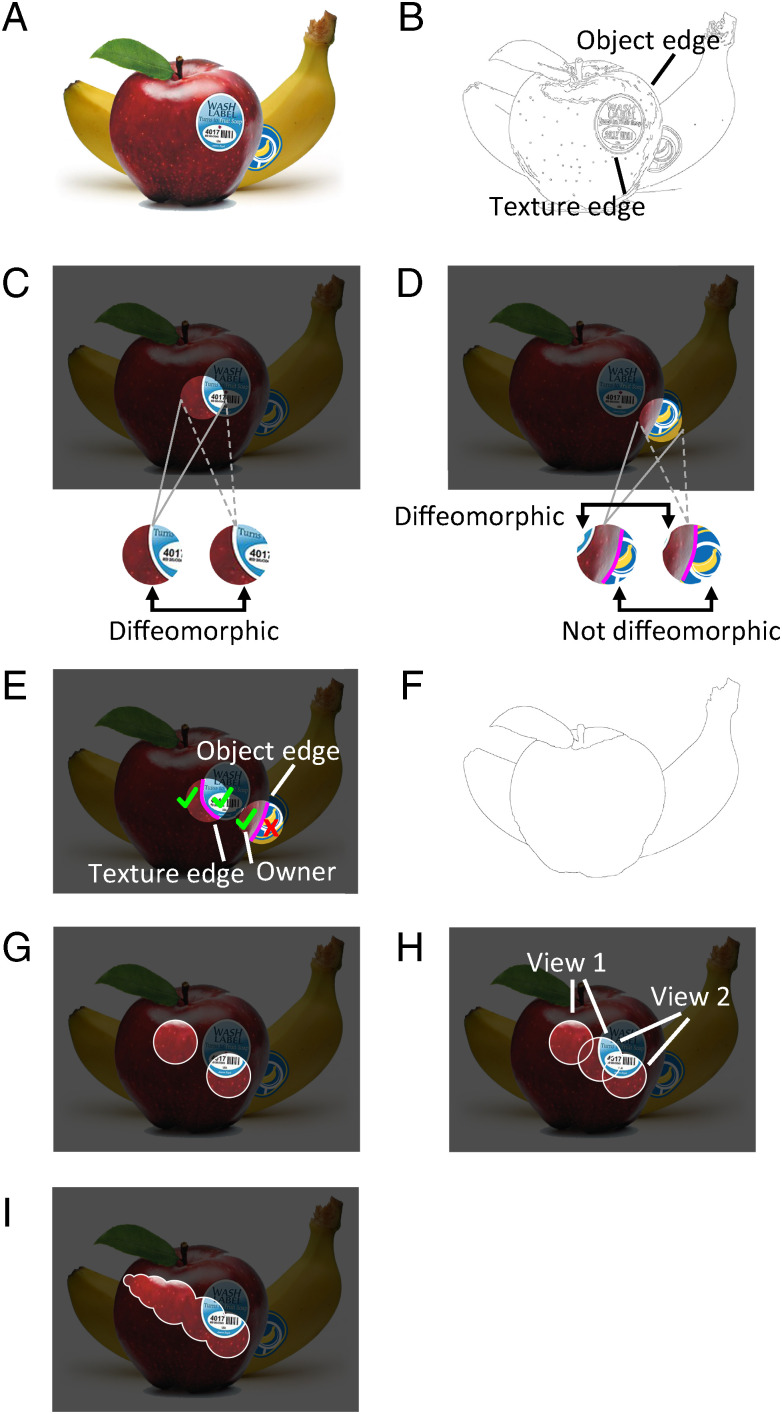
Topological solution to object segmentation and tracking: overview. (*A*) An example scene. (*B*) Edge map of the scene. (*C*) The projections of a region of space containing a contiguous surface patch to two observation points are diffeomorphic to each other. (*D*) The projections of a region of space containing an object edge to two observation points are diffeomorphic on one side (namely, the side that owns the edge) but non-diffeomorphic on the other side. (*E*) Two- versus one-sided diffeomorphism provides an effective criterion for distinguishing texture edges from true object edges. (*F*) Removal of texture edges produces a map of true object boundaries. (*G*) The invariance problem posed within the surface representation framework: How can one determine that the two distinct patches belong to the same surface? (*H*) Solution to the invariance problem: If one can identify a third patch overlapping both patches, then all three belong to the same surface. In this way, partial views (view 1, view 2) can be connected through overlaps. Thus the same diffeomorphism computation used to solve segmentation (*C* and *D*) can be used to solve tracking. (*I*) Through the equivalence relation of partial surface overlap, all possible views of an object can be identified.

Given a complex scene containing multiple objects (Fig. 2*A*), the goal of segmentation is to identify object boundaries. An efficient way to approach this is to start with a map of all the edges in the image (Fig. 2*B*), since object boundaries should be a subset of these edges. The key difficulty is that some edges are “texture edges” (e.g., the edge of the sticker in Fig. 2*A*), while others are true object edges (e.g., the edge of the apple in Fig. 2*A*). We prove that information in the transformation between nearby perspectives of a scene can be used to distinguish these two types of edges. Specifically, if a region of space contains a patch of surface, then two image patches taken from nearby observation points will be diffeomorphic to each other; that is, one can register them by stretching and warping like a rubber sheet (Fig. 2*C*). Furthermore, we show how to compute this diffeomorphism computationally through an iterative optimization scheme in which a set of local Gabor receptive fields dynamically undergo affine transformation to cancel the transformation between the two image patches (see Fig. 5). However, if an image patch contains an object edge, then on one side of the edge the image patches will be diffeomorphic, but on the other side they will not be, because there will be a piece of the background visible from one perspective but not the other, leading to a one-sided breakdown in diffeomorphism (Fig. 2*D*). In visual psychophysics, this phenomenon has been referred to as “da Vinci stereopsis” (23). This provides an effective way to distinguish texture edges from true object borders (24): For each edge element, determine the existence of diffeomorphism on each side of the edge. Object borders are accompanied by diffeomorphism on only one side. Moreover, we can identify this as the side that owns the edge (Fig. 2*E*). By repeating this process across the entire image, we can convert an edge map into a truly informative map of object borders (Fig. 2*F*).

Once segmentation has been framed in this surface representation framework, the solution to the invariant tracking problem, which has been considered one of the hardest problems in vision (25, 26), becomes almost trivial. How can we know whether two discrete patches (e.g., the two patches shown in Fig. 2*G*, or the front and back views of a horse) belong to the same invariant surface? We can determine this by checking whether the two patches are connected through a series of overlapping surface patches (Fig. 2 *H* and *I*). Thus, in the surface representation framework, an invariant object constitutes an equivalence class of surface patches, where the equivalence relation is defined by surface overlap. Importantly, the same diffeomorphism machinery for solving segmentation also allows us to compute these surface overlaps, and thus to connect (i.e., track) different views of the same surface over time. Even if a surface undergoes a drastic transformation in appearance (e.g., the front and back views of a horse), as long as successive views are related by local diffeomorphisms, then the tracking process can readily link the views.

## 2. Surface Representation: Mathematical Theory

In this section, we express the laws of ecological optics mathematically. We show that the data for solving the segmentation and invariance problems, and more generally, for obtaining a representation of visual surfaces, is sufficiently and redundantly available in the animal’s proximal visual environment. We formulate the problems of segmentation and invariance as follows: Is it possible to determine whether two image patches (seen from a sequence of observation points) belong to the same physical surface? For the case of a single view, this corresponds to the segmentation problem; for the case of a continuous series of views over time, this corresponds to the tracking problem. Our solution to these problems, already summarized in the previous section, is categorically different from those proposed previously. It relies on a key property, surface contiguity, that is topological and not image based, and computed from pairs of images taken from different perspectives rather than from single images.

We introduce two topological spaces: one for describing the 3D objects in the environment (the distal stimulus) and one for describing the light rays reflected from these objects and converging at each observation point in the environment (the proximal stimulus). We study the mapping between these two spaces and prove that information about the topological organization of objects in the former space is faithfully represented in the latter space. In other words, we prove that visual perception of invariant objects is possible.

**Fig. 3.**
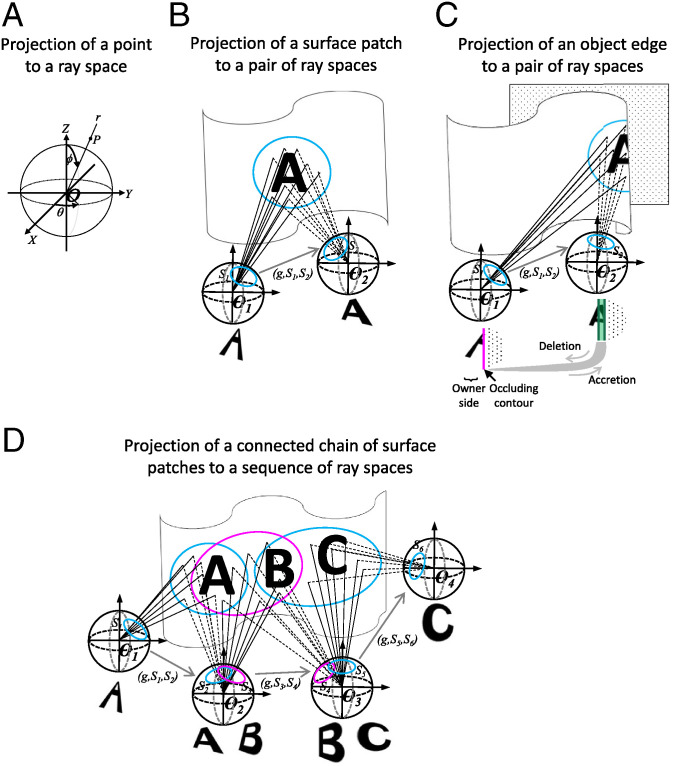
Coding local and global surface contiguity through stereo diffeomorphisms. (*A*) Point *P* projects to ray space *S*(*O*) with value ray *r =* (*θ, φ*) in a polar coordinate system. (*B*) Surface contiguity in the distal Euclidean space is faithfully encoded in the proximal visual space. If a neighborhood of a point is the perspective projection of a contiguous local surface patch in the environment (e.g., the surface patch containing the letter “A”), then a stereo diffeomorphism can be found from this neighborhood to a neighboring ray space. The pairs of intersecting rays correspond to stereo pairs in the transition space *S*(*O*_1_) × *S*(*O*_2_). (*C*) Surface discreteness in the distal Euclidean space is faithfully encoded in the proximal visual space. No diffeomorphism can be found between a neighborhood in a ray space containing points of an occluding contour to a neighboring ray space. In the figure, an occluding contour segment in the ray space at *O*_1_ is marked by the short vertical magenta line; it is the ray space image of an object fold under perspective projection, and constitutes a border of infinitesimal accretion because, following any change in observation point away from the owner side, for example, to *O*_2_, there is accretion; that is, the two side images of the border (the two dark green vertical lines) are now regular and have no intersection point. The owner of the occluding contour is specified by the side opposite the accretion (see *SI Appendix*, Fig. SM14 for more details). Occluding contours provide a compact and complete representation of environment surfaces because all points in a ray space that are not in an occluding contour possess a neighborhood representing a local surface patch. (*D*) Surface persistence in the distal Euclidean space is faithfully encoded in the proximal visual space. Image patches A and C in ray spaces based at *O*_1_ and *O*_4_ represent parts of the same contiguous environment surface because they are connected by a chain of overlapping stereo neighborhoods; that is, they are *CC*(Ω) equivalent. In detail, image patch A at *O*_1_ and image patch A at *O*_2_ are *MS*(Ω) equivalent, as are image patches B at *O*_2_ and B at *O*_3,_ and image patches C at *O*_3_ and C at *O*_4_. Image patches A at *O*_2_ and B at *O*_2_ are overlapping, as are image patches B at *O*_3_ and C at *O*_3_. Thus the *MS*(Ω)-equivalence class containing image patch A at *O*_1_ is connected to the *MS*(Ω)-equivalence class containing image patch B at *O*_3,_ and the latter is further connected to the *MS*(Ω)-equivalence class containing image patch C at *O*_4_. Thus image patch A at *O*_1_ is *CC*(Ω) equivalent to image patch C at *O*_4_. This scheme allows extremely different views of the same global surface (e.g., the three views of the horse in Fig. 1*B*) to be perceived as belonging to the same global persistent surface.

Specifically, we prove that the property of local surface contiguity is specified by the existence/nonexistence of a particular type of mapping between pairs of images taken from different perspectives, namely, “stereo diffeomorphism”; this provides the key to topological image segmentation (Fig. 3 *A*–*C*). We further prove that two surface representations are of the same object if they each contain a part related by stereo diffeomorphism; this global topological property provides the key to invariance (Fig. 3*D*).

### 2.1. The Geometry of Light Rays in an Environment Containing Objects and Multiple Observation Points.

Let *O* be a potential observation point in the medium. We call the set *S*(*O*) of all rays starting at the point *O* the *ray space* based at *O* (Fig. 3*A*). The space containing all rays with their base point located in a domain of the observation space Ω (i.e., set of all potential observation points) is the space VS(Ω) = *S* × Ω. We call VS(Ω) the *visual space* on the *observation domain* Ω. The space spanned by two ray spaces *S*(*O*) and *S*(*O*′) represents all possible pairings of rays taken from *S*(*O*) with rays taken from *S*(*O*′). We call *S*(*O*) × *S*(*O*′) the transition space based at (*O, O*′).

We have two types of topological spaces: 1) the 3D Euclidean space of ordinary points for describing the spatial structure of the objects and their surfaces and 2) the ray spaces, transition spaces, and visual space for describing the spatial structure of light rays converging on every possible observation point. There are mapping relations between “points” of the two different types of topological spaces.

We use the term *environment* to refer to all the surfaces, the ordinary surfaces of 3D objects and the ground, and the sky which is considered a surface with each point at an infinite distance. A further mathematical assumption is that environment surfaces are piecewise smooth. The mapping from a point in the 3D Euclidean space to a ray space is given by point projection: let *P* be a point in the 3D Euclidean space, *O* be a point in a domain of observation, *P* ≠ *O*. We call the ray *r* ∈ S(*O*) the *image* of *P* if *P* is a point on *r* (Fig. 3*A*). A point of the visual environment is *visible from a point* of observation if the line segment connecting these two points does not intersect any other points on an ordinary surface.

A *perspective projection* from a surface to a ray space is a map generated by applying point projection to every visible point on the surface. The analytical structure of perspective projection from a general 2D manifold to another 2D manifold is the subject matter of differential topology. In particular, according to a theorem by Whitney (27), upon perspective projection of environment surfaces to ray spaces, the points in each ray space are divided into two sets: the set of *regular* values, where the perspective projection is one-to-one continuous and differentiable, and the set of *critical* values occurring at the boundaries of regular domains, where this relation breaks down. This insight provides a means to compute surface contiguity and separation from information available in the proximal ray spaces.

In a rigid environment, the perspective changes with change of the point of observation (achieved by having two eyes or by physically displacing one eye). We call a pair of perspectives a *perspective transition*. We call the image of 3D Euclidean space in the 4D transition space the *stereo space*. Each of its elements is called a *stereo pair*. The stereo space constitutes the subset of ray pairs in the transition space that intersect in a point in 3D Euclidean space (Fig. 3*B*). A *stereo diffeomorphism* is a diffeomorphism between domains (*stereo neighborhoods*) of two ray spaces, such that each ray and its image form a stereo pair.

### 2.2. Coding Local Surface Contiguity.

Let *S* be a patch of surface in the environment visible from two observation points *O*_1_ and *O*_2_ (Fig. 3*B*). We prove that the images of *S* under perspective projection in *S*(*O*_1_) and *S*(*O*_2_) are related by stereo diffeomorphism *g* (*SI Appendix*, Existence of Stereo Diffeomorphism Theorem); conversely, given a stereo diffeomorphism *g* from a domain *S*_1_ in one ray space to *S*_2_ in another ray space, the stereo pairs in the transition space satisfying the constraint *g* specify a 2D manifold in the 3D stereo space (*SI Appendix*, Surfaciness Theorem).

We call *h =* (*g*, *S*_1_, *S*_2_) a *mapping triple*. Both *S*_1_ and *S*_2_ are diffeomorphic to the 2D surface patch specified by *h,* and, therefore, each qualifies as a topological representation of this patch. The mapping triple specifies not only the existence of a contiguous surface patch but also its metric properties of distance and curvature (*SI Appendix*, Shape from Perspective Mapping Theorem).

### 2.3. Coding Surface Spatial Separation.

In the previous section, we showed how local surface contiguity is encoded in pairs of ray spaces by mapping triples. Points in domains of mapping triples are regular values of perspective projection (*SI Appendix*, Local Stability of the Regular Value Set Theorem). A perspective projection can also have critical values, and these turn out to be the key to encoding surface spatial separation.

Whitney (27) proved that there are only two types of singularities of a smooth mapping from a 2D manifold to a 2D manifold: *folds* and *cusps* (*SI Appendix*, Fig. SM10). We call images of fold singularities in a ray space under perspective projection *occluding contours* (Fig. 3*C*, vertical magenta segment).

Occluding contours carry rich information about surface spatial separation and continuation. First, we prove that the two sides of an occluding contour represent spatially separated local surfaces; second, each of the local surfaces continues in a particular manner, with the owner side folding back, and the non–owner side extending behind the surface of the owner side (*SI Appendix*, Separated Surface Continuation Theorem). The brain appears to use this information effectively: Gestalt psychologists observed that the presence of occluding contours can remove the boundary of a figure and make it “incomplete” and thus trigger a process of “amodal completion” behind the occluding contour (28).

Occluding contours are defined as singularities of the mapping from the 3D Euclidean space to the visual space. But the visual system only has access to data in the visual space. What information in the visual space is available to detect occluding contours? The key insight is that an occluding contour is a border of infinitesimal accretion: There always exists a small domain of observation, such that the occluding contour is the border of accretion of a perspective transition within this domain (Fig. 3*C*, vertical green region and *SI Appendix*, Border of Accretion Criteria for Occluding Contours Theorem). Furthermore, the owner side can be computed as the side opposite that which undergoes perceivable accretion, while it itself remains topologically invariant, that is, is only subject to a diffeomorphic transformation. Note that, for a smooth surface like a sphere, there can be accretion on both sides. However, we can prove that, for shift of observation point of magnitude ε (where ε is small enough that ε^2^ ≪ ε), the width of the accretion on the owner side goes as ε^2^, while the width of the accretion on the background goes as ε (*SI Appendix*, Sided Division of Accretion Track Theorem). Because the amount of accretion on the owner side is so much smaller, it is easy to differentiate the two sides computationally.

### 2.4. Coding Global Surfaces in a Single Perspective: Ad Hoc Surface Representation.

Under perspective projection, a tuple *T* = (*C, B*) of a regular component (i.e., maximal connected set) *C* and its surrounding occluding contours *B* gives a representation of the global surface at a point of observation in the following sense: Component *C* represents a visible part of the global surface, and *B* represents the rest of the global surface. We call such a tuple an *ad hoc representation* of the global surface.

### 2.5. Coding Global Invariant Surfaces across Perspectives.

Once the machinery for generating an ad hoc surface representation in a single perspective through occluding contours is in place, extracting a globally invariant surface representation (i.e., a representation in which the same surface is identified across perspectives) is essentially trivial: The local contiguous surface components in each ad hoc representation can be simply stitched together through partial overlaps.

How can we identify representations of the same surface across perspectives? First, we define an equivalence relation among domains of regular values in the visual space: Domains *S*_1_ and *S*_2_ are equivalent if there is a perspective mapping triple (*g*, *S*_1_, *S*_2_) for domains *S*_1_ and *S*_2_ in the ray spaces of some pair of observation points. This equivalence relation divides the whole set of domains of regular values in the visual space defined on an observation domain Ω into different equivalence classes, each called an *MS(Ω)-equivalence class*, where *MS*(Ω), the *mapping structure* on *Ω,* is the total set of perspective mapping triples (*g*, *S*_1_, *S*_2_). The mapping structure forms a pseudogroup (29) on the visual space and provides the conceptual foundation for understanding visual invariance. Each of these equivalence classes represents a local surface patch of the environment invariant to perspective.

From these *MS*(Ω)-equivalence classes, we construct a perspective-invariant representation of a global surface as follows: If a pair of domains from two different *MS*(Ω)-equivalence classes have nonvoid intersection, we call the two *MS*(Ω)-equivalence classes *connected*. We call two *MS*(Ω)-equivalence classes *chain connected* if there is a chain of consecutively connected domains linking these two classes. Chain connectedness defines an equivalence relation. Each chain-connected *MS*(Ω)-equivalence class, denoted a *CC*(Ω)-equivalence class, represents the perspective-invariant global surface of a 3D object (*SI Appendix*, Fixed Owner Theorem).

Finally, we are ready to answer the question, How can ecological optics represent an invariant global surface across different perspectives? In each perspective, an ad hoc representation of the global surface is available if it is partially visible. Let *T*_1_ = (*C*_1_*, B*_1_) and *T*_2_ = (*C*_2_*, B*_2_) be two ad hoc representations in a perspective transition from observation point *O*_1_ to observation point *O*_2_. These two representations are perceived as encoding the same global surface if *C*_1_ and *C*_2_ are *CC* equivalent (Fig. 3*D*).

To summarize, we set out to understand whether it is possible to determine that two patches *S*_1_, *S*_2_ of ambient optic arrays in ray spaces at different observation points *O*_1_*, O*_2_ are perspective images of the same physical surface or not. We first sketched a mathematical framework: Light from object surfaces is mapped to ray spaces at each observation point in the environment through perspective projection, defining a mapping from the distal Euclidean space to the proximal visual space VS(Ω). We then searched for a stereo diffeomorphism between *S*_1_ at *O*_1_ and an image patch in a nearby observation point, and likewise for *S*_2_ at *O*_2_. The existence of these stereo diffeomorphisms means that *S*_1_ and *S*_2_ each represent some local surface patch (not necessarily belonging to the same global surface). Next, at both *O*_1_ and *O*_2_, we extend these local surface patches to ad hoc representations of global surfaces by identifying the occluding contours bounding *S*_1_ at *O*_1_, and bounding *S*_2_ at *O*_2_. Finally, we determine whether these ad hoc representations are of the same global surface by testing for chain connectedness between *S*_1_ and *S*_2_.

Thus the laws governing the optical projection of the visual environment to the visual space give rise to a topological representation of persistent environment surfaces in terms of 1) the ad hoc representation in each perspective defined by the set of regular and critical values and 2) the invariants across perspectives defined by equivalence relations given by the pseudogroup of stereo diffeomorphisms and chain connectivity. This persistent surface representation sets the stage upon which object perception functions.

We note that, in computer vision, Koenderink and van Doorn (30) were the first to try to explore the singularity structure of images for the purpose of understanding invariant perception of objects, but their goals were very different from ours. They observed that 1) self-induced movements of an observer generate motion-parallax fields, 2) the singularities of these fields correspond to folds and cusps [following Whitney (27)], which are stable for most vantage points and provide information about invariant object shape, and 3) at unstable points, these singularities can change in a specific number of possible ways to reveal new shapes (e.g., a hill transforming into a hyperbolic intrusion). Their main focus was extracting information about solid shape, while our main focus is segmentation and object tracking independent of shape; in neuroscience terms, this can be considered a distinction between “what” and “where” stream functions. Critically, in our theory, the invariance of surfaces is based upon the equivalence relation of partial overlap, not on “stability of singularities.”

## 3. Surface Representation: Algorithmic Implementation and Computational Experiment

So far, we have presented a theory of ecological optics. In the same way that geometric optics describes how points on an object are carried by light to points in the image plane, ecological optics describes how topologically important structures of object surfaces in 3D Euclidean space (i.e., properties such as contiguity, spatial separation, partial overlap, etc.) are carried by light to topological structures of rays in the visual space: regular components, perspective mappings, occluding contours, accretion/deletions around occluding contours, *MS*(Ω)-equivalence classes, and *CC*(Ω)-equivalence classes. The theory of ecological optics presented in the previous section describes the physical reality of the animal’s visual environment and does not depend in any way on the presence of a visual system. In this section, we demonstrate how a visual system that moves through the environment can computationally exploit the topological structures of rays in the visual space to perceive the topology of the visual environment, that is, to perceive discrete, invariant units.

### 3.1. Algorithmic Method for Segmentation and Invariant Object Tracking.

Given a sequence of video frames of a scene in which either the observer or objects are moving, our goal is to segment each frame according to surface contiguity and assign the same label to surface components corresponding to the same object across frames.

**Fig. 4.**
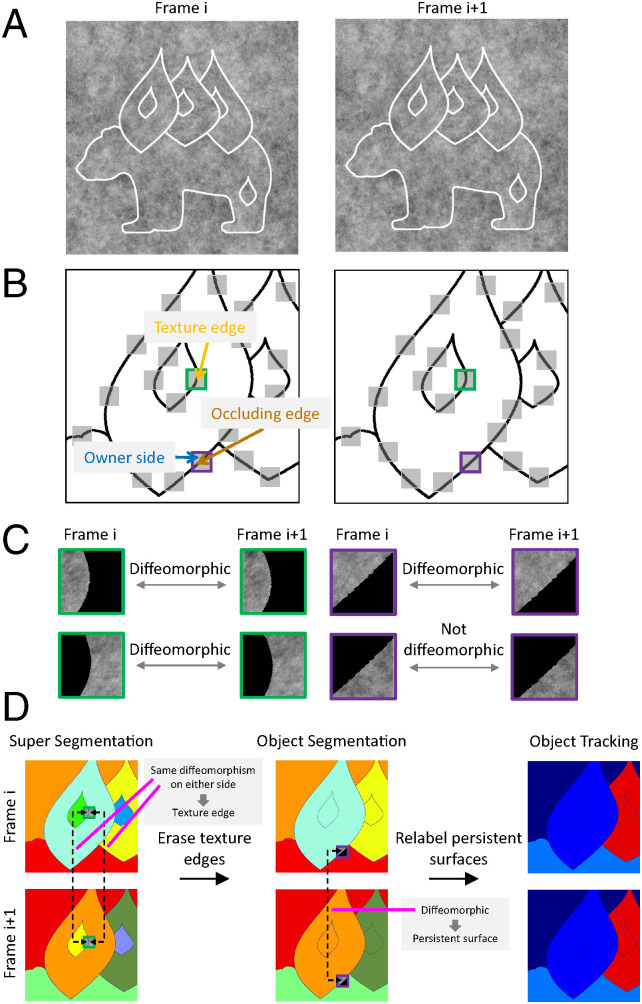
A computational implementation of topological segmentation and tracking. (*A*) A scene composed of four objects (a bear and three leaves) viewed from two neighboring observation points (frame i, frame i + 1); each object contains an internal texture contour. (*B*) Edge map corresponding to *A*, enlarged over one part of the image. The raw edge map includes both texture and occluding edges. To distinguish texture from occluding contours, we first randomly select a set of neighborhoods of edge elements (gray squares). (*C*) For each neighborhood, local diffeomorphism detection is independently performed on the left and right sides between successive frames. Existence of the same diffeomorphism on both sides implies a texture edge (left, green), while existence of a diffeomorphism on only one side (right, purple) implies an occluding edge; moreover, the owner side is specified by the side possessing diffeomorphism (here, the side to the left of the edge). (*D*) Workflow for computing object segmentation maps and object tracking maps. (*Left*) Super segmentation map for frames i and i + 1 assigning a different label to each contour-bounded component (35); note that texture edges and object edges are treated the same at this stage. (*Middle*) Object segmentation maps produced by identifying and erasing texture edges by resetting the label of any pure texture region (i.e., a region that abuts a texture edge but is never a one-sided owner) to that of its two-sided partner. (*Right*) Object tracking maps computed by determining persistent surfaces (i.e., components of the object segmentation map in frame i + 1 containing a patch diffeomorphic to a one-sided owner/texture patch from frame i) and assigning them the same label as that in the previous frame.

We first find intensity edges using a standard edge detection algorithm, for example, the Canny edge detector (32) (Fig. 4*A*); here, we are assuming that, in natural viewing conditions, occluding contours are mostly associated with intensity edges. This assumption is due to the fact that images of borders between spatially separated surfaces likely have different intensities. We then randomly select a set of neighborhoods of the identified edges for further topological analysis. Importantly, these neighborhoods are taken in pairs from successive frames (Fig. 4*B*). The next, crucial step is to classify edge segments as texture edges or occluding edges, based on diffeomorphism detection between successive frames performed separately on each side of the segment (Fig. 4 *B*–*D*), and then to identify the owner of each occluding edge. Following the mathematical theory, at texture edges, diffeomorphisms computed on either side are the same, while at object edges, the neighborhood on the side that owns the edge is diffeomorphic to its counterpart in the next frame, but the neighborhood on the opposite side is not due to accretion/deletion. The specific method we use to determine existence/nonexistence of diffeomorphism is described in detail in the next section as well as in *SI Appendix*, section 4 and in refs. 33 and 34.

Once texture edges have been distinguished from object edges, owners of object edges have been identified, and diffeomorphisms have been computed between successive frames at each neighborhood, we are then ready to perform object segmentation and tracking. We start by computing a “super segmentation” map that assigns a different label to each contour-bounded component (35) (Fig. 4 *D*, *Left*). Then, to compute the segmentation map, we simply erase texture edges by reassigning the label of any pure texture region (i.e., a region that abuts a texture edge but is never a one-sided owner) to that of its two-sided partner (Fig. 4 *D*, *Middle*). Finally, once segmentation is complete, the last step of computing the object tracking map becomes trivial: We determine persistent surfaces—components of the object segmentation map containing a patch diffeomorphic to a one-sided owner or texture patch from the previous frame (Fig. 4 *D*, *Middle*)—and assign each persistent surface the same label as that in the previous frame (Fig. 4 *D*, *Right*). Note that here, we are reusing the diffeomorphism detections performed during the segmentation stage.

In broad terms, the steps for scene segmentation and tracking just presented can be organized into three major groups of steps: 1) edge extraction and computation of a super segmentation map, 2) computation of diffeomorphic correspondence, and 3) relabeling of components of the super segmentation map using correspondence information. Below, we elaborate on the key computational workhorse in this scheme, detection of diffeomorphisms.

### 3.2. Extraction of Diffeomorphisms in a Perspective Transition.

Distinguishing texture from object edges requires the determination of existence/nonexistence of diffeomorphism. A diffeomorphism *g* can locally be approximated by its first-order Taylor expansion, which is a shift of the center point of the domain and a linear correction term for points in its vicinity, an affine transformation with six parameters. If the diffeomorphism is a stereo diffeomorphism (i.e., a diffeomorphism arising from viewing the same rigid object from two perspectives), then the transformation is constrained to only three parameters (*SI Appendix*, section 2.3c). Let *p* be a ray from the ray space *S*(*O*) and *p*′ *= g*(*p*) be its image in *S*(*O*′). Let *U*(*p*) and *U*′(*p*′) denote the set of all rays in local neighborhoods of *p* and *p*′, respectively, and $f_{O}$ and $f_{O\prime }$ denote functions that map each ray at *O* and *O*′, respectively, to a brightness value. The image patches taken at two locations *O* and *O*′, $f_{O}(U(p))$ and$\;f_{O\prime }(U\prime (p\prime ))$, are said to be *g-*related if

[1] p′=g(p),U′(p′)=g(U(p))=g∘U(p),

and

[2] $$f_{O}(U(p))=f_{O\prime }(g\circ U(p)).$$

Our goal is to compute, for any two image patches $f_{O}$ and $f_{O\prime },$ whether there exists a six-parameter affine transform *g* that satisfies Eq. **2**. If so, then we conclude that the two image patches are related by diffeomorphism *g*. Eq. **1** expresses the fact that light rays projected from a surface patch to ray spaces at two observation points are related by diffeomorphism (Fig. 3*B*), while Eq. **2** expresses the brightness constancy constraint, namely, that the brightness of every light ray originating from the same point is the same (we only need this to hold locally).

Our general approach is as follows: We project both image patches onto a set of Gabor receptive fields of varying orientation and spatial frequency. Importantly, we make these receptive fields dynamic, such that they can undergo affine transforms. We then set up an energy minimization process to find the affine transform of the receptive field that exactly cancels the affine transform of the image patch. If we succeed, then we conclude the two image patches are related by diffeomorphism, and the affine approximation to this diffeomorphism is given by the parameters of the identified receptive field transform. For example, if image patch 2 is shifted relative to image patch 1, then our energy minimization process identifies the precise amount of shift in receptive field such that

〈receptive field, image patch 1〉=〈shifted receptive field, image patch 2〉.

Formally, the real-valued function $f_{O}(U(p))$ on the image plane (i.e., the image patch) can be thought of as a vector in an infinite dimensional Hilbert space (i.e., a complete space with an inner product), and Eq. **2** is an abbreviation of an infinite system of equations. Given image patch $f_{O\prime }(U(p))$, the trajectory of $f_{O\prime }(g\circ U(p))$ in the Hilbert space of images on the second image plane under the affine Lie transformation group is a 6D submanifold in the Hilbert space. The power of considering an image patch as a vector in Hilbert space is that we can then represent an affine transform of the image patch as a conjugate affine transform in a dual vector space of differentiable receptive field functions. This allows us to use a gradient-based optimization approach to identify the conjugate affine transform that exactly cancels the affine transform of the image patch.

Projecting $f_{O}(U(p))$ on a subspace spanned by *n* differently oriented Gabor “receptive field” functions *F_i_*(*U*), *i* = 1, 2, *…, n* gives smoothed and band-pass filtered vector-valued signals,

$$\gamma_{O}^{i}(p)=\langle F_{i}(U),f_{O}(U(p))\rangle ,i=1,2,\ldots ,n.$$

Eq. **2** implies

[3] $$\gamma_{O}^{i}(p)=\langle F_{i}(U),f_{O}(U(p))\rangle =\langle F_{i}(U),f_{O\prime }(g\circ U(p))\rangle ,\;i=1,2,\ldots ,n.$$

The *n*-tuple ${\vec{\gamma }}_{O}(p)=(\gamma_{O}^{1}(p),\gamma_{O}^{2}(p),\ldots ,\gamma_{O}^{n}(p))$ in the *n*-dimensional signal space $R^{n}$ is called a Gabor place token. Notice the pullback via mapping g:U→U′ and g:(x,y)↦(x′,y′), $T(g):f\prime \mapsto f,f\in L^{2},f\prime \in L^{2}$, where $\left(T(g)\circ f_{O\prime })(U(p))=f_{O\prime }(g\circ U(p)\right)$ is a linear transformation on the Hilbert space of *L*^2^ functions. Let $T^{*}(g)$ be the conjugate of the Hilbert space transformation of T(g) with respect to the *L*^2^ inner product (see *Methods*); from Eq. **3**, we have

$$\begin{array}{c} \langle F_{i}(U),f_{O}(U(p))\rangle =\langle F_{i}(U),(T(g)\circ f_{O\prime })(U(p))\rangle ,\;i=1,2,\ldots ,n.\\ \langle F_{i}(U),f_{O}(U(p))\rangle =\langle \left(T^{*}(g)\circ F_{i})(U),f_{O\prime }(U(p)\right)\rangle ,\;i=1,2,\ldots ,n. \end{array}$$

Let $g^{*}$ be the image domain affine transformation with $T^{*}(g)$ as its pullback image transformation,

[4] $$\langle F_{i}(U),f_{O}(U(p))\rangle =\langle F_{i}(g^{*}\circ U),f_{O\prime }(U(p))\rangle ,\;i=1,2,\ldots ,n.$$

Let $g=g(\vec{a})$ be an affine transformation of six parameters $\vec{a}=(a_{11},a_{12},a_{21},a_{22},t_{x},t_{y})$ at location *p*, and define

$$\gamma_{O\prime }^{i}\left(p,\vec{a}\right)=\langle F_{i}\left(g^{*}\circ U\right),f_{O\prime }\left(U\left(p\right)\right)\rangle ,\;i=1,2,\ldots ,n.$$

Eq. **4** implies that the Gabor place token at point *p* is invariant to affine transformation in the sense that the Gabor place token extracted by conjugate affine-distorted Gabor receptive fields from the affine distorted image at a place on an image, $\;\gamma_{O\prime }^{i}\left(p,\vec{a}\right)$, equals the Gabor place token at the same place, $\gamma_{O}^{i}(p)$. Thus we can define an energy function

$$E(\vec{a},O,O\prime )=||{\vec{\gamma }}_{O\prime }(p,\vec{a})-{\vec{\gamma }}_{O}(p)|{|}^{2}.$$

**Fig. 5.**
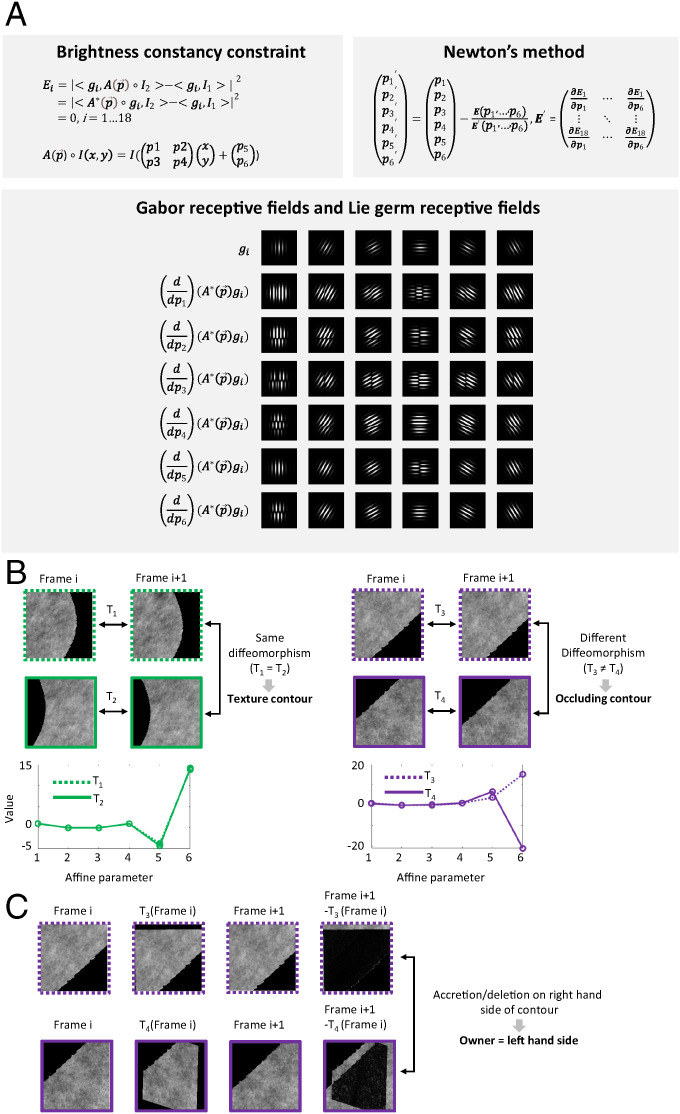
Computing diffeomorphisms. (*A*) To compute the diffeomorphism between two patches centered at a point, we project two image patches onto a set of Gabor receptive fields *g_i_* (*i* = 1,…18 for six orientations and three spatial frequencies). (*Top Left*) Due to the geometry of perspective projection and the brightness constancy constraint, the two image patches will be locally related by an affine transform, $A(\vec{p})$, corresponding to the first term in the Taylor series expansion of the full diffeomorphism; this yields the equation for $E_{i}$ shown. To compute this transform, we solve for parameters $\vec{p}$ such that ***E*** = 0. (*Top Right*) We do this using Newton’s method, which requires computing the derivative matrix E′. (*Bottom*) This, in turn, requires computing derivatives of the Gabor receptive fields with respect to each parameter of the affine transform, dubbed “Lie germ receptive fields” (34). (*B*) A pair of image frames from a point centered on a texture contour (*Left*, green) and a point centered on an occluding contour (*Right*, purple); these are the same two neighborhoods shown in Fig. 4*C*; here, the center of the patch has been shifted to the left (*Top*) or right (*Middle*) in order to provide a sufficient support for affine transform computation. (*Bottom Left*) The six parameters of the affine transforms *T*_1_ and *T*_2_ computed between frames i and i + 1 for the left and right neighborhoods, respectively, are plotted. They are equal, implying that the contour separating the two neighborhoods is a texture contour. (*Bottom Right*) The same computation at a different edge point yields affine transforms *T*_3_ and *T*_4_ for the left and right neighborhoods; these are different, implying that the contour separating the two neighborhoods is an occluding contour. Note that computing a difference in diffeomorphism between the two sides is equivalent to computing a diffeomorphism breakdown on one side, but is computationally easier, since it does not depend on detecting nonconvergence of Newton’s method. (*C*) At occluding contours, the foreground side owns the contour. To determine the owner, we apply the affine transforms computed for the left- and right-hand sides (*T*_3_ and *T*_4_, respectively) to the left and right parts of the image patch in frame i (column 1), to produce transformed image patches (column 2). We then compare these to the left and right parts of the image patch in frame i + 1 (column 3). For the owner side, these should be identical (columns 2 and 3, *Top*), while, for the occluded side, there should be a border of accretion/deletion leading to difference (columns 2 and 3, *Bottom*). Here, this process reveals a border of deletion to the right of the contour (column 4, *Bottom*), implying that the owner is to the left (see Fig. 4*B* for zoomed out view of the patch). Note that differences in column 4 are projected onto Gabor receptive fields; thus differences at the edges are discounted.

Since this energy function is an analytical function of the affine parameters $\vec{a}=\left(a_{11},a_{12},a_{21},a_{22},t_{x},t_{y}\right)$ defining g, we can solve for $\vec{a}$ using a gradient dynamical system (Fig. 5*A*).

Images taken from different views are almost always subject to compounded distortion involving rotation, scale, and skew, and our method is the only one we know of that can handle such compounded distortion in a principled manner; in contrast, the popular SIFT (scale invariant feature transform) approach of Lowe (36, 37) can only handle scale and orientation changes. Other correspondence methods such as FlowNet (38) generate a dense optic flow map at each point and do not directly inform about the existence of a diffeomorphism within a local neighborhood.

Equipped with this dynamic receptive field method for extracting diffeomorphisms, we can readily distinguish texture from occluding edges and identify the owners of the latter, using the fact that the six affine parameters extracted from computing correspondence between the left and right sides of a texture edge are identical (Fig. 5 *B*, *Left*), while those extracted from computing correspondence between the left and right sides of an occluding edge are different (Fig. 5 *B*, *Right*). Moreover, we can readily identify the owner of the occluding edge by determining which side is diffeomorphic to its counterpart in the next frame (Fig. 5 *C*). Overall, our method for diffeomorphism detection provides a principled way to compute the key signal necessary for topological segmentation and tracking, surface correspondence. From these local correspondence signals, surfaces can then be stitched together across space and time to endow the visual world with global, symbolic structure.

### 3.3. Results on Synthetic Video Containing Severe Appearance Changes due to Object Deformation, Changing Perspective, and Dynamic Occlusion.

**Fig. 6.**
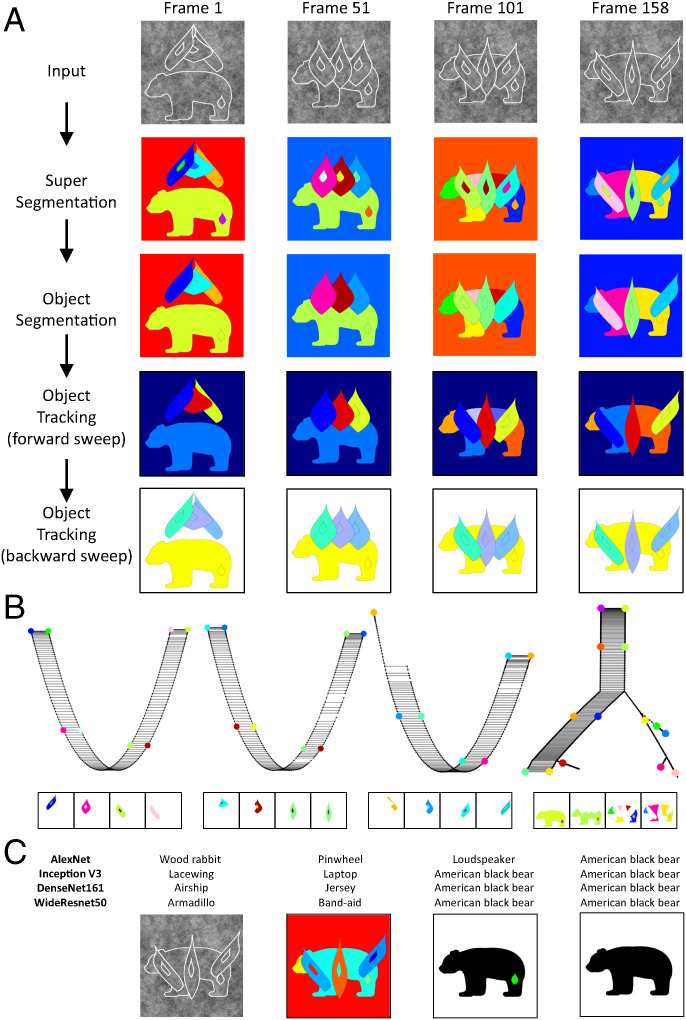
Segmenting and tracking objects in a synthetic dataset containing multiple objects despite severe appearance changes due to object deformation, changing perspective, and dynamic occlusion. (*A*) The output of the segmentation and tracking system after each stage of processing (see Fig. 4*D*). *Row 1* shows input images at four distinct time points. *Row 2* shows super segmentation maps. *Row 3* shows object segmentation maps. *Row 4* shows object tracking maps. *Row 5* shows revised object tracking maps computed via a backward sweep after computation of invariant object graphs. (*B*) *Top:* Four connected components of the scene graph computed from this synthetic dataset, corresponding to the three leaves and the bear. Each vertex corresponds to a distinct super segmentation component. Vertices of each graph component corresponding to the frames shown in *A* are indicated by color. *Bottom:* The corresponding super segmentation components are reproduced in the frames below each graph component. Note how tracking is robust to severe changes in shape due to object deformation, changing perspective, and dynamic occlusion. (*C*) Four images from a single frame, taken from different processing stages in the topological segmentation and tracking workflow: (*Left* to *Right*) visual input, super segmentation map, tracked surface component with texture patch distinguished, and tracked surface component with texture patch removed. The corresponding classification of each image by four different deep networks is indicated above. Through topological segmentation and tracking, the cluttered input image (*Left*) can be transformed/linked to an unoccluded representation of an isolated surface.

To test our system, we generated a video sequence consisting of 160 frames of a dynamic scene with four objects. The objects underwent severe deformation, perspective change, and partial occlusion, and furthermore, each contained an internal texture contour to challenge the segmentation process (see https://youtu.be/eu_aJNo3R5I for a movie of the stimulus sequence). Fig. 6*A* shows the results of our topological approach applied to this dataset: We are readily able to segment and track the four objects despite drastic appearance changes.

Following a feedforward sweep across all frames, we obtain a complete scene graph whose vertices comprise super segmentation components across space/time and whose edges correspond to connectedness between these surface components across space/time. The distinct components of this scene graph correspond to distinct invariant objects (Fig. 6*B*). Equipped with this scene graph, we can then retraverse the frames and assign the same label to each surface in the segmentation map that belongs to the same connected component in the scene graph. This allows distinct surface components to be identified as part of the same object across splits and joins over time (Fig. 6 *A, Row 5*).

We underscore the severity of appearance changes that our method can handle (Fig. 6 *B*, *Bottom*). This is possible because information for grouping is not tied to image features within single frames but rather to topological relationships between successive frames. Moreover, the approach is robust because information for surface representation is redundantly available on a massive scale: 1) The number of distinct objects in the environment is much less than the number of local neighborhoods available for diffeomorphism detection, and 2) most objects persist over time. Assuming the occluding contour of a typical object runs through 100 neighborhoods, and persists through 100 stimulus frames (i.e., 1 s for a 0.1-kHz visual system), this would generate 10,000 independent diffeomorphism measurements; in our simulation, we found that ∼5 diffeomorphism measurements were sufficient to correctly segment a surface component. Thus, even though our demonstration was for synthetic video of textured surfaces observed without noise, it is not implausible that the approach could be adapted to natural video where these assumptions no longer hold.

In our computational test, in which 100 neighborhoods per frame were sampled across 160 frames, the segmentation process made a total of 13 mistakes (representing an error rate of 2%, since, if one simply copied the super segmentation map as the segmentation map, this would result in 640 errors). *SI Appendix*, Fig. S1 presents a detailed analysis of one such mistake. An internal texture component was incorrectly segmented as a separate unit in one frame (*SI Appendix*, Fig. S1 *A* and *B*), but was nevertheless correctly tracked due to correct surface connectivity information across frames (*SI Appendix*, Fig. S1 *A* and *C*). Indeed, in the same way, all 13 segmentation mistakes vanished after object tracking. This illustrates how redundancy in information for surface representation leads to robustness.

We conclude this section on computational results with a simple demonstration of how our topological surface representation mechanism could significantly augment the capabilities of current deep neural networks trained to classify objects. Such networks rely heavily on texture (40) and can be fooled by small amounts of strategically placed noise imperceptible to humans (41). Furthermore, they are highly sensitive to training distribution (42). Indeed, if we take four images corresponding to different stages of tracking, which each carry different color/texture information, and present them to various deep networks trained to classify images, we get three or four different answers (Fig. 6*C*; to the human eye, it is evident all four frames contain a bear with varying amounts of occlusion and varying surface color/texture); only one, in response to segmented and tracked input, is reliably correct. Thus, through topological segmentation and tracking, we can transform cluttered visual input that is unrecognizable to a classification-trained deep network (first three images in Fig. 6*C*) into a representation of object surfaces that is readily recognizable (fourth image in Fig. 6*C*).

## 4. Discussion

The essential conceptual advance of this article is to show how generation of a visual surface representation turns the problem of segmentation and invariance from an ill-posed challenge, requiring ad hoc tricks or black box deep learning, to a readily solvable problem. The world is composed of objects possessing smooth textured surfaces, and animals view the world from a moving observation point. With only these two natural assumptions, we proved it is possible to solve the problem of segmenting and invariantly tracking each discrete surface in the environment. Our theory explains how a surface representation, that is, a topological labeling of contiguous surface components together with a geometric description of their shapes and positions, can be extracted from perspective projections of the environment in a manner that is invariant to changing perspective and occlusion. We prove that segmentation of an image into separate surfaces can be accomplished through detection of occluding contours (which carry information about spatial separation of visible surfaces), and tracking of invariant surfaces in an image sequence can be accomplished by detection of diffeomorphisms (which carry information about overlap relations between surfaces visible from different views). Furthermore, we not only prove the validity of our approach mathematically but demonstrate its computational efficacy for object segmentation and invariant tracking of synthetic video.

It is a common belief that, in an image, there is, in reality, no occlusion, no surface, no contour, but only an assemblage of pixels, and the goal of perception is to “interpret” these sensory data. Our work shows how the visual system can perceive topological structures (occlusions, surfaces, contours, etc.) in a true and original sense. The perception of these topological structures does not require observer-dependent interpretations but can result from extraction of information directly specifying these topological objects and their relations in a rigorous mathematical sense. To achieve this, it is necessary to expand the concept of perspective projection. Perspective projection is generally considered as a mapping from a point in 3D space to a point in the image plane. However, to understand segmentation and invariant tracking of real, curved objects, we show that it is essential 1) to regard perspective projection as a mapping from a 2D surface of an object to a 2D ray space, and 2) to further enlarge the focus, from how a 2D surface is projected to a single ray space to how it is projected to a field of ray spaces. This mathematical construction enables us to use differential topology to reach statements about surfaces as global entities: Perspective projections are now 2D to 2D diffeomorphisms on regular domains, which are separated by critical points that take the form of fold contours, and these critical points are encoded by accretion/deletions in mappings between neighboring ray spaces. Without this construction, we can only speak of points.

Our theory was presaged by Gibson’s theory of surface perception (20). Gibson observed that surface contiguity is specified by order-preserving transformations (“the available information in the optic array for continuity could be described as the preservation of adjacent order”), and related occluding contours to accretion/deletion events (“It is called a transition, not a transformation, since elements of structure were lost or gained and one-to-one correspondence was not preserved…Deletion always caused the perception of covering, and accretion always caused the perception of uncovering”). Nakayama et al. (43) further developed the concept of surface representation and incisively demonstrated its importance to human vision through ingenious psychophysical experiments. In particular, they discovered the astonishing psychophysical phenomenon that accretion/deletion in stereograms is sufficient to produce the percept of surface separation. They termed this form of 3D perception “da Vinci stereopsis,” to contrast it with “Wheatstone stereopsis,” which concerns the perception of the depth of binocularly visible points (23, 43). Both da Vinci and Wheatstone stereopsis have been formulated in terms of matching points in a pair of images. But the problems of segmentation and object tracking essentially require grouping of neighborhoods of points. Thus, to make these two problems mathematically and therefore computationally tractable, we had to replace the geometric optics used to explain da Vinci and Wheatstone stereopsis with an ecological optics based on differential topology.

These topological concepts from ecological optics shine new light on many classic ideas in vision research. For example, an occluding contour is typically regarded as an intensity discontinuity due to a surface 3D distance discontinuity. Our definition, on the other hand, does not even involve “intensity.” In our framework, an occluding contour is simply a singularity in the perspective projection, with the associated property of being an infinitesimal accretion border; this concept of occluding contour lies at the foundation of our formulation of image segmentation. As another example, invariance has conventionally been regarded as an issue related to object learning. In our framework, invariance is mathematically formulated as an equivalence relation between perspective images of surfaces; the critical equivalence relation is surface overlap, and the machinery for computing equivalence is local diffeomorphism detection. Importantly, this mathematical formalism carries with it enormous computational power, which we discuss next in relation to computer vision.

### 4.1. Implications for Computer Vision.

The theory of topological surface representation has significant implications for computer vision. The theory underscores the importance of equipping artificial vision systems with an explicit surface representation intermediate between pixels and object labels. Furthermore, the theory clarifies that surface overlap is the key mathematical property enabling object tracking. In contrast, most computer vision algorithms for tracking assume that the tracked object should be “similar” between frames (with “similar” defined in various ad hoc ways).

Current computer vision methods for video segmentation can be broadly divided into three approaches. One approach (“tracking by detection”) relies on first segmenting individual objects within single frames and then linking the segmented object instances across frames via some similarity measure (10, 44–47). The fundamental insufficiency of tracking by detection as an account of human perception was recognized by Bela Julesz (48) more than 60 years ago: Human perception of physical reality is first and foremost determined by perspective transformations between images and not by forms within single images (*SI Appendix*, Fig. S2). A second approach attempts to perform video segmentation by directly using optical flow as input (11, 12, 15, 49–52). Finally, a third approach in the era of deep learning is end-to-end trained deep networks that take video as input and output per-frame object detections (e.g., refs. 53 and 54).

While some of these computer vision approaches have kinship to the theory of topological surface representation presented here, their implementations often rely on 1) ad hoc assumptions (e.g., that objects constitute clusters of pixels with similar motion patterns, which is invalid for nonrigid objects) or 2) black-box deep learning approaches that do not leverage the principles enabling optical flow to generate object labels. Nevertheless, existing approaches have achieved impressive performance on benchmarks for tracking objects in real-world video (10, 52, 55, 56) and gained valuable insights into how to incorporate learning to build robust segmentation and tracking systems (10–12, 15). We believe such systems may become even more powerful by incorporating a mathematically grounded surface representation framework ab initio. Below, we give four specific arguments why this is advantageous.

### 4.1.1 Surface Representation Clarifies What Needs to Be Learned.

Ecological optics breaks the problem of object perception into two halves: 1) how surfaces in space are projected into ray spaces and how the diffeomorphisms and breakdowns in diffeomorphisms within the ray spaces encode the surfaces and 2) how to compute these diffeomorphisms from images. The first half of this problem is a mathematically exact encoding problem. The second half is a detection problem, which faces issues of noise and ambiguity. The conceptual insight is that the first step greatly simplifies the problem of vision. The organization of a scene into surfaces is defined by a 1D set of occluding contours, and information to detect these contours is highly redundantly available through movements of the observation point, making the detection problem readily solvable (as demonstrated by the fact that, in real life, we actually do not encounter many ambiguous visual situations).

We do not underestimate the magnitude of the second half of the problem and the amount of engineering necessary to transform our current algorithm, which works on synthetic video without any noise, into a robust system that works on real-world data. For this purpose, learning will almost certainly be essential: to generate high-quality super segmentation maps that provide the essential input for our system, to handle objects that lack enough pixels to compute border ownership at edges accurately (e.g., thin shapes like chair legs), and, most importantly, to intelligently combine local signals about surface organization into a coherent scene narrative. This last task will require knowledge of natural scene statistics to add breaks or links to the object graphs computed by the bottom-up surface segmentation and tracking mechanism [e.g., for the purpose of “reidentification” (57), in which object identity is preserved even after complete occlusion]. Importantly, surface representation vastly simplifies the problem, since statistical knowledge supporting inference can now be expressed in terms of surfaces, which constitute a low-dimensional symbolic representation (Fig. 6*B*).

While it is certainly the case that our system cannot handle real-world video without further engineering, it is equally the case that existing segmentation and tracking systems have fundamental insufficiencies compared to our system, and to the human visual system: We note that, if we apply a recent multiobject tracking system to our synthetic video, the results are extremely disappointing (*SI Appendix*, Fig. S3) (58).

### 4.1.2 Surface Representation Enables Self-Supervised Learning of Object Recognition from Spatiotemporal Contiguity in a Principled Manner.

An influential conceptual framework for object recognition suggests that it constitutes a process of manifold untangling (26), with the essential challenge to untangle tangled manifolds. We suggest that there is an even more fundamental and prior challenge: finding connected paths along distinct tangled manifolds. This is precisely what topological surface segmentation and tracking accomplishes. The theory makes the concept of “spatiotemporal contiguity,” which has previously been suggested to play an important role in unsupervised learning (59–61), precise. For example, one technique used for the latter is contrastive learning of image views, in which a network learns to make the representations of two different views of the same scene agree and the representations of two views of different scenes disagree (62, 63). However, as noted by Hinton (64), for a scene with multiple objects, one does not want to learn a representation that makes the entire scene in one frame similar to the entire scene in the next frame; rather, one wants to encourage similar representations only for representations of the same objects. Topological surface representation provides machinery to achieve this: The tracking mechanism provides a large set of labeled object examples (Fig. 6 *A*, *Row 5*) to pretrain a subsequent invariant recognition module in a self-supervised manner. Thus a visual system initially equipped with diffeomorphism-based surface representation machinery can learn much more effectively than a tabula rasa. Moreover, after learning to extract a surface representation using other cues besides local diffeomophism, the visual system can then readily handle situations where topological surface segmentation and tracking would encounter difficulties, such as non-Lambertian lighting.

### 4.1.3 Surface Representation May Benefit from Specialized Front-End Hardware.

Our topological solution to segmentation and tracking depends on accurate computation of correspondence. While recent emphasis in building intelligent vision systems has focused on developing better learning algorithms and more powerful datasets, for solving the correspondence problem, faster front-end hardware could also make a critical difference. In particular, “event-based” cameras built on the principles of biological retinas to detect changes can operate at an effective frame rate of ∼50 kHz rather than the typical video rates of 30 Hz, while maintaining low sensing and computing throughput (65). Such cameras would make the correspondence problem significantly easier, due to smaller changes from frame to frame and elimination of image blur. Artificial vision systems thus equipped could exploit topological surface representation with maximal efficiency. Together with parallelization of correspondence detection using GPUs (66), we envision that our segmentation and tracking system could operate in real time.

### 4.1.4 Surface Representation Unifies Segmentation, Tracking, and 3D Surface Reconstruction into a Coherent Framework.

In computer vision, object segmentation/tracking and 3D surface reconstruction have largely been pursued through distinct paths (for review of the latter, see ref. 67). In the current paper, we have focused on the former: how diffeomorphisms computed at object edges enable identification of occluding contours and stitching of overlapping surface patches over time. Importantly, a diffeomorphism specifies not only the existence of a contiguous surface patch but also its metric properties of distance and curvature (*SI Appendix*, Shape from Perspective Mapping Theorem). Thus the same machinery for diffeomorphism computation, when carried out across the image, should enable accurate surface reconstruction.

## 4.2 Implications for Biological Vision

We believe our results have important implications not only for building new artificial vision systems but also for understanding biological vision. We currently possess detailed understanding of neural mechanisms for very early image processing such as edge detection (68) and motion detection (69), as well as mechanisms for very high-level object recognition such as face recognition (70). What is missing are the steps in between, which explain how an object first arises: how a set of edges can be transformed into a set of object contours invariantly associated with specific objects. The solution to this fundamental problem presented here outlines a path for neuroscience research to go beyond the search for simple neural correlates of perceptual grouping, to discover the detailed workings of visual surface representation.

The computations we describe for solving segmentation and invariant tracking are necessarily local and therefore likely accomplished in retinotopic visual areas. The invariant label for each object is propagated across the object through local diffeomorphisms between different perspectives—local threads (the edges of the scene graph, Fig. 6*B*) create global objects (the connected components of the scene graph and their associated symbolic labels). We believe purely local processes in retinotopic visual areas must generate a representation akin to an object graph, and this object graph structure must already be in place by the output stage of a retinotopic visual area [possibly area V4 or a retinotopic region within the posterior parietal cortex (71)]. To create an object graph, an essential neural mechanism is needed to represent the linkages within the graph. What this binding signal consists of remains unknown and constitutes, in our view, one of the biggest known unknowns in systems neuroscience. Notably, a recent study suggests that the machinery for invariant visual surface representation may be unique to primates (72). One piece of physiological evidence for the existence of topological surface representation in the primate brain is the finding of “border-ownership cells” that show selectivity for side-of-owner of contours (73), a critical topological feature which we show how to compute (Fig. 5*C*). Our theory predicts that the output of border-ownership cells should be integrated over time to generate invariant object labels (Fig. 4*D*), effecting the fundamental transformation of visual information from sensory to symbolic.

The theory of ecological optics presented here is not an arbitrary new model of vision but a mathematical necessity. And each part of the theory maps onto a computational goal and mechanism. The essential simplicity and necessity of the theory set a new direction for vision research to understand in detail how surface representation is accomplished in the brain.

## 5. Methods

Detailed methods for generating the synthetic dataset and computational implementation of topological segmentation and tracking are described in *SI Appendix, Supplementary Methods*.

### 5.1. Generating a Synthetic Dataset.

To perform the computational test, we generated a synthetic 160-frame video sequence by applying four independent sequences of affine transforms to each of four objects. See https://youtu.be/eu_aJNo3R5I for a movie of the stimulus sequence.

### 5.2. Computational Implementation of Topological Segmentation and Tracking.

We began by identifying a set of neighborhoods in each super segmentation frame. Then, at each point, on each side of the edge, we computed the affine transform between successive frames. From these measurements, we built segmentation (Fig. 6*A*, row 3) and tracking (Fig. 6*A*, rows 4, 5) maps following the approach illustrated in Fig. 4*D*.

### 5.3. Dynamic Receptive Field Method for Diffeomorphism Extraction (33, 34).

At each point, for each side of the edge, we projected the input image from successive frames onto Gabor receptive fields. We then used Newton’s method with 10 iterations to find the value of the six affine parameters $\vec{p}$ constituting the zero of the equation $E=\;\underset{i}{\sum }E_{i}\;$ (Fig. 5*A*).

## Supplementary Material

Supplementary File

## Data Availability

All code to reproduce the reported results can be found on GitHub at https://github.com/dortsao/CODE_TSAO_PNAS (74).
