# Supplementary material for: A topological solution to object segmentation and tracking
Source: Proc Natl Acad Sci U S A. 2022 Oct 6;119(41):e2204248119. doi: 10.1073/pnas.2204248119 (PMC9564096; doi:10.1073/pnas.2204248119)
Supplement: Supplementary File [file pnas.2204248119.sapp.pdf]

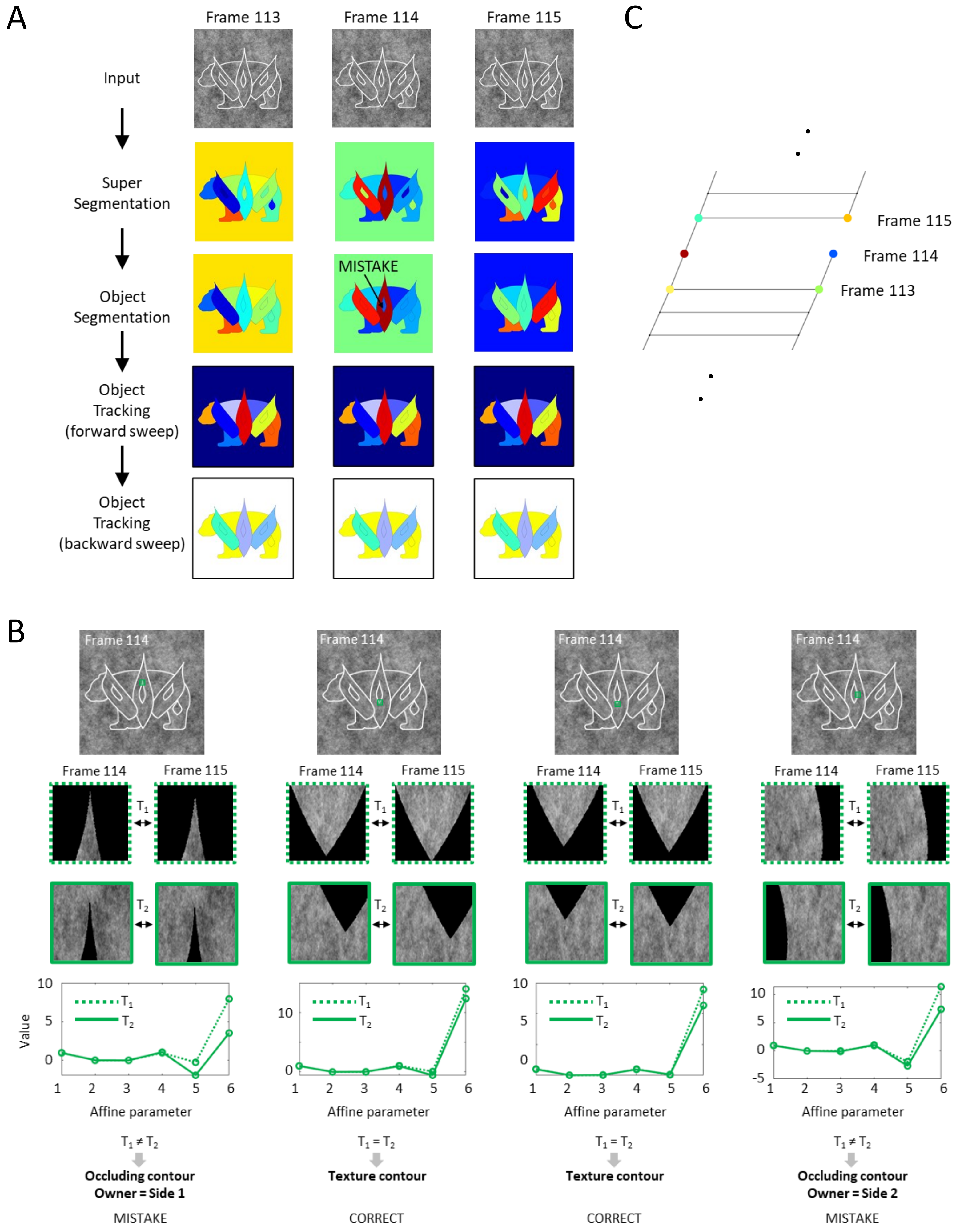
A
Frame 113
Frame 114
Frame 115
Input
Super Segmentation
Object Segmentation
MISTAKE
Object Tracking (forward sweep)
Object Tracking (backward sweep)
C
Frame 115
Frame 114
Frame 113
B
Frame 114
Frame 115
T1
T2
Value
Affine parameter
T1 ≠ T2
Occluding contour
Owner = Side 1
MISTAKE
T1 = T2
Texture contour
CORRECT
T1 = T2
Texture contour
CORRECT
T1 ≠ T2
Occluding contour
Owner = Side 2
MISTAKE

Figure S1

Figure S1. The segmentation and tracking process is highly robust due to redundancy of information for surface representation. (**A**) A frame in which a mistake is made in object segmentation, together with two adjacent frames. Conventions as in **Fig. 6A**. (**B**) Pinpointing the source of error: two-sided diffeomorphism detection was performed for neighborhoods of four edge points (top row) along the contour separating the inner from outer component of the middle leaf. Conventions as in **Fig. 5B**. Two of the four neighborhoods are correctly classified as containing texture contours (columns 2, 3), while two are mistakenly classified as containing occluding contours (columns 1, 4). (**C**) Portion of object graph corresponding to the segmentation mistake. The blue dot in frame 114 should be connected to the red dot in frame 114, since they are separated by a texture contour. However, due to mistakes in local diffeomorphism detection, the contour is misclassified in this frame as an edge contour, and thus the connection between between the red and blue dots in the object graph is missing. Nevertheless, due to a connection with the previous frame representing surface persistency (green -> blue), the super segmentation component represented by the blue dot remains connected to the correct global object graph, and the mistake is absent from the object tracking map for the same frame. This example illustrates how redundancy in information for surface representation leads to robustness.

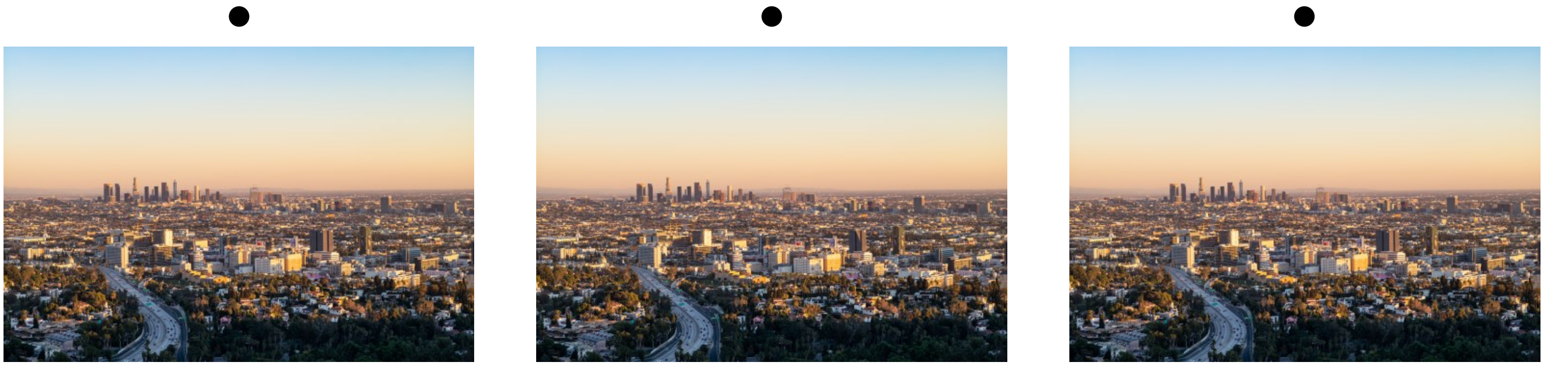

Figure S2

Figure S2. The primacy of perspective transformations for determining perception of physical reality. A stereogram of a bear is constructed from a texture pattern consisting of LA skyline. Tracking-by-detection approaches would attempt to segment the buildings, freeway, and other objects in the two frames and then track these and would thus completely miss the bear that pops out only through stereo.

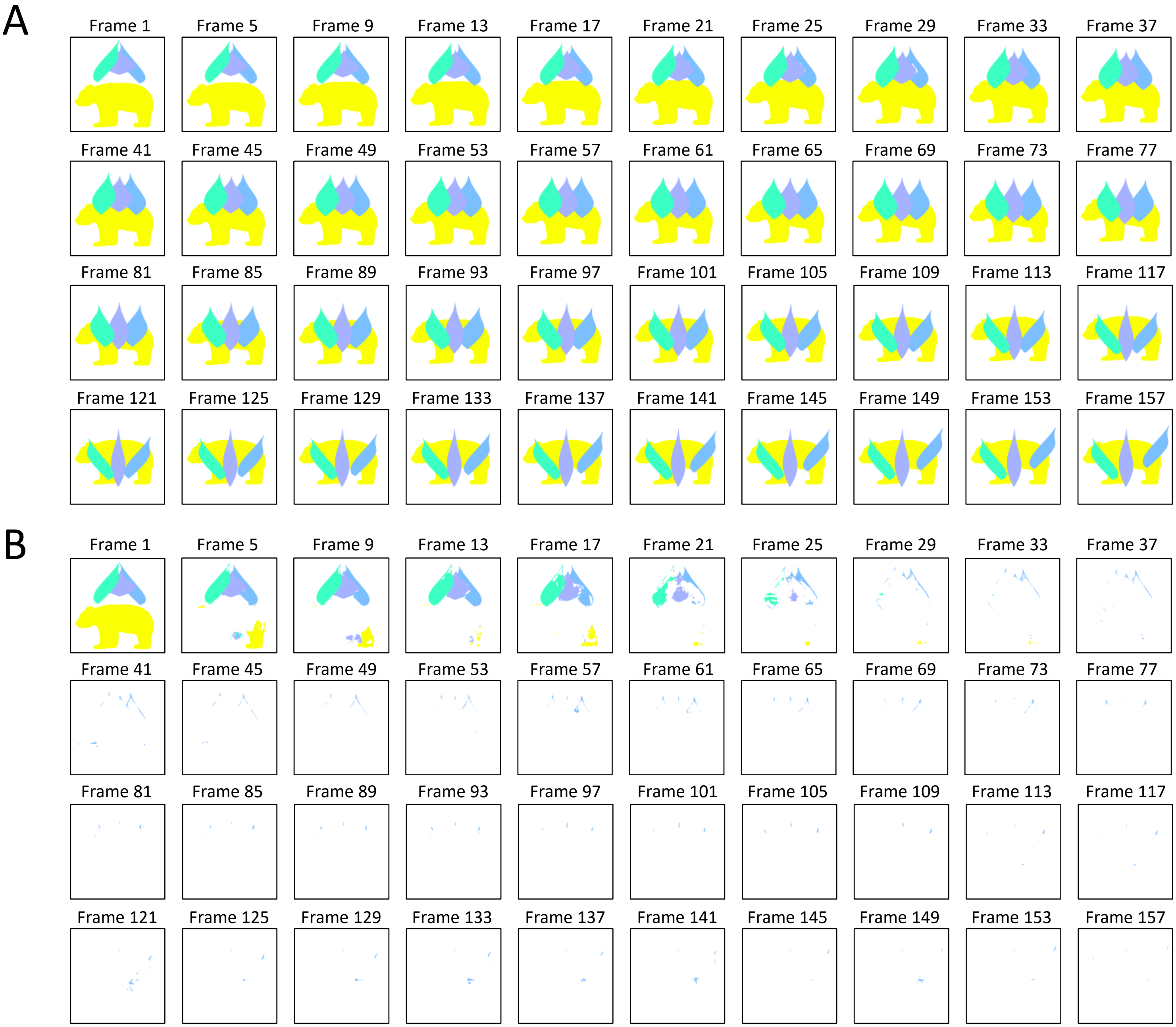
A
Frame 1
Frame 5
Frame 9
Frame 13
Frame 17
Frame 21
Frame 25
Frame 29
Frame 33
Frame 37
Frame 41
Frame 45
Frame 49
Frame 53
Frame 57
Frame 61
Frame 65
Frame 69
Frame 73
Frame 77
Frame 81
Frame 85
Frame 89
Frame 93
Frame 97
Frame 101
Frame 105
Frame 109
Frame 113
Frame 117
Frame 121
Frame 125
Frame 129
Frame 133
Frame 137
Frame 141
Frame 145
Frame 149
Frame 153
Frame 157
B
Frame 1
Frame 5
Frame 9
Frame 13
Frame 17
Frame 21
Frame 25
Frame 29
Frame 33
Frame 37
Frame 41
Frame 45
Frame 49
Frame 53
Frame 57
Frame 61
Frame 65
Frame 69
Frame 73
Frame 77
Frame 81
Frame 85
Frame 89
Frame 93
Frame 97
Frame 101
Frame 105
Frame 109
Frame 113
Frame 117
Frame 121
Frame 125
Frame 129
Frame 133
Frame 137
Frame 141
Frame 145
Frame 149
Frame 153
Frame 157

Figure S3

Figure S3. Comparison of segmentation and tracking results using our method and a recent multi-object tracking algorithm (73). (**A**) Tracking results for every fourth frame using our method. (**B**) Tracking results on the same synthetic video using a semi-supervised video tracking algorithm that uses a space time memory network. Tracking fails very early, likely because the network relies on interpolation within the training distribution instead of building a cue-invariant surface representation.

Supplementary Methods

Generating a synthetic dataset. To perform the computational test, we generated a synthetic 160-frame video sequence as follows: we started with an initial 5745 x 5745 image containing four objects bounded by bright contours, each possessing an additional internal texture contour to make segmentation challenging; the texture of both object surfaces and the background consisted of 1/f noise. We then applied four independent sequences of affine transforms to each of the four objects. See https://youtu.be/eu_aJNo3R5I for a movie of the stimulus sequence. In addition to this video sequence, we also generated a corresponding edge map and super segmentation map for each frame.

Computational implementation of topological segmentation and tracking. We began by identifying a set of neighborhoods in each super segmentation frame evenly sampling the different types of surface adjacencies (i.e., pairs of distinct, abutting components). To do this, in each frame, for each edge point, we computed four numbers (n, s, e, w) corresponding to the value of the super segmentation map 20 pixels above, below, to the left, and to the right of the edge point. We identified unique neighborhoods as unique combinations of (n, s, e, w). For each frame, we identified 100 random edge points that evenly represented different unique two-sided neighborhoods (i.e., neighborhoods containing just two super segmentation labels). Then, at each point, on each side of the edge, we computed the affine transform (using our dynamic receptive field method for diffeomorphism extraction) between frame i and frame i+1. The end result of this process is that for each frame, at each of 100 edge points, we have two sets of 6 affine parameters, $\vec{p}$ and $\vec{p}'$, corresponding to the affine transforms computed for the two sides of the edge. If the two are the same (determined by the criterion that $|(p_1 - p_1{}' \, p_2 - p_2{}' \, p_3 - p_3{}' \, p_4 - p_4{}')| < 0.1$ and $|(p_5 - p_5{}' \, p_6 - p_6{}')| < 4$), then we classified the edge element as a texture edge, otherwise we classified it as an occluding contour. For edge elements classified as occluding contours, we further determined the owner side by applying the affine transforms computed for the left- and right-hand sides to the left and right parts of the image patch in frame i, respectively, and comparing these to the left and right parts of the image patch in frame i+1 (**Fig. 5C**); comparison was done after projection onto Gabor receptive fields. The side with smaller difference was taken as the owner side. For each unique two-sided neighborhood type, we used winner-take-all across all neighborhoods of that type to determine the consensus classification.

Thus for each frame of the super segmentation map, each unique neighborhood type was classified as one-sided (i.e., occluding edge) or two sided (i.e., texture edge), and for occluding edges the owner side was identified. Furthermore, for i) the owner side of an occluding edge and ii) for both sides of a texture edge, the diffeomorphic super segmentation component(s) in the next frame were identified (**Fig. 4D**, column 2): these constitute the persistent surface components. In cases where a single component splits in two, such that the same surface component in frame i is diffeomorphic to two or more different target surfaces in frame i+1, we picked the label of the modal target in frame i+1 as the one that persists, while others were assigned new labels. Finally, we identified the background label in each super segmentation map as the region for which the difference between the pixel value in the current frame and that in the previous frame was most often 0.

To create the object segmentation map, for each frame, we started with the super segmentation map. We then ran through all the labels in this map, and if a label was part of two-sided neighborhood but was never a one-sided owner, then we reset its label to that of its two-sided partner. This effectively erases all texture edges (**Fig. 4D**, column 1 → column 2).

To create the backward object tracking map, for each frame, we started with the segmentation map. We then set the label of each surface identified as persistent to the label of that surface in the previous frame (**Fig. 4D**, column 2 → column 3). In addition, we set the label of the background to 0.

Finally, to create the post-hoc object tracking map (**Fig. 6A**, row 5), we first built, during the process of forward traversal through frames just described, a scene graph containing a node for every distinct component of the super segmentation map in every frame, and an edge between any two nodes identified as connected, either due to separation by a texture edge, or due to persistency across frames. We then identified the distinct components of this scene graph, and assigned a unique label to each; for our synthetic dataset, there were five labels in total, one for each of the objects plus one for the background. Equipped with this scene graph, we then re-traversed the frames, assigning to each component of the super segmentation map a new invariant object label according to its membership in one of the scene graph components.

Dynamic receptive field method for diffeomorphism extraction (33, 34). We projected the input image onto Gabor receptive fields defined on 200 x 200 image patch, at θ = 0, 30, 60, 90, 120, 150 and λ = 10, 20, 30:

$$g_i = e^{-\frac{x_\theta^2 + y_\theta^2}{2\sigma^2}} \cos\left(\frac{2\pi}{\lambda} x_\theta\right), \begin{pmatrix} x_\theta \\ y_\theta \end{pmatrix} = \begin{pmatrix} cos\theta & sin\theta \\ -sin\theta & cos\theta \end{pmatrix} \begin{pmatrix} x \\ y \end{pmatrix}, i = 1 \dots 18$$

We then used Newton's method (**Fig. 5A**) with 10 iterations to find the value of the six affine parameters $\vec{p}$ constituting the zero of the equation

$$E_i = \left|< A^*(\vec{p}) \circ g_i,\ I_2 > - < g_i, I_1 >\right|^2 \qquad (1)$$

where $A^*(\vec{p})$ is the conjugate of the affine transform $A(\vec{p})$,

$$A(\vec{p}) \circ I(x, y) = I(\begin{pmatrix} p_1 & p_2 \\ p_3 & p_4 \end{pmatrix} \begin{pmatrix} x \\ y \end{pmatrix} + \begin{pmatrix} p_5 \\ p_6 \end{pmatrix}).$$

The rationale for (1) is as follows. By the brightness constancy constraint,

$$E = 0 = | \iint \left[e^{\frac{-(x_\theta^2 + y_\theta^2)}{2\sigma^2}} \cos\left(\frac{2\pi}{\lambda} x_\theta\right)\right] \left[I2\big(u(x,y), v(x,y)\big) - I1(x,y)\right] dxdy|^2$$

where $\begin{pmatrix} u \\ v \end{pmatrix} = A \begin{pmatrix} x \\ y \end{pmatrix} + \begin{pmatrix} p_5 \\ p_6 \end{pmatrix}$, $A = \begin{pmatrix} p_1 & p_2 \\ p_3 & p_4 \end{pmatrix}$, assuming I1 and I2 are two image patches related by diffeomorphism.

Thus

$$E = 0 = | \overbrace{\iint \left[e^{\frac{-(x_\theta^2 + y_\theta^2)}{2\sigma^2}} \cos\left(\frac{2\pi}{\lambda} x_\theta\right)\right] I2\big(u(x,y), v(x,y)\big) dxdy}^{< g_i, A(\vec{p}) \circ I_2 >} - \cdots |^2$$

$$= | \underbrace{\iint \left[e^{\frac{-(x_\theta^2 + y_\theta^2)}{2\sigma^2}} \cos\left(\frac{2\pi}{\lambda} x_\theta\right)\right] J_{\frac{(x,y)}{(u,v)}} I2(u, v) dudv}_{< A^*(\vec{p}) \circ g_i,\ I_2 >} - \cdots |^2, \underbrace{J_{\frac{(x,y)}{(u,v)}}}_{\text{Jacobian}} = \det(A^{-1}) = \frac{1}{p_1 p_4 - p_2 p_3}$$

Note that prior to projection of one-sided neighborhoods onto Gabor receptive fields, image patches were shifted to the centroid of the to-be-projected image patch (e.g., image patches shown in **Fig. 4C** were shifted to produce the centered image patches shown in **Fig. 5B**). This ensured that a sufficient non-zero region was available for diffeomorphism detection.

For each neighborhood, we computed the affine transform with starting $\vec{p}$ = (1 0 0 1 x_shift y_shift), where x_shift and y_shift ran from -20 to 20 pixels in steps of 5 pixels; this ensured convergence for our dataset. We also computed the value of $E = \sum_i E_i$ at the end of the 10-iteration Newton process for each neighborhood. We then choose the $\vec{p}$ with minimal E.

This algorithm assumes that illumination is locally (but not globally) constant (i.e., ambient) and furthermore that surfaces are opaque. Handling transparency and specularity would require further investigation (possibly, effects due to transparency and specularity could initially be treated as noise in a first-pass estimate of surface parameters, and subsequently incorporated in a refined model).

Supplementary material:
A topological solution to object segmentation and tracking

Thomas Tsao and Doris Y. Tsao

Table of Contents

1. Introduction 2

1.1. Seeing objects in 3D space 2

1.2. Visual information for invariant surface perception 2

2. The representation of invariant surfaces in perspective transitions 3

2.1. A mathematical model of the visual environment 4

2.2. Representing 3D space through perspective transitions: 3D Embedding Theorem 8

2.3. Surface representations in transition spaces 13

2.3a. Coding the topology of 3D surfaces: Surfaciness Theorem 13

2.3b. Surfaces are invariants of perspective transformations: Fixed Vertex Theorem 14

2.3c. Rigid shape from perspective mappings 20

2.4. Persistent surfaces under changing visibility 24

2.4a. Occluding contours separate visible surfaces: One Side Projection Theorem, Local Stability Criteria of the Regular Value Set Theorem, One Owner Theorem, Separated Surface Continuation Theorem 24

2.4b. Ad hoc surface representation and the extraction of occluding contours: Border of Accretion Criteria for Occluding Contours Theorem 31

2.4c. Specifying a persistent global surface: Global Surface from the Union Surface of a Complete Sample Theorem, Fixed Owner Theorem 36

3. Extracting transition vectors from perspective transitions 38

4. Extracting perspective mappings from perspective transitions 40

5. Summary 43

References 44

"My explanation of vision was then based on the retinal image, whereas it is now based on what I call the ambient optic array."

-J.J. Gibson

1. Introduction

A fundamental quest in vision research is to understand how we perceive the invariant environment from the rapidly changing stimulus sensed during locomotion. What data in the changing visual stimulus specifies the persistent surfaces of invariant 3D objects? In this Supplementary Material, we present the full mathematical theory of ecological optics. We demonstrate that the topological and geometrical structure of the environment is specified by certain structures of light accessible to a moving observer. For the sake of clarity, we have organized this exposition to be self-contained, including additional background context as well as discussion of implications. This necessitates some redundancy with the main paper.

1.1. Seeing objects in 3D space

The eyes of terrestrial animals have evolved to see their environment: the dangers and resources in their surroundings, and the paths and obstructions that require proper actions to avoid the dangers and access the resources. Environmental objects are first of all seen as things outside of the viewer that occupy a part of space that the animal could otherwise move into. The spatial presence of objects in visual awareness sets a general condition for and prior to the rest of visual experience: shape, color, texture, distance, movement, etc.

The problem of how we see things in space was a subject of philosophical debate between empiricism and innatism. For example, Helmholtz asserted that the way the human vision system perceives distances to objects in space from motion parallax is by unconscious inference based upon past experiences (1). In Kant's philosophical statement, the form of "outer intuition" is an *a priori* given condition for any visual experience; space as the form for object representation is innate in the human mind. Despite their differences, both agreed upon one thing: the sense of objects and their surfaces in space cannot be only based upon the images of objects projected to the eyes. The 3D representation of objects and their surfaces needs additional support, either from *past experiences* or from an *a priori* construction of the human mind.

Both theories make an implicit assumption: visual information is conveyed through the retinal images in the form of local color, intensity, texture, contours, etc. In this article, we demonstrate that light rays converging on points of observation in the environment have much richer structures than those commonly associated with retinal images, and a vision system is capable of picking up these structures and extracting invariants from them to generate a 3D surface representation of the environment.

1.2. Visual information for invariant surface perception

Instead of appealing to past experiences or *a priori* mindsets, Gibson offered a different answer to the puzzle of how the brain sees invariant surfaces in space (2). He expanded the origin of visual information from the narrowly focused *retinal images* to a much richer source: the structure of ambient lights, the *optic arrays*, and asserted that the ambient lights surrounding the viewer fully specify the 3D structure of environment surfaces.

The conceptual expansion from vision based upon retinal images (more precisely, structures commonly associated with retinal images such as local color, intensity, texture, contours, etc.) to vision based upon ambient optic arrays leads to a surface-based vision theory. To understand how the ambient lights may carry more information than that already included in the form of retinal images, we need to look closely at the overall structure of ambient lights. In explaining the origin of the notion of 3D space in vision, Poincarè

introduced a new concept into vision: *perspective transition* during eye movement, and pointed out that the perception of geometrical space of three dimensions is derived from the isomorphism between composition laws of perspective transitions and that of movements of invariable objects.

> "The images of external objects are painted on the retina, which is a plane of two dimensions; these are perspectives. But as eye and objects are movable, we see in succession different perspectives of the same body taken from different points of view. We find at the same time that the transition from one perspective to another is often accompanied by muscular sensations. … Then when we study the laws according to which these operations are accompanied, we see that they form a group, which has the same structure as that of the invariable bodies. Now we have seen it is from the properties of this group that we derive the idea of geometrical space and that of three dimensions. We thus understand how these perspectives gave rise to the conception of three dimensions, although each perspective is of only two dimensions, - *because they succeed each other according to certain laws.*" (pages 68-69, (3)).

Poincare described two layers of structure in ambient lights. One is recorded in the retinal images, where ambient lights are structured according to their colors and intensities into a two dimensional sheet. Poincare called this layer of structure "perspectives". The other layer of structure is recorded in the perspective transitions, or changes between perspectives. As we will show, these changes include perspective transformations of correspondent areas and accretion/deletion of non-correspondent areas. This *inter-perspective* structure of ambient lights is relatively independent of the perspective structures involved in the transition: e.g., the same inter-perspective structure can apply to two random dot images or two pictures of objects. Poincare pointed out that the source of visual information for perceiving things in 3D space is the inter-perspective structure of ambient lights.

In Section 2, we introduce the concept of perspective transition groupoid to model the inter-perspective structure of ambient lights, and show that this groupoid completely specifies 3D surfaces. Then, in Sections 3 and 4, we describe key elements of a computational approach for picking up this inter-perspective structure. Finally, we summarize the results and discuss some interesting, important, and surprising conclusions with less restriction.

2. The representation of invariant surfaces in perspective transitions

In this section, we prove that perspective transitions fully specify 3D surfaces in the environment. **Fig. SM1** show two sets of light rays cast by the visual environment through two different focal points. A perspective transition is simply an ordered pair of two sets of light rays.

In Section 2, we define a mathematical model of the environment. Then, we first show, through the *3D embedding theorem,* that 3D space is homeomorphically mapped through biprojection to a 3D submanifold within the product of a pair of 2D ray spaces. To understand this better, think of 3D space as filled by points like points A and B in **Fig. SM1**, and consider projection through two observation foci. Each of these points maps to a pair of rays, and these ray pairs form a subset of the 4D space of ray pairs. The 3D embedding theorem shows that the set of points in the 3D world and the subset of ray pairs under biprojection have the same topological structure. In other words, biprojection preserves the topological structure of the 3D world within the pair of target ray spaces. This is important because it means the animal, through its two eyes or the movement of one eye, can directly access the topological structure of the 3D world.

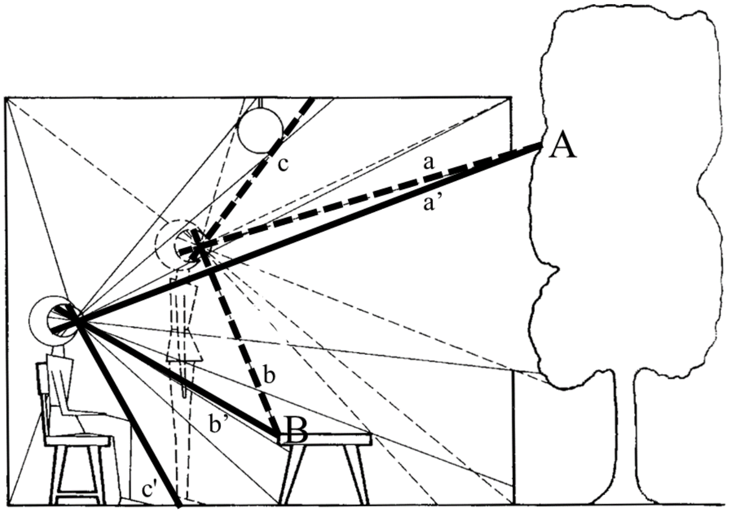

Figure SM1. The visual environment casts two sets of light rays through two different focal points ("biprojection"). At each focal point, the light rays span a 2D manifold, called the ray space. The pairs of rays {a,a'}, {b,b'}, and {c,c'} are each elements within the 4D space of pairs of rays. The pairs {a,a'} and {b,b'} are both images of points in 3D space under biprojection. The pair {c,c'} is not.

The visual environment is not a dense set of points in 3D space but is composed of a set of 2D surfaces. The 3D embedding theorem already implies that 2D surfaces in the world are topologically mapped into the product of a pair of rays spaces. However, it is not sufficient for an animal to simply know that the surfaces in the 3D world are mapped *somehow* to a submanifold within a pair of ray spaces. The animal needs to perceive the actual surface, namely the surface with specifics of its embedding in the 3D world. We next show, through the *surfaciness theorem*, that the topology of surfaces is coded by the perspective transformations between pairs of ray spaces. The surfaciness theorem shows that given a pair of ray spaces, a mapping from a domain in one ray space to another ray space fully specifies a surface in the environment. Given multiple pairs of ray spaces, the mappings provide multiple surface representations. We derive through the *fixed vertex theorem* that these different representations form an equivalence class that codes an invariant surface. Next, through the *shape from perspective mapping* theorem, we show that the surface geometry (not just topology) is also coded by the perspective mappings between ray spaces.

Surfaces in the world are always only partially visible from any single perspective due to self-occlusion and mutual occlusion (e.g., the front always occludes the back). How can an animal, which accesses only partial sensory samples of a surface at a time, perceive the same global surface all the time during its locomotion? In the last part of Section 2, we derive the mathematical conditions under which partial samples can be associated to a single, invariant surface. In Section 3, we show how the transition vectors, namely the movements of the eye, are mathematically related to the perspective transitions.

2.1. A mathematical model of the visual environment

In this subsection, we first present a model of a 3D environment composed of rigid surfaces. We define the concept of natural perspective, which models how light rays are structured by the 3D environment. Then we describe a model of locomotion around the environment. Finally, we define the concepts of perspective transition and the groupoid of perspective transitions. This will allow us, in subsection 2.2, to demonstrate how 3D space can be represented in terms of perspective transitions.

Our model of the visual world is a 3D Euclidean space partially occupied by compact objects and the ground. The objects constitute a countable set. The objects and the ground are bounded by smooth, compact, and orientable 2D manifolds, i.e., surfaces. The rationale for the surface smoothness assumption is that rough

surfaces can be approximated by smooth ones, and with smoothness we can apply techniques of mathematical analysis.

We also assume a surface called the *sky,* a sphere composed of "ideal points" which have infinite distance to any point in the Euclidean space. All objects in the environment including the ground are inside this sphere. The object surfaces and the ground surface in the Euclidean space are called *ordinary surfaces*. Together with the sky they constitute a *visual environment*.

Given a coordinate system, the Euclidean space is a topological space with the metric topology from the coordinates of its points. An ordinary surface is also a topological space with the relative topology of its ambient Euclidean space. The sky has the topology of the unit sphere at the origin of the coordinate system. The visual environment is the topological sum of the sky and the ordinary surfaces. A *connected open set* in a topological space is called a *domain*. The domain outside of the objects and the ground under the sky is called the *observation space*. We assume each ordinary surface is a connected component, which means there is no contact between different ordinary surfaces. It is a simplification of the real world.

The "connected components" are the basic units of the visual environment. The definitions of connected set (4) and components (5) are quoted as follows:

Definition (Connected Set) A topological space X is said to be *connected* if it is not the union of two non-void disjoint closed sets. A set U in a topological space is *connected* if it is not the union of two nonvoid disjoint sets, both open in the relative topology of U.

Definition (Component) Let X be a topological space and let $x \in X$. The *component* of a point x in X is $C(x)$, the union of all connected sets containing x. It is a connected closed set. If $A \subset X$, the *components* of A are the components of the points of the subspace A.

The geometric concept of *ray* plays a special role in our mathematical description of vision. A ray is a half line: an ordered set of points with a minimum element called the starting point of the ray and no maximum element. A ray r is said to pass a point P if P is a non-starting point on r. We call a ray with an assigned brightness a light ray. We assume that in the environment, only those rays with starting point on some environment surface and directed towards the air (rather than the surface interior) are light rays. We call the environment illuminated if every observation point has light rays passing in every direction.

The points and other mathematical objects, such as surfaces in the environment, are described by coordinates in a 3D Euclidean space: $E^3(O) = (\boldsymbol{i}, \boldsymbol{j}, \boldsymbol{k}; O)$. At a given observation point P, from a given direction, an infinite number of different rays could pass the observation point, varying in their starting point. For convenience in describing these equivalence classes, we set a *reference frame* by translating the origin to P: $E^3(P) = (\boldsymbol{i}, \boldsymbol{j}, \boldsymbol{k}; P)$. A ray with starting point P may then be given by the coordinates of points along the ray. By using a spherical polar coordinate system:
$ray(\theta, \phi) =$
$\{(\rho, \quad \theta, \quad \phi) = ((x^2+y^2+z^2)^{1/2}, \quad \cos^{-1}(x/(x^2+y^2)^{1/2}), \quad \cos^{-1}(z/(x^2+y^2+z^2)^{1/2})) \mid \rho \geq 0\}$ the ray can also be represented simply by its two coordinate (θ, ϕ) when there should be no confusion.

The (θ, ϕ) description for a ray passing point P leaves the starting point unspecified. We call (θ, ϕ) the *direction* of a ray passing point P. For a given illuminated environment and a point of observation, along each direction there is one and only one light ray passing that point. Thus for a given illuminated environment, we can define the brightness of a direction as the brightness of the unique light ray along that direction; we call a direction with assigned brightness an *illuminated direction*.

The set $\mathcal{S}(O)$ of rays starting at a point O is called the *ray space* based at O. Point O is the base point of $\mathcal{S}(O)$. The rays in $\mathcal{S}(O)$ are one-to-one correspondent to the points on the *unit sphere* around O. We equip the set $\mathcal{S}(O)$ with the metric topology of the unit sphere, and often make no distinction between a ray in the ray space and a point on the unit sphere surrounding the base point of the ray space. The base point of a ray space is also called the center of the ray space. The set $\mathcal{A}(O)$ of illuminated directions is called the ambient optic array based at O. The ambient optic array is a ray space in which each direction has an assigned brightness.

To give coordinates to all incoming directions to a point in an observation domain, we define the mathematical structure $\mathcal{VS}(\Omega) = \mathcal{S}\times\Omega$. This is a trivial *fiber bundle*, with bundle projection map

π: $\mathcal{VS}(\Omega) \rightarrow \Omega$,

Ω is the observation domain, $S = \{(\theta, \phi) | \theta \in [0, 2\pi)$ and $\phi \in [0, \pi]\}$ is the *ray space*. $\pi^{-1}(O)$, the ray space at the point of observation, is denoted S(O). We call $\mathcal{VS}(\Omega)$ the *visual space* on the *observation domain Ω.*

Definition (Visible Domain, Stably Visible Domain) A point of the visual environment is *visible from a point* of observation if the line segment connecting these two points does not intersect any other points on an ordinary surface. A domain of the visual environment is *visible* from a point of observation if every point in the domain is visible from the point of observation. A point of the visual environment is stably visible from a point of observation if it is also visible from a neighborhood of the point of observation. A visible domain is *stably visible* from a point of observation if it is also visible from a neighborhood of the point of observation. A visible domain is *locally stably visible* if each point of the domain has a neighborhood that is stably visible.

Definition (Point Projection) Let $\mathcal{S}(O)$ be a ray space, and P a point in the Euclidean space, $P \neq O$, $r \in \mathcal{S}(O)$ and P on r, the function from point P to the ray space $\mathcal{S}(O)$ that takes r as its value is called a *point projection*. The open line segment on the *target ray* from the *source point* to the center of the ray space is called the *path* of a point projection.

Fig. SM2 shows a point P in the Euclidean space with polar coordinate (θ, ϕ, ρ) is projected into the ray space with value (θ, ϕ).

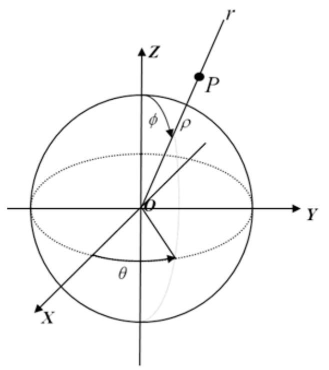

Figure SM2. Point P projects to ray space $\mathcal{S}(O)$ with value ray $r = (\theta,\varphi)$ in polar coordinate system.

Definition (Natural Perspective, Perspective Transition) A *perspective projection* from an ordinary surface in the environment to a ray space is a map from the visible part of the surface to the ray space taking

each point on the visible part of the surface as a source and taking its value under point projection to the ray space as the target. The *ambient perspective projection* to a ray space is a map from the visible part of the visual environment to the ray space through point projections. An ambient perspective projection is also called a *natural perspective*, or a *perspective*. When the environment is illuminated, the image of the natural perspective at P is a set of illuminated directions, called the *ambient optic array*. An *ambient perspective projection* to a *visual space* is a map from the visual environment to the visual space through ambient perspective projection to each of its ray spaces. An ordered pair of perspectives in a visual space is called a *perspective transition*. For brevity it is denoted a *P-transition*. Base points of the ray spaces in perspectives or in *P*-transitions are called base points of perspectives or *P*-transitions.

Some concepts need to be further clarified. (1) A perspective is a projection mapping from visible environment surfaces to a ray space. Through the source of the mapping, the visible environment surfaces, two perspectives in a perspective transition are related. (2) An ambient optic array is a set of illuminated directions at an observation point, i.e., rays with starting point at the observation point and an assigned brightness. Gibson carefully distinguished between the ambient optic array and the 2D "retinal image." One can make the set of illuminated directions look like a "retinal image" by borrowing the topology and coordinate system from the unit sphere around the point of observation (or in the case of a pinhole camera, the image plane behind the point of observation). However, the biological vision system cannot forget that the nature of an illuminated direction is a ray, not a point on the retina. The unit sphere represents a ray space, which includes an observation point hiding outside the 2D sheet, and as we shall see, this point plays a critically important function in 3D vision.

The observation space is not homogeneous for 3D movements: at each point, the allowed movements are limited by environment surfaces. An observer cannot move into the ground or inside an object. Instead of the whole 3D translation group, the set of allowed movements at each point in the observation space is a subset of the 3D translation group, different from place to place. Movements in the observation space can be represented by line segments each with specified start and end points, which we call *steps*. Unlike the situation with vectors in a vector space, the composition law of steps can only be partially defined at best: In a convex subspace of the observation space, if the end point of one step is the start point of a second step, the steps can be added together to yield a third step. If the space in not convex, even this partial composition can fail.

Our intuitive notion of locomotion in the observation space contains two concepts: (1) steps, and (2) paths, where a movement from one place to another place with a sequence of steps is called a path. Two consecutive paths can always be added together to form a longer path. The set of all paths thus formed in the observation space equipped with the partially-defined composition law we have just described has a group-like algebraic structure, including the trivial (zero) path, the inverse path of a path, and the association laws of compositions of paths. The algebraic structure of this path model of animal locomotion in its environment is a *groupoid*. The definition of groupoid was first given by Brandt ((6), also see (5, 7)).

Definition (Groupoid) A *groupoid* with base $\boldsymbol{B}$ (or "over $\boldsymbol{B}$") is a set $\boldsymbol{G}$ with mappings α and β from $\boldsymbol{G}$ onto $\boldsymbol{B}$ and a partially defined binary operation $(g,h)\mapsto gh$, satisfying the following four conditions:

1. gh is defined whenever $\beta(g)=\alpha(h)$, $\alpha(gh)=\alpha(g)$, and $\beta(gh)=\beta(h)$.

2. Associativity: If either $(gh)k$ or $g(hk)$ is defined, then both are defined and they are equivalent.

3. For every $g\in G$, there are left- and right-identity elements λ_g and ρ_g respectively, satisfying $\lambda_g g = g = g\rho_g$ (identity elements are defined for each element in $\boldsymbol{G}$).

4. Every $g\in G$ has an inverse g^{-1} satisfying $g^{-1}g=\rho_g$ and $gg^{-1}=\lambda_g$.

Definition (Path of Locomotion, Leap, Locomotion Groupoid, Motion Groupoid) Two steps are called *consecutive* if the end of the first step is the start of the second step. An ordered sequence of finite consecutive steps is called a *path*, the start of the first step is called the start of the path, the end of the last step is called the end of the path. The start point of a path, the ordered sequence of start points of intermediate steps, and the end point of a path are together called the *footprints of the path*. Two paths with common start and end points are called equivalent paths. Each equivalence class of paths is called a *leap*. The set of paths in the observation space with the groupoid algebra structure is called the *locomotion groupoid* on the observation space. The base of the locomotion groupoid is the observation space. The groupoid generated by the set of leaps is called the *motion groupoid*.

The *perspective on an observation domain* Ω defines a function on $\Omega\times\Omega$ that takes *P*-transitions as its values: $\mathcal{P}\times\mathcal{P}$: $(P,Q)\mapsto(\mathcal{P}(P), \mathcal{P}(Q))$, where $\mathcal{P}(P)$, $\mathcal{P}(Q)$ are the perspectives at *P, Q*, respectively. Let $(\mathcal{P}(P), \mathcal{P}(Q))$ and $(\mathcal{P}(Q), \mathcal{P}(R))$ be two *P*-transitions, *P, Q, R* are points in Ω; with the composition law of perspective transitions $(\mathcal{P}(P), \mathcal{P}(Q)) \bullet (\mathcal{P}(Q), \mathcal{P}(R)) = (\mathcal{P}(P), \mathcal{P}(R))$, the perspective transition groupoid is isomorphic to the motion groupoid, since they have the same base space, one-to-one correspondence of their members, and homomorphism in their compositions. Let $\tau : \overrightarrow{PQ}\mapsto (\mathcal{P}(P), \mathcal{P}(Q))$ be the correspondence of the isomorphism, we may use $\tau(P,Q)$ to denote the *P*-transition correspondent to the motion vector $\overrightarrow{PQ}$: $\tau(P,Q)=\tau(\overrightarrow{PQ})$.

Definition (Perspective Transition Groupoid) The groupoid of *P*-transitions with base points in a domain of observation space Ω and with the above given composition law of the *P*-transitions is called the *perspective transition groupoid* on Ω, denoted as $PTG(\Omega)$. For $\tau \in PTG(\Omega)$, $\alpha(\tau)\in\Omega$ is called the start position of τ, $\beta(\tau)\in\Omega$ is called the end position of τ, $\mathcal{S}(\alpha(\tau))$ is called the *start ray space* of τ, $\mathcal{S}(\beta(\tau))$ is called the *end ray space* of τ.

2.2. Representing 3D space through perspective transitions: 3D Embedding Theorem

Definition (Stereo and Conjugate Sets of a *P*-Transition) Let $(\mathcal{P}(P), \mathcal{P}(Q))$ be a *P*-transition, $\Sigma(\mathcal{P}(P))$ the set of source points of point projections in $\mathcal{P}(P)$ and $\Sigma(\mathcal{P}(Q))$ the set of source points of point projections in $\mathcal{P}(Q)$. A point projection *f* in $\mathcal{P}(P)$ or in $\mathcal{P}(Q)$ is called a *stereo point projection* if its source $\sigma(f) \in \Sigma(\mathcal{P}(P))\cap\Sigma(\mathcal{P}(Q))$. Two rays $r_p \in \mathcal{S}(P)$ and $r_q \in \mathcal{S}(Q)$ are *conjugate* to each other if they have the same source point in natural perspective; both r_p and r_q are called stereo rays. Sets of stereo rays in a ray space of a *P*-transition are called *stereo sets* of the *P*-transition. Two stereo sets are *conjugates* of each other if each ray in one set has its conjugate in the other set.

Poincare pointed out an obvious but profound fact: a perspective transition is defined on two ray spaces whose product constitutes a 4D space. This is what makes it possible for the perspective transitions to encode 3D space. These two ray spaces are able to simultaneously record the spatial locations of two rays each passing a perspective center. These joint events occupy a 4D space. A point in Euclidean space can project to two rays passing the two perspective centers. It is a joint event and can have a coordinate in the 4D transition space, just as any other joint events with two rays. But it is a special joint event: the two rays emanating from a point in space must lie in a plane, and the angle between the first and second rays is

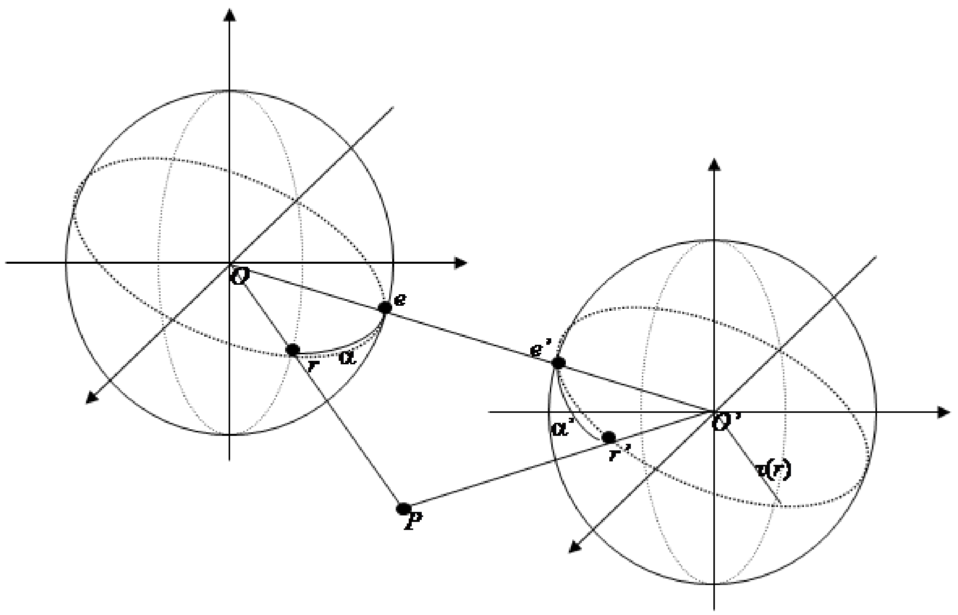

Figure SM3. The plane passing points *P, O, O'* is a *transition plane*, *e* and *e*' are two *transition poles*. The minor open arc on the transition circle linking ray *r* to the transition pole *e* is the *transition arc* α of the ray *r*. Ray *τ(r)* is the parallel transport of ray *r*. The *space-like conjugate arc* of ray *r* is the transition arc from *e*' to its parallel transport *τ(r)*.

restricted to be larger than 0 and less than π (**Fig. SM3**). A general joint event need not obey these restrictions. We will prove that these *coplanar* rays occupy a 3D submanifold of the 4D transition space, and the further restricted 3D submanifold is homeomorphic to the Euclidean space.

Before going further, we first define some important geometric objects associated with perspective transitions.

Definition (Transition Geometry of a Visual Space) Given a visual space, an element of the motion groupoid on the base space of the visual space from point *O* to point *O'*, denoted as $\overrightarrow{OO'}$, is called a *transition vector*, or simply a *transition*. The Euclidean length of a transition vector is called the *distance of the transition*. The product $\mathcal{S}(O)\times\mathcal{S}(O')$ of the two ray spaces is called the *transition space* of the transition $\overrightarrow{OO'}$. The transition space of the null transition vector is a single ray space. The transition space $\mathcal{S}(O')\times\mathcal{S}(O)$ is called the *transpose transition space* of $\mathcal{S}(O)\times\mathcal{S}(O')$. The point $(\theta_1,\phi_1,\theta_1',\phi_1')\in\mathcal{S}(O)\times\mathcal{S}(O')$ is the transpose point of $(\theta_2',\phi_2',\theta_2,\phi_2)\in\mathcal{S}(O')\times\mathcal{S}(O)$ if $(\theta_1,\phi_1)=(\theta_2,\phi_2)$ and $(\theta_1',\phi_1')=(\theta_2',\phi_2')$, and vice versa. A point in a transition space has two component rays, the source ray and the target ray. Both ray spaces are called ray spaces of the transition. Line $\overline{OO'}$ is called the *transition line*. A plane passing the transition line is called a *transition plane*. In terms of the unit sphere representation of the ray spaces, the transition geometry also includes: two *transition poles* which are the two points of the unit spheres of the two ray spaces that intersect the line segment $\overline{OO'}$, the *transition circles* which are intersections of the two ray spaces and transition planes, and the *transition arcs* which are the minor open arcs of the transition circles linking the rays to the transition poles. The transition plane, transition pole, transition circle, and transition arc of a null transition are null sets.

Definition (Biprojection) For each point *Q* in the Euclidean space other than the two points *O* and *O',* the mapping from *Q* to the transition space $\mathcal{S}(O)\times\mathcal{S}(O')$ by way of point projections from *Q* to $\mathcal{S}(O)$ and *Q* to

$\mathcal{S}(O')$ is called a *point biprojection* from Q to the 4D space $\mathcal{S}(O)\times\mathcal{S}(O')$. Let D be a domain in the 3D Euclidean space, $O\in D$ and $O'\in D$, the mapping from D to the 4D space $\mathcal{S}(O)\times\mathcal{S}(O')$ through point biprojections is called a *biprojection* from D to $\mathcal{S}(O)\times\mathcal{S}(O')$.

We show in the 3D embedding theorem that the set of points in the 3D world and the subset of ray pairs in the 4D transition space under biprojection have the same topological structure. In other words, biprojection preserves the topological structure of the 3D world in the transition space. We call the image of the 3D Euclidean space in the transition space the stereo space. Each of its elements is called a stereo pair. This is important because it means the animal, through its two eyes or a movement of one eye, can directly access the topological structure of the 3D world if the signals from the two eyes are used in a combinatorial way, e.g., as inputs to binocular cells, rather than in a complementary way, e.g., as inputs to two classes of monocular cells. Whether the two 2D retinas span a 4D space or form a union of two 2D spaces, which is still a 2D topological space, depends on how they are used.

Fig. SM3 shows the basic geometric objects in the transition space during locomotion.

Now we show that through biprojection, the topological structure of the 3D environment is represented in the transition spaces. The proper mathematical concept for describing "representation" is manifold *embedding*. The following definition is quoted from page 22 of (8).

Definition Let $\psi : M \rightarrow N$ be C^∞.

(a) ψ is an *immersion* if $d\psi_m$ is non-singular for each $m \in M$.
(b) *The pair* (M, ψ) is a *submanifold* of N if ψ is a one-to-one immersion.
(c) ψ is an *embedding* if ψ is a one-to-one immersion which is also a homeomorphism into; that is, ψ is open as a map into $\psi(M)$ with the relative topology.
(d) ψ is a *diffeomorphism* if ψ maps M onto N and ψ^{-1} is C^∞.

A diffeomorphism is a smooth homeomorphism.

Definition (Parallel and Opposite Parallel Rays) Let $\mathcal{S} = \mathcal{S}(O)$ and $\mathcal{S}' = \mathcal{S}(O')$ be the two ray spaces of a transition from O to O', $ray(\theta',\phi')\in \mathcal{S}'$ is called *parallel* to $ray(\theta, \phi)\in \mathcal{S}$ if in the coordinate system representation used in this article $(\theta',\phi') = (\theta,\phi)$, called *opposite parallel* if $(\theta',\phi') = ((\pi+\theta) \bmod 2\pi, \pi-\phi)$.

Definition (Restricted Euclidean Space) Given transition $\overrightarrow{OO'}$, the 3D manifold ($E^3\backslash\overline{OO'}$) is called the *restricted Euclidean Space* of the transition.

Definition (Stereo Mapping, Stereo Diffeomorphism, Vertex Set, Stereo Space) Give transition $\overrightarrow{OO'}$, a mapping from an open set in ray space $\mathcal{S}(O)$ to ray space $\mathcal{S}(O')$ is a *stereo mapping* if every ray in the source set intersects with its image in the target ray space. A stereo mapping is called a *stereo diffeomorphism* if it is diffeomporphic. The intersection point is called a *vertex* of the mapping. A pair of rays is called a *stereo pair* if one is the target of the other under some stereo map. The vertex of a pair of rays with the same direction is a point in the sky. A stereo mapping determines a *vertex set*. The assignment of the vertex of two stereo rays gives the *inverse mapping* of a biprojection. The inverse mapping is called 3D recovery. The set of all stereo pairs in the transition space is called the *stereo subspace* of the transition space, or simply called the *stereo space* of the transition.

Theorem (3D Embedding) Given two ray spaces centered at O and O', the biprojection map from the restricted Euclidean Space $M = (E^3 \backslash \overline{OO'})$ to the transition space $N = \mathcal{S}(O) \times \mathcal{S}(O')$ is a manifold embedding.

Proof: First we show the biprojection from M to $\mathcal{S}(O) \times \mathcal{S}(O')$ is an immersion at every point $(x, y, z) \in E^3 \backslash \overline{OO'}$. Without loss of generality, we assume the z-axis of reference frame $\mathcal{F} = (\boldsymbol{i}, \boldsymbol{j}, \boldsymbol{k}; O)$ passes point O' (**Fig. SM4**). The coordinate of O' in frame $\mathcal{F}(O) = (\boldsymbol{i}, \boldsymbol{j}, \boldsymbol{k}; O)$ is $(0, 0, l)$, $l = \left\|\overline{OO'}\right\|$. A point $Q \in M$ with $\mathcal{F}(O)$ coordinate (x, y, z) has its $\mathcal{F}(O') = (\boldsymbol{i}, \boldsymbol{j}, \boldsymbol{k}; O')$ coordinate $(x, y, z-l)$. The biprojection mapping $\psi : (x, y, z) \mapsto (\theta, \phi, \theta', \phi')$ is given by the following formula:

$$\begin{cases} \theta = \cos^{-1}\left(x \big/ \sqrt{x^2+y^2}\right) \\ \phi = \cos^{-1}\left(z \big/ \sqrt{x^2+y^2+z^2}\right) \end{cases} \text{ and } \begin{cases} \theta' = \cos^{-1}\left(x \big/ \sqrt{x^2+y^2}\right) \\ \phi' = \cos^{-1}\left((z-l) \big/ \sqrt{x^2+y^2+(z-l)^2}\right) \end{cases}$$

We show at point Q, $d\psi_Q$ has rank 3. Now

$$d\psi_Q = \begin{bmatrix} \frac{-|y|}{x^2+y^2} & \frac{zx}{(x^2+y^2+z^2)\sqrt{x^2+y^2}} & \frac{-|y|}{x^2+y^2} & \frac{(z+l)x}{(x^2+y^2+(z+l)^2)\sqrt{x^2+y^2}} \\ \frac{x(sign(y))}{x^2+y^2} & \frac{zy}{(x^2+y^2+z^2)\sqrt{x^2+y^2}} & \frac{x(sign(y))}{x^2+y^2} & \frac{(z+l)y}{(x^2+y^2+(z+l)^2)\sqrt{x^2+y^2}} \\ 0 & \frac{-\sqrt{x^2+y^2}}{(x^2+y^2+z^2)\sqrt{x^2+y^2}} & 0 & \frac{-\sqrt{x^2+y^2}}{(x^2+y^2+(z+l)^2)\sqrt{x^2+y^2}} \end{bmatrix}$$

We first subtract the 3rd column by the 1st column. Since $x^2+y^2>0$, either $x \neq 0$ *or* $y \neq 0$. If $y \neq 0$, we multiply the second row by $-x/y$ and add to the first row, so that we have the matrix

$$\begin{bmatrix} \frac{-sign(y)(x^2+y^2)}{(x^2+y^2)y} & 0 & 0 & 0 \\ \frac{x(sign(y))}{x^2+y^2} & \frac{zy}{(x^2+y^2+z^2)\sqrt{x^2+y^2}} & 0 & \frac{(z+l)y}{(x^2+y^2+(z+l)^2)\sqrt{x^2+y^2}} \\ 0 & \frac{-\sqrt{x^2+y^2}}{(x^2+y^2+z^2)\sqrt{x^2+y^2}} & 0 & \frac{-\sqrt{x^2+y^2}}{(x^2+y^2+(z+l)^2)\sqrt{x^2+y^2}} \end{bmatrix}$$

Now

$$\begin{vmatrix} \frac{zy}{(x^2+y^2+z^2)\sqrt{x^2+y^2}} & \frac{(z+l)y}{(x^2+y^2+(z+l)^2)\sqrt{x^2+y^2}} \\ \frac{-\sqrt{x^2+y^2}}{(x^2+y^2+z^2)\sqrt{x^2+y^2}} & \frac{-\sqrt{x^2+y^2}}{(x^2+y^2+(z+l)^2)\sqrt{x^2+y^2}} \end{vmatrix}$$

$$= \frac{ly}{(x^2+y^2+z^2)(x^2+y^2+(z+l)^2)\sqrt{x^2+y^2}} \neq 0.$$

If $x \neq 0$, we multiply the first row by $-y/x$ and add to the second row, so that we have the matrix

$$\begin{bmatrix} \frac{-|y|}{x^2+y^2} & \frac{zx}{(x^2+y^2+z^2)\sqrt{x^2+y^2}} & 0 & \frac{(z+l)x}{(x^2+y^2+(z+l)^2)\sqrt{x^2+y^2}} \\ \frac{(x^2+y^2)(sign(y))}{x(x^2+y^2)} & 0 & 0 & 0 \\ 0 & \frac{-\sqrt{x^2+y^2}}{(x^2+y^2+z^2)\sqrt{x^2+y^2}} & 0 & \frac{-\sqrt{x^2+y^2}}{(x^2+y^2+(z+l)^2)\sqrt{x^2+y^2}} \end{bmatrix}$$

Now $\begin{vmatrix} \frac{zx}{(x^2+y^2+z^2)\sqrt{x^2+y^2}} & \frac{(z+l)x}{(x^2+y^2+(z+l)^2)\sqrt{x^2+y^2}} \\ \frac{-\sqrt{x^2+y^2}}{(x^2+y^2+z^2)\sqrt{x^2+y^2}} & \frac{-\sqrt{x^2+y^2}}{(x^2+y^2+(z+l)^2)\sqrt{x^2+y^2}} \end{vmatrix}$

$$= \frac{lx}{(x^2+y^2+z^2)(x^2+y^2+(z+l)^2)\sqrt{x^2+y^2}} \neq 0.$$

In either case, $d\psi_Q$ has rank 3. Therefore point (x, y, z) is a regular point. If two regular points have the same image in N under biprojection, they must be the same point. This is because if two rays intersect, that have only one intersection point. Thus biprojection is injective. Now we show the inverse mapping of biprojection from $N=\psi(M)$ to M, $\psi^{-1}: N \to M$, is continuous when N is given the relative topology of the transition space. Using the same coordinate system as before, let $P_0 = (x_0, y_0, z_0) \in E^3 \backslash \overline{OO'}$ be a point of the restricted Euclidean space, and the point $(\theta_0, \phi_0, \theta_0', \phi_0') \in \mathcal{S}(O) \times \mathcal{S}(O')$ its image in the transition space under biprojection. In this coordinate representation, we have $\theta_0 = \theta_0'$, and $0 < \phi_0 < \phi_0' < \pi$. In terms of transition space coordinates, the vertex point has the following Euclidean space coordinates:

$$\begin{cases} x = \frac{l \sin\phi' \sin\phi \cos\theta}{\sin(\phi' - \phi)} \\ y = \frac{l \sin\phi' \sin\phi \sin\theta}{\sin(\phi' - \phi)} \\ z = \frac{l \sin\phi' \cos\phi}{\sin(\phi' - \phi)} \end{cases},$$

for all transition space points $(\theta, \phi, \theta', \phi') \in \mathcal{S}(O) \times \mathcal{S}(O')$, where $\theta = \theta'$, $0 < \phi < \phi' < \pi$. This is a continuous function from the image points under biprojection in the transition space to the preimage points of the restricted Euclidean space. Therefore the biprojection mapping from M to N is a homeomorphism and ψ is an embedding. *QED.*

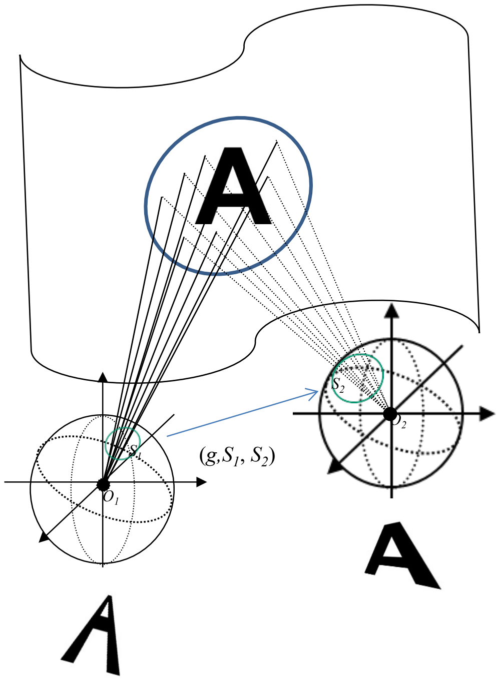

Figure SM4. The surface with label A is mapped into the stereo space of the transition $\overrightarrow{O_1O_2}$ as the image of a diffeomorphism (g,S_1 ,S_2), which is a 2-D manifold in the transition space $\mathcal{S}(O_1)\times \mathcal{S}(O_2)$. There are infinite number of such copies of the surface with label A in the visual space.

<u>2.3. Surface representations in transition spaces</u>

<u>2.3a. Coding the topology of 3D surfaces: Surfaciness Theorem</u>

How can we see surfaces from ambient lights? First of all, we need to clarify the meaning of seeing surfaces. A surface is not just a *point set*. It is a topological structure. Gibson created the term *surfaciness* to indicate something with *spatial presence* and with *continuous extension* like a sheet. Many vision researchers assume the topological quality of being a surface is imposed by the human mind (a 3D surface *"interpretation"* of the retinal image) based upon some discrete depth maps obtained from feature point correspondences between binocular (or motion) stereo pairs. In Gibson's view, surfaciness is *directly seen* from the ambient lights and is not the result of a subsequent process of data interpolation. He suggested the information for surfaciness can be found in the *transformations* between optic arrays: "the available information in the optic array for *continuity* could be described as the *preservation of adjacent order*."

We have shown that biprojection from the 3D Euclidean space to the 4D transition space is a topological embedding. Surfaces are 2D submanifolds in 3D Euclidean space. The surface representation problem then is a part of the 3D embedding problem: during 3D embedding, a 2D submanifold of the 3D Euclidean space is also embedded in the 4D transition space, in the 3D stereo subspace. The image points in the transition space resulting from the 3D embedding are constrained by stereo diffeomorphism. The points in the transition space from the 3D embedding of a surface are characterized by an additional constraint: each ray has a *unique* conjugate ray, and the correspondence mapping between conjugate rays is a homeomorphism; we prove this in the *stereo mapping theorem*. The sets in the transition space constrained by a stereo map specify the surface; we prove this in the *surfaciness theorem*.

Theorem (Surfaciness) Let O and O' be two points in observation space Ω, g a diffeomorphism: g: $S_o \to S_{o'}$ from a domain in the ray space $S(O)$ to a domain in the ray space $\mathcal{S}(O')$: $S_o \subset \mathcal{S}(O)$, $S_{o'} \subset \mathcal{S}(O')$. The set $M = \{(\theta,\phi,\theta',\phi') \mid (\theta,\phi) \in S_o,\ (\theta',\phi') \in S_{o'}, \text{ and } (\theta',\phi') = g(\theta,\phi)\}$ is a 2D manifold in the transition space. If g is a stereo mapping and M is a stereo set of $\mathcal{S}(O) \times \mathcal{S}(O')$, the biprojection ψ from space $E^3 \backslash \overline{OO'}$ to the transition space of the transition $\overrightarrow{OO'}$ has inverse on M, and $\psi^{-1}(M)$ is a surface.

Proof: A *diffeomorphism* : g: $S_o \to S_{o'}$ can be written as follows:

$$\begin{cases} \theta' = & g_1(\theta,\phi) \\ \phi' = & g_2(\theta,\phi) \end{cases}, \textit{ where } (\theta,\phi) \in S_o,\ (\theta',\phi') \in S_{o'}.$$

Let $\begin{cases} F_1(\theta,\phi,\theta',\phi') = \theta' - g_1(\theta,\phi) \\ F_2(\theta,\phi,\theta',\phi') = \phi' - g_2(\theta,\phi) \end{cases}$, $F(\theta,\phi,\theta',\phi') = \begin{pmatrix} F_1(\theta,\phi,\theta',\phi') \\ F_2(\theta,\phi,\theta',\phi') \end{pmatrix}$ is a mapping from the 4D submanifold $N = S_o \times S_{o'}$ of the transition space into a 2D Euclidean space R^2.

$DF = \begin{pmatrix} -\partial g_1/\partial\theta & -\partial g_1/\partial\phi & 1 & 0 \\ -\partial g_2/\partial\theta & -\partial g_2/\partial\phi & 0 & 1 \end{pmatrix}$ has rank of 2. From the preimage theorem, $F = \begin{pmatrix} 0 \\ 0 \end{pmatrix}$ cuts a 2D submanifold $M = \{(\theta,\phi,\theta',\phi') \mid (\theta,\phi) \in S_o,\ (\theta',\phi') \in S_{o'}, \text{ and } (\theta',\phi') = g(\theta,\phi)\}$ in the 4D manifold $N = S_o \times S_{o'}$. When g is a stereo diffeomorphism, M is a subset in the 3D stereo space. The vertex set from 3D recovery is homeomorpic to M, and therefore a 2D manifold in the Euclidean space, a surface. *QED*.

The theorem shows the environment surfaces are locally specified by the diffeomorphisms between the ray spaces in a perspective transition. In the proof of the surfaciness theorem, we have shown that a *triple* (g, $S1$, $S2$) specifies *a 2D submanifold* of the 4D manifold $S1 \times S2$. Thus a surface is not specified by a set of points. *A surface is specified by a function, a stereo diffeomorphism which maps each source ray to its stereo conjugate.*

Note that in real life, the coplanar diffeomorphism g need not be specified exactly. A visual representation is always an approximation, and an approximation to g can be given by a finite order Taylor approximation. This method of surface approximation is very different from the conventional approach to approximate surfaces by *depth maps*. The triple representation directly specifies the topological quality of being a 2D manifold (*surfaciness*) as well as the geometric shape and location of the surface. These three pieces of information are what the vision system needs for surface representation. Neither shape nor topology can be conveyed by a depth map: a set of discrete 3D points possesses no 2D manifold topological structure nor any shape.

2.3b. Surfaces are invariants of perspective transformations: Fixed Vertex Theorem

We have just shown that in a given P-transition, a stereo diffeomorphism between domains in two ray spaces provides a code for a surface in the Euclidean space. Now we further demonstrate that any environment surface can be locally represented by stereo diffeomorphisms. Furthermore, for a local surface, the set of all the different diffeomorphisms of a local surface as taken from different P-transitions form an equivalence class and represent the same local surface.

We now define a topology for the visual space with which the mappings representing the surface are defined. The simplest way to make such a topological space is to take the topological sum of all the ray spaces.

Definition (Ray Space Topology of Visual Space) Let $\mathcal{VS}(\Omega) = \mathcal{S} \times \Omega$ be the visual space over observation

domain Ω, and $\mathcal{T}(P)$ the topology (family of all open sets) of ray space $\mathcal{S}(P)$. $\mathcal{B}(\Omega) = \bigcup_{P\in\Omega} \mathcal{T}(P)$ is called the *bundle of the ray space topologies* $\mathcal{S}(P)$. For $N \in \bigcup_{P\in\Omega} \mathcal{T}(P)$ the point $P \in \Omega$ such that $N \in \mathcal{T}(P)$ is called the *base point* of N, and denoted as $\pi_\Omega(N)$. The topological sum $\mathcal{T}(\Omega) = \coprod_{P\in\Omega} \mathcal{T}(P)$ is called the *ray space topology of the visual space* $\mathcal{VS}(\Omega)$; it is larger than $\mathcal{B}(\Omega)$, including the union of neighborhoods in different ray spaces.

Definition (Mapping Triples, Subtriples of a Mapping Triple) Let S_1 and S_2 be two open sets in the ray space topology of $\mathcal{VS}(\Omega)$, g: $S_1 \rightarrow S_2$ a diffeomorphism from S_1 to S_2, the 2D submanifold of $S_1 \times S_2$ constrained by mapping g according to the surfaciness theorem is called a *mapping triple* and denoted by $\mu\tau(g, S_1, S_2)$. It is also called a *triple*. A mapping triple $\mu\tau(h_{PQ}, T_1, T_2)$ is called a *subtriple* of the mapping triple $\mu\tau(g_{PQ}, S_1, S_2)$ if (h_{PQ}, T_1, T_2) is a *restriction* of (g_{PQ}, S_1, S_2), i.e., $T_1 \subseteq S_1$, $T_2 \subseteq S_2$ and $h_{PQ} = g_{PQ}|_{T_1}$.

Can an animal's vision system respond selectively only to the triples from the stereo space? If so, then the vision system will be selective for surfaces in space. The brain with such a neural wiring would have a Kantian mind possessing the *a priori* form of seeing surfaces in space.

While the surfaciness theorem shows that stereo diffeomorphisms determine surfaces, it is possible that for a local surface there may not be a stereo diffeomorphism in the visual space serving as its representation. The next theorem shows that surfaces do actually generate stereo diffeomorphisms; therefore all visible surfaces are coded in the form of triples. A basic mathematical concept used here is the *regular mappings* (diffeomorphisms) between different ray spaces.

Theorem (Existence of Stereo Diffeomorphism) If a domain of the visual environment is stably visible from the two base points of a transition, then there is a stereo diffeomorphism between the ray spaces with the visible domain as its vertex set.

Proof: Let $\mathcal{VA}(P_1,P_2)$ be a domain of the visual environment, f_1: $\mathcal{VA}(P_1,P_2) \rightarrow \mathcal{S}(P_1)$ and f_2: $\mathcal{VA}(P_1,P_2) \rightarrow \mathcal{S}(P_2)$ perspective projections. Suppose there is some singular point of the projections f_1 or f_2, then with arbitrarily small perturbation, the singularity will degenerate into folds possibly accompanied by cusps (Arnold 1981, Whitney's theorem on plane-to-plane mappings), and no matter how small the perturbation, the surface area must include some part not visible after the perturbation because of the existence of a fold. Thus the domain is not stably visible, contradicting our hypothesis. This means the domain must be regular at every point on $\mathcal{VA}(P_1,P_2)$. Now for each regular point p, there is a neighborhood $N(p) \subseteq \mathcal{VA}(P_1,P_2)$ on which f_1 and f_2 are diffeomorphisms to neighborhoods in the range of f_1, $\mathcal{RG}(f_1)$, and neighborhoods in the range of f_2, $\mathcal{RG}(f_2)$, respectively. Therefore the mapping from ray space $\mathcal{S}(P_1)$ to ray space $\mathcal{S}(P_2)$ given by

$$g = f_2 \circ f_1^{-1} : \mathcal{RG}(f_1) \rightarrow \mathcal{RG}(f_2)$$

is a diffeomorphism. $\mathcal{RG}(f_1)$ and $\mathcal{RG}(f_2)$ are a pair of stereo domains. *QED*.

Definition (Perspective Mapping, Perspective Surface) Given a P-transition, the stereo diffeomorphism g from a stereo domain to its stereo conjugate as described in the above theorem is called a *natural perspective mapping* or *perspective transformation*. The stably visible surface area is called the *perspective surface* of the perspective mapping.

Actually, from the surfaciness theorem, all the stereo diffeomorphisms are perspective mappings associated with the perspective surface they determined. Later on we do not distinguish the concepts of *perspective mapping* and *stereo diffeomorphism*. The counterpart of the concept of perspective mapping in classic projective geometry is also called *perspective transformation*. They are related but different concepts. In **Fig. SM5a**, correspondences of lines in different pencils in a *projective space* are made by joint incidence to points on a plane called the perspective plane; in **Fig. SM5b**, correspondences of rays in ray spaces based at different points of observation are made by joint incidence to points on a non-flat surface, the perspective surface. In classic projective geometry, a perspective plane can be determined by three pairs of correspondent lines with intersection points not on one line. Determining a perspective surface requires a *stereo diffeomorphism*.

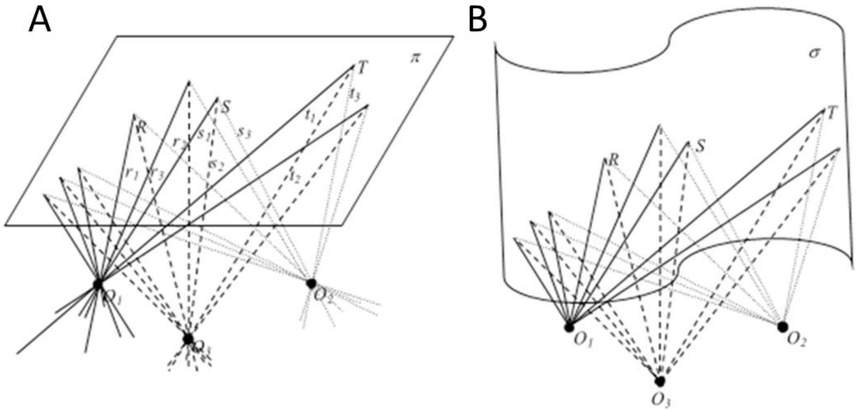

Figure SM5. (**A**) A classic perspective transformation between a pair of pencils of lines given by a perspective plane π maps lines r_1, s_1, t_1 of the line pencil at O_1 to lines r_2, s_2, t_2 of the line pencil at O_2 through incidence to points *R, S, T* on the perspective plane *π*. The plane determines a perspective transformation between any pair of line pencils in the 3D projective space, such as at points O_1 and O_2. Three pairs of correspondent lines with their intersection points not on one line determine the perspective plane, thereby the perspective transformation. (**B**) A natural perspective transformation between rays in different ray spaces is given by a surface through incidence to common points on the surface σ. The surface determines a perspective transformation between ray spaces based in the observation area.

Having shown that environment surfaces can be represented by perspective mappings, we now show the surface coded by a sequence of perspective mappings is fixed in the Euclidean space, i.e., a visible surface is an invariant of its representations in the form of perspective mappings. To prove this, we first introduce the concept of an algebraic structure of regular mappings in the visual space with the ray space topology. We have already used the concepts of *regular* and *critical* points. A formal definition is quoted below.

Definition 1.11 (from pg 33-34, (9)**).** Let *X* and *Y* be differentiable manifolds and $f: X \to Y$ a C^1-mapping. Then

(1) corank $(df)_P = \min(\dim X, \dim Y) - \text{rank}(df)_P$.
(2) a point $p \in X$ is a critical point of f if corank $(df)_P > 0$.
(3) the set of critical points of f is denoted by $C[f]$.
(4) a point $q \in Y$ is a critical value of f if $q \in f(C[f])$.
(5) a point $p \in X$ is a regular point of f if $p \notin C[f]$.
(6) a point $p \in X$ is a regular value of f if it is not a critical value of f. So, in particular, a point not in Image(f) is a regular value.

The perspective transformations are mappings between different open sets in the ray space topology of the visual space. With the ray space topology of visual space, we can now define the totality of *perspective transformations* for the visual space as a special groupoid called a *pseudogroup* (9, 10). The definition of a

pseudogroup is quoted here from Kobayashi and Nomizu (10):

Definition (Pseudogroup of Transformations) A *pseudogroup of transformations* on a topological space S is a set Γ of transformations satisfying the following axioms:

(1) Each $f \in \Gamma$ is a homeomorphism of an open set (called the domain of f) of S onto another open set (called the range of f) of S;
(2) If $f \in \Gamma$, then the restriction of f to an arbitrary open subset of the domain of f is in Γ;
(3) Let $U = \bigcup_i U_i$ where each U_i is an open set of S. A homeomorphism f of U of an open set of S belongs to Γ, if the restriction of f to U_i is in Γ for every I;
(4) For every open set U of S, the identity transformation of U is in Γ;
(5) If $f \in \Gamma$, then $f^1 \in \Gamma$;
(6) If $f \in \Gamma$ is a homeomorphism of U onto V and $f' \in \Gamma$ is a homeomorphism of U' onto V' and $V \cap U'$ is non-empty, then the homeomorphism $f' \circ f$ of $f^{-1}(V \cap U')$ onto $f'(V \cap U')$ is in Γ.

Definition (Global Transition Mapping Function, Transition Mappings of Visual Space) Let $\mathcal{VS}(\Omega) = \mathcal{S} \times \Omega$ be the visual space over observation domain Ω, $\tau\mu$ a function defined on $\Omega \times \Omega$ by assigning each (P,Q), $P \neq Q$, a *mapping triple* of the visual space: $\tau\mu(P,Q) = \mu\tau(g_{PQ}, S1, S2)$, where $S1, S2 \in \mathcal{B}(\Omega)$, $\pi_\Omega(S1) = P$, $\pi_\Omega(S2) = Q$, and $g_{PQ}: S1 \to S2$ a diffeomorphism, with the requirement that $\tau\mu(Q,P) = \tau\mu^{-1}(P,Q)$, the inverse mapping of $\tau\mu(P,Q)$, and $\tau\mu(P,P) = 1_{S(P)}$, the identity (or trivial) mapping for all $P \in \Omega$. This function $\tau\mu$ is called a global *transition mapping function* on the visual space. Each of its values is called a *global transition mapping*, denoted as a *GT*-mapping. A restriction of a global transition mapping is called *a transition mapping* with respect to the *GT*-mapping function of the visual space, or simply a *T-mapping*. The transition vector $\overrightarrow{PQ}$ is called the *transition* of the *T*-mapping. We can think of the global transition mapping function as a function defined on the set of transitions on the observation space.

Definition (Consecutive Transition Mappings and their Composition) Two *T*-mappings are consecutive if the set of targets of the first mapping is the set of sources of the second mapping. Given two consecutive *T*-mappings $h_{PQ}: SD \to SM$ and $h_{QR}: SM \to ST$, where SD, SM, and ST are open sets in ray spaces, $\pi_\Omega(SD) = P$, $\pi_\Omega(SM) = Q$, and $\pi_\Omega(ST) = R$, the mapping $h_{PQR} = h_{QR} \circ h_{PQ}: SD \to ST$ is called the composition of the two consecutive *T*-mappings.

Although with two consecutive mappings we can always properly define their composition from one ray space to another ray space, there is no guarantee their composition is also a *T*- mapping with respect to the same *GT*-mapping function.

Definition (Motion Consistent Global Transition Mapping Function) Let $\tau\mu$ be a *GT*- mapping function. If for any three points P, Q, R in the base space Ω of the visual space, and three transition mappings with respect to $\tau\mu$, $h_{PQ}: SD \to SM$, $h_{QR}: SM \to ST$ $h_{PR}: SD \to ST$, where h_{PQ}, h_{QR} are consecutive, the following diagram commutes, we call the global transition mapping function *motion consistent*.

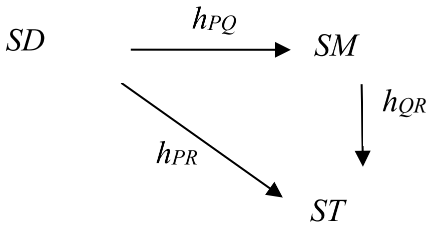

The set of *T*-mappings with respect to a *GT*-mapping function in the visual space satisfies the axioms of pseudogroup (1), (2), (4), and (5). The requirement that compositions of *T*-mappings be *motion consistent* guarantees axiom (6) is observed. The *axioms of topology* require the union of a family of open sets be included in the topology. The union of open sets across ray spaces is included in our *ray space topology*. As a consequence, maps from a source open set distributed in different ray spaces to a target open set distributed in different ray spaces are included in the mapping structure, therefore satisfying axiom 3. Such mappings are not *T*-mappings, which happen between two ray spaces. However, their existence in our pseudogroup will not cause any inconsistency with our statements about *T*-mappings.

Definition (A Mapping Structure of the Visual Space) The pseudogroup generated by the value set of a motion consistent *GT*-mapping function of the visual space with the ray space topology is called the *mapping structure* of the visual space imposed by the *GT*-mapping function, denoted by $MS(\Omega, f)$, where Ω is the observation space and f is the *GT*-mapping function. A mapping structure $MS(\Omega, f)$ is called a *stereo mapping structure* if each of its *T*-mappings is a stereo mapping.

Definition (**Perspective Mapping Structure of a Visual Space**) The mapping structure of the visual space generated by the set of natural perspective mappings on the visual space is called the *perspective mapping structure* of the visual space. The perspective mapping structure is the pseudogroup of all stereo diffeomorphisms in the visual space imposed by environment surfaces.

Now we will show the surface recovered from a stereo diffeomorphisms is invariant within the equivalence class defined by the perspective mapping structure of the visual space.

Definition (Equivalence Class with respect to a Mapping Structure) Given a mapping structure of visual space, two members of $\mathcal{B}(\Omega)$ are *equivalent* with respect to the mapping structure if there is a *T*-mapping mapping one member to the other. The equivalence classes thus formed are called *equivalence classes* with respect to the mapping structure.

Definition (Stable Open Set under a Mapping Structure, Locally Stable Mapping Structure) Let *MS* be a mapping structure in a visual space, *S* a member of $\mathcal{B}(\Omega)$. *S* is *stable* if the *base points* of members of the equivalence class of *S* with respect to the mapping structure contain a neighborhood of the base point of *S*. *S* is called *locally stable* if each ray in *S* is contained in a stable member of $\mathcal{B}(\Omega)$. A mapping structure is *locally stable*, if each of its members has a locally stable source.

Theorem (Fixed Vertex Set) Given a locally stable space-like coplanar mapping structure of a visual space, each equivalence class determined by this mapping structure determines a fixed vertex set.

Proof: Within each equivalence class, the compositions of transition mappings are motion consistent (by definition of mapping structure). Consider a ray *a* in ray space S(O_1) that has its image ray *b* in ray space S(O_2), which in turn has its image ray *c* in ray space S(O_3). Suppose O_1, O_2, and O_3 are not along one line. We can assume this due to local stability. The maps are motion consistent, therefore *a* has its image ray *c*

in ray space S(O_3). This means rays a and b are in a plane m_1, rays b and c are in a plane m_2, and rays c and a are in a plane m_3. Because the mapping structure is space-like, the intersection point p_1 of a and b exists and is on m_1, m_2, and m_3. Similarly the intersection point p_2 of b and c exists and is on m_1, m_2, and m_3, and the intersection point p_3 of a and c exists and also is on m_1, m_2, and m_3. Since p_1, p_2, p_3 are each on m_1, m_2, and m_3, we must have $p_1 = p_2 = p_3$, since three planes intersect in at most one point. Therefore the trajectory of a domain in a ray space has an invariant attached vertex set. *QED.*

We now show that the set of regular values of ambient perspective projection is locally stable under the perspective mapping structure, thus completing our demonstration that the P-mapping structure provides codes for local environment surfaces, and surfaces are invariant to the coding variations through P-transitions.

Theorem (Local Stability of the Regular Value Set) The set of regular values of a natural perspective projection is locally stable under the perspective mapping structure.

Proof: Let ray x be a regular value of a perspective projection from a surface to the ray space $\mathcal{S}(O)$, $f(.\,;O)$: $x = f(P)$, P the preimage of x on the surface. Since x is a regular value, there is a neighborhood $N(P)$ of the point P on the surface, and a neighborhood $N(x)$ in the ray space such that the map

$$f(.;O): N(P) \to N(x)$$

is a diffeomorphism. Now that $N(P)$ is a regular domain of the perspective projection to O, the determinant of Df does not vanish on $N(P)$. Let $N'(P)$ be a surface extension of $N(P)$ containing the closure of $N(P)$. We may assume $N'(P)$ is also a regular domain with compact closure, and $\det(Df(X;O)) \geq \varepsilon > 0$ for any point $X \in N'(P)$, where $\det(Df(X;O))$ is the determinant of the derivative of the perspective projection at surface point X. Referring to **Fig. SM6**, C is a cone with vertex O and base surface $\overline{N}(P)$, the closure of $N(P)$. We can cover each point of $\overline{N}(P)$ with an open set in 3D Euclidean space such that it contains no points of environment surfaces other than those of $N'(P)$, since $\overline{N}(P)$ is visible from O. and each point of the cone surface of C with an open set in the observation space. By first choosing a finite open cover Co for the cone surface C we can enlarge cone C to a cone C' within the scope of Co with same base $\overline{N}(P)$ and containing point O inside. Unlike cone C, cone C' may contain points of $N'(P)$ inside, because it is made of the open covers for the base surface $\overline{N}(P)$. Let $N(O)$ be a neighborhood of O inside the cone C'. We claim there is a neighborhood ball $N_\delta(O)$ centered at O with radius δ in the observation space, $N_\delta(O) \subseteq N(O)$, such that $N(P)$ is visible from any point in $N_\delta(O)$. If this is true, we reach the conclusion of the theorem. Suppose the contrary is true, then for any $\delta(i) = \delta/2^i$, there is some point $O_i \in N_{\delta(i)}(O)$ such that a point $P_i \in N(P)$ is occluded by some surface point $Q_i \in N'(P)$ inside the cone C'. Linking points P_i and Q_i by a path in $N'(P)$, there must be a critical point $T_i \in N'(P)$ of the perspective projection $f(.,O_i)$, and $\det(Df(T_i;O_i)) = 0$. Let $i \to \infty$, we can choose a subsequence of $\{T_i\}$ converging to a point $T_0 \in N'(P)$. The sequence of (3) converges to O. The function $\det(Df(X;Y))$ is a continuous function with respect to Y and X. Therefore $\det(Df(T_0, O)) = 0$. A contradiction shows that such a $N_\delta(O) \subseteq N(O)$ must exist. *QED.*

From Sard's theorem, which states that the image of the set of critical points of a smooth function f from one manifold to another has measure 0, it is easy to deduce that almost all points in a ray space are regular values under natural perspective projection. This local stability theorem further demonstrates for each regular value there is a stable neighborhood. Therefore, at almost every ray in a ray space, there is a stable neighborhood. The perspective mapping structure is locally stable.

The vertex set computation described in the proof of the Fixed Vertex Set Theorem is a fundamental

computation for 3D vision. It is what makes possible explicit perception of the distance of points on environment surfaces. It is only possible through explicit representation of *visual rays*, direction vectors linking an observation point behind the image plane to points on the image plane. It is the rays that reach the third dimension from the 2D retina. This is what Gibson meant when he emphasized that the eyes sense optic arrays, not retinal images; even when looking at a 2D picture, the eyes sense the optic array generated by lights starting from the 2D picture.

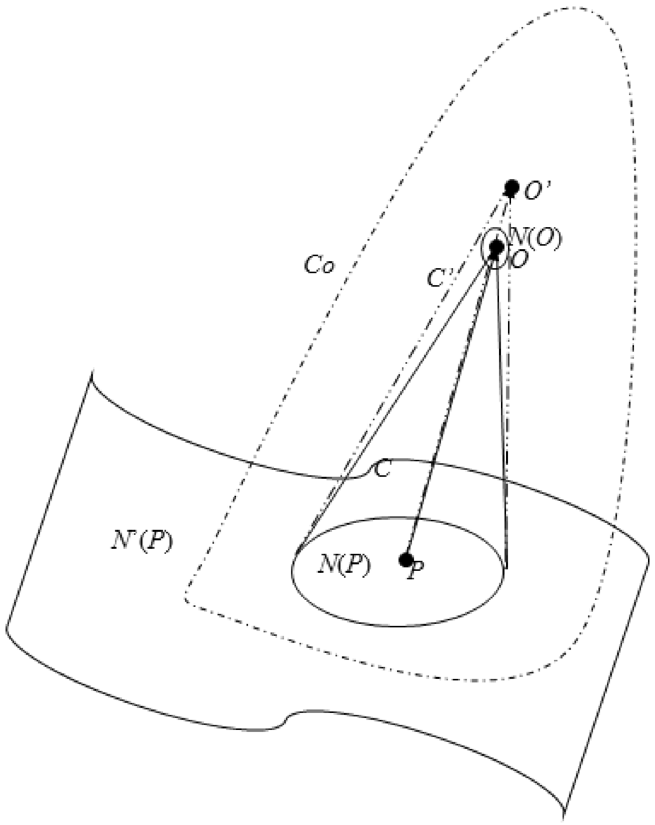

Figure SM6. The cone *C* with base $\overline{N}$ (*P*), the closure of a neighborhood of *P* on the surface *S*, as the base and the point *O* as the vertex. Since *N*(*P*) is visible from *O*, the interior of *C* contains no points of environment surfaces. Cone *C* has a finite open cover *Co* with a boundary of some minimum positive distance away from cone *C*. Cone *C' enlarges C* within the scope of *Co* containing *O* inside. *C'* may contain part of surface *N'(P)* inside.

2.3c. Rigid shape from perspective mappings

So far we have shown that the *triples* of the *perspective mapping structure* in the same equivalence class specify a surface patch fixed in 3D space, and thus provide a representation for the topology of the surface. A rigid surface also has a *geometric shape*, i.e., a specific numeric characterization of the geometric construction, the curving, of the surface. Now we further show that the geometric shape of a surface is also specified by the perspective mapping structure.

In this section, we use a simple "pinhole" camera model for representing rays in a ray space, and a translation of the observation point along the Y-axis by one unit ("binocular setting") as the coordinate representation of the *P*-transition. **Fig. SM7** represents our pinhole camera model and **Fig. SM8** represents

the binocular setting (ambient optic arrays at two observation points) with *convergent point* located at infinity.

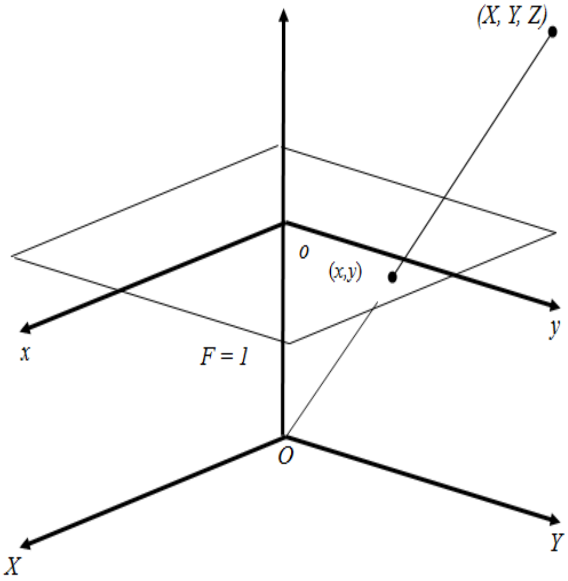

Figure SM7. An environment-associated coordinate system *O-XYZ* and an image plane coordinate system *o-xy* for a pinhole camera model are attached in a fixed geometric relation.

Using the image plane coordinate system and referring to **Fig. SM8**, a perspective mapping from domain $S_h(O)$ (the image plane for the half ray space centered at *O*) to domain $S_h(O')$ (the image plane for the half ray space centered at *O'*):

$$g : p \mapsto p' = g(p); p \in U, p' \in U'$$

can be written in terms of image coordinates: $\begin{pmatrix} x' \\ y' \end{pmatrix} = \begin{pmatrix} g_1(x,y) \\ g_2(x,y) \end{pmatrix}$,

Here $\begin{pmatrix} x \\ y \end{pmatrix}$ is the image plane coordinate representation of ray *p*, and $\begin{pmatrix} x' \\ y' \end{pmatrix}$ is the image plane coordinate representation of ray *p'*, and $\begin{pmatrix} g_1 \\ g_2 \end{pmatrix}$ are two components of the perspective mapping *g*.

The first order Taylor expansion of *g* at point p_0 with image plane coordinate (x_0, y_0) has image plane coordinate representation:

$$\begin{pmatrix} x' \\ y' \end{pmatrix} = \begin{pmatrix} g_1(x,y)) \\ g_2(x,y) \end{pmatrix} = \begin{pmatrix} g_1(x_0,y_0) \\ g_2(x_0,y_0) \end{pmatrix} + \begin{bmatrix} \dfrac{\partial x'}{\partial x} & \dfrac{\partial x'}{\partial y} \\ \dfrac{\partial y'}{\partial x} & \dfrac{\partial y'}{\partial y} \end{bmatrix} \begin{pmatrix} \Delta x \\ \Delta y \end{pmatrix} + o(\|\Delta\|^2),$$

where $\Delta = \begin{pmatrix} \Delta x \\ \Delta y \end{pmatrix} = \begin{pmatrix} x - x_0 \\ y - y_0 \end{pmatrix}$.

The translation vector $\begin{pmatrix} \delta_x \\ \delta_y \end{pmatrix} = \begin{pmatrix} g_1(x_0, y_0) - x_0 \\ g_2(x_0, y_0) - y_0 \end{pmatrix}$ is the "disparity" between corresponding centers of the two respective neighborhoods, the matrix $\begin{pmatrix} a_{11} & a_{12} \\ a_{21} & a_{22} \end{pmatrix} = \begin{bmatrix} \frac{\partial x'}{\partial x} & \frac{\partial x'}{\partial y} \\ \frac{\partial y'}{\partial x} & \frac{\partial y'}{\partial y} \end{bmatrix}_{(x_0, y_0)}$ is the derivative $dg : S_h(O)_{p_0} \to S_h(O')_{g(p_0)}$, representing a mapping from the tangent space at p_0 in area *U* to the tangent space at $g(p_0)$ in *U'*.

Using the coordinate system of **Fig. SM8**, the perspective mapping *g* has only three parameters in the first order Taylor expansion: three parameters representing the *coplanar constraint* are fixed: $\delta_x \equiv 0$, $\frac{\partial x}{\partial x'} \equiv 1$, and $\frac{\partial x'}{\partial y} \equiv 0$.

$$dg = \begin{bmatrix} a_{11} & a_{12} \\ a_{21} & a_{22} \end{bmatrix} = \begin{bmatrix} 1 & 0 \\ \frac{\partial y'}{\partial x} & \frac{\partial y'}{\partial y} \end{bmatrix}.$$

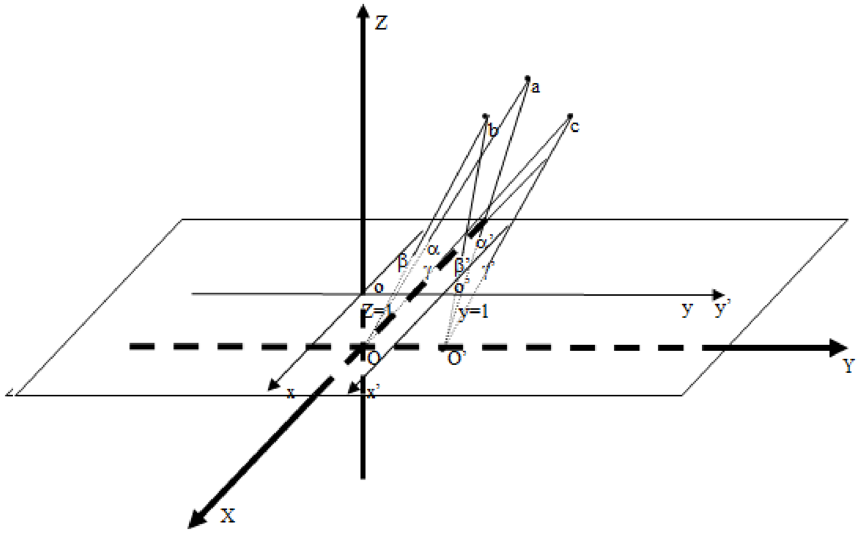

Figure SM8. Two cameras located parallel each other with one unit shift along the Y-axis. Both image planes are located on the Z=1 plane. The first center of projection is at the origin of the O-XYZ coordinate system and the second center of projection moves to O' by shifting one unit along the Y axis. The origin of the coordinate system of the second image plane o' is at y=1. Three space points *a, b, c* project to α, β, γ, and α', β', γ' in the first and second camera systems, respectively.

If $\frac{\partial y'}{\partial y} \neq 0$, point p_0 is regular and there is a neighborhood U of p_0 such that $g: U \to g(U)$ is a diffeomorphism onto the open set $g(U)$ in $S_h(O')$, Namely, there is a perspective mapping with U as its domain. The shape of the rigid surface is specified by the perspective mapping:

Theorem (Shape from Perspective Mapping) The 2D manifold in 3D space recovered from a perspective mapping g has a rigid *shape* determined by up to second order derivatives of this mapping.

Proof: From the fundamental theorem of surface in differential geometry (11) the rigid shape of a surface is determined by the *first type* and *second type* of *fundamental forms* of the surface. Now we show these fundamental forms are completely determined by the perspective mapping. First we introduce a parameter representation for the surface patch recovered from the perspective mapping *g*. With this parameterization, we then calculate its first type fundamental form and second type fundamental form, both from *g* and its derivatives. Thus we conclude that with *g*, the Euclidean geometry shape of the 2D manifold is completely specified.

The surfaces recovered from the *triples* can naturally be parameterized by the *ray space coordinates* of the domains of the triples. Using the image plane coordinate system as our ray space coordinate representation, the parameter representation of the surface patch *f* is now given by

$$f(x,y) = \begin{pmatrix} X(x,y) \\ Y(x,y) \\ Z(x,y) \end{pmatrix} = \begin{pmatrix} xZ(x,y) \\ yZ(x,y) \\ Z(x,y) \end{pmatrix}, \ (x,y) \in U .$$

With reference to the coordinate system of **Fig. SM8**, we have

$$Z(x,y) = -1/(g_2(x,y) - y)$$

and the surface *f* can be expressed in terms of *g* as follows:

$$f(x,y) = \begin{pmatrix} x/(y - g_2(x,y)) \\ y/(y - g_2(x,y)) \\ 1/(y - g_2(x,y)) \end{pmatrix}, \ (x,y) \in U .$$

From the above expression, it is easy to calculate the *first fundamental form* for the surface *f*: $df_u \bullet df_u$, in terms of $u \in U$, i.e., the parameters *x, y* taken on the image plane, *g*(*x, y*), and *dg*(*x,y*), the first derivative of *g*(*x, y*).

To compute the second fundamental form, we first look at the normal vector at a point $\begin{pmatrix} X_0 \\ Y_0 \\ Z_0 \end{pmatrix} = \begin{pmatrix} X(x_0,y_0) \\ Y(x_0,y_0) \\ Z(x_0,y_0) \end{pmatrix}$ on the surface. It is along the direction of the cross product of two tangent vectors at that point:

$$\left(\left(\partial X/\partial x,\ \partial Y/\partial x,\ \partial Z/\partial x\right)\times\left(\partial X/\partial y,\ \partial Y/\partial y,\ \partial Z/\partial y\right)\right)\Big|_{(x_0,y_0)} =$$

$$\left(Z\left(-\partial Z/\partial x,\ -\partial Y/\partial y,\ Z+x\,\partial Z/\partial x+y\,\partial Z/\partial y\right)\right)\Big|_{(x_0,y_0)}.$$

From this we have the normalized normal vector:

$$n_0=\left(a_{21}/D,\ \ (a_{22}-1)/D,\ \ (g_2(x,y)-ya_{22}-xa_{21})/D\right)\Big|_{(x_0,y_0)}.$$

where $D=\sqrt{a_{21}^2+(a_{22}-1)^2+(ya_{22}+xa_{21}-g_2(x,y))^2}$ is the normalization factor, and

$$a_{21}=\left.\frac{\partial g_2(x,y)}{\partial x}\right|_{(x_0,y_0)} \text{ and } a_{22}=\left.\frac{\partial g_2(x,y)}{\partial y}\right|_{(x_0,y_0)}.$$

We see that at each point on the surface, the normalized normal vector can be expressed by the perspective mapping *g* and its first derivative, and it is easy to calculate the *second fundamental form* for the surface *f*: $dn_u \bullet df_u$, in terms of $u \in U$, i.e., the parameters *x, y* taken on the image plane, *g*(*x,y*), *dg*(*x,y*), the first derivative and $d^2g(x,y)$, the second derivative of *g*(*x, y*). *QED*.

Thus far, we have shown that the topology and geometric shape of an environmental surface is represented by the *inter-perspective* structure imposed on the visual space by the environment: the perspective mapping structure. And we have shown that despite the variations of this code, the surface is invariant with respect to the perspective mapping structure.

2.4. Persistent surfaces under changing visibility

The visual system perceives environment surfaces invariant to changes of perspective. The term invariant surface in vision has two different meanings: when part of an environment surface is visible from different points of observation, it is seen as the same and fixed in the environment. When different parts of a surface are seen from different points of observation, they are seen as *of the same surface*.

In Section 2.3b, we showed that surfaces are invariant under variations of perspective mappings. A surface is represented by perspective mappings: these perspective mappings form an equivalence relation, and the surface is the invariant with respect to the equivalence relation of perspective mappings.

We formulate the problem of the perception of invariant surfaces under sampling variation in three steps: (1) we show that for each perspective, the visual system derives an *ad hoc* representation of surfaces with visible samples; (2) we show that an equivalence relation can be established on the different visible samples; and (3) we show that from each equivalence class of visible samples an invariant global surface can be derived.

In this section, we first introduce a general criteria for surface connectedness in terms of local surfaces and surface extensions, and define the concept of equivalence classes of visible samples of a surface in terms of surface extensions. We then describe a condition for the equivalence of visible samples obtained along a locomotion path, and demonstrate that the global surfaces are invariants along such a path.

2.4a. Occluding contours separate visible surfaces: One Side Projection Theorem, Local Stability Criteria

of the Regular Value Set Theorem, One Owner Theorem, Separated Surface Continuation Theorem

Definition (Local Surface, Surface Continuation) A connected open set of an environment surface is called a *local surface*. Let $\overline{S}$ be the closure of a local surface *S*, and $C(\overline{S}) = \{U_i \mid i \in I\}$ an open cover of $\overline{S}$, where *I* is a set of indices, U_i connected open sets of the environment surface, each with nonempty intersection with S, and $\overline{S} \subset \bigcup_{i \in I} U_i$. The set $\bigcup_{i \in I} U_i$ is called an *open continuation of the surface S*, or simply a *continuation of surface S*.

The union of two local surfaces is a connected 2D space if they have non-empty overlap ((5), 3.2.5). In general, the union of two overlapping local surfaces is not necessarily a surface: it may form a branched union (**Fig. SM9**). We call those that do result in a surface the surface extension to each local surface.

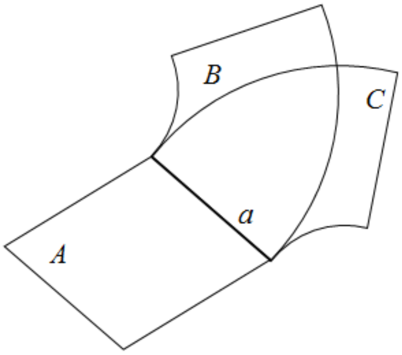

Figure SM9. Surface *A* has two separated extensions *B* and *C* from its boundary *a*. Both $A \cup B$ and $A \cup C$ are surfaces. But $A \cup B \cup C$ is not a surface. Surfaces with partial overlap form a connected 2D space but do not necessarily form a surface.

Definition (Surface Extension, Extension Chain, *E*-chain Surface, *E*-chain Connected Surfaces) The union set of two local surfaces with nonempty overlap is called a *surface extension* to each of the two local surfaces, if it is a surface. Let $\sigma = \{S_i \mid i = 1, ..., n\}$ be a sequence of local surfaces, it is called an *extension chain*, or *E-chain*, of local surfaces, if for $i= 1, ..., n-1$, $S_i \cap S_{i+1} \neq \emptyset$,, and $S_i \cup S_{i+1}$ is a surface. The surface $S = \bigcup_{i=1}^{n} S_i$ is called the *E*-chain surface of σ, and S_1 and S_n are called *E-chain connected* local surfaces, and denoted as $S_1 \sim_c S_n$. It is easy to see that E-chain connectedness is an equivalence relation of the local surfaces of the visual environment: For any local surfaces σ_i, σ_j, and σ_k, (1) $\sigma_i \sim_c \sigma_i$; (2) $(\sigma_i \sim_c \sigma_j) \rightarrow (\sigma_j \sim_c \sigma_i)$; and (3) $(\sigma_i \sim_c \sigma_j)$ & $(\sigma_j \sim_c \sigma_k) \rightarrow (\sigma_i \sim_c \sigma_k)$.

Obviously, a surface extension of a local surface is a connected surface. By induction, *E*-chain surfaces are connected surfaces. We have the statement:

Proposition The *E*-chain surface of an *E*-chain of local surfaces is a connected surface.

Taking the connected surface as the intermediate "local surface" any two simple surfaces in a connected surface are *E*-chain connected.

Theorem (*E*-chain Connected Criteria) A surface is a connected surface if and only if any of its two local surfaces are *E*-chain connected.

Proof: We only need to show the "if" part. By definition, if the surface is not connected, then it is the union of two open sets σ_1 and σ_2 of the environment surfaces with no intersection. Surfaces are locally connected. Pick from each of the two open sets a local surface. They are not *E*-chain connected. For if they are, then the *E*-chain surface connecting them, σ_e, is a connected open set. Both $\sigma_1 \cap \sigma_e$ and $\sigma_2 \cap \sigma_e$ are open sets of the visual environment. Their intersection is the null set. Therefore the set $(\sigma_1 \cap \sigma_e) \cup (\sigma_2 \cap \sigma_e) = \sigma_e$ is not a connected set. The contradiction shows if any two local surfaces of a surface are *E*-chain connected, the surface is a connected surface. *QED.*

The concepts of *E*-chain surface and *E*-chain connected surfaces are pertinent to vision for the following reason. At each point of observation, due to self-occlusion, the global surface of an object, even if it is not occluded by other objects, is only partially visible. Therefore from each perspective what the vision system can visibly access are local surfaces. On the other hand, connected surface components are units of ecological significance. They represent spatially coherent objects of the environment. It is through the *E*-chain connected surface, that the vision system is able to derive a surface component from its visible local samples.

The boundary of a 2D domain is called a *contour*. Two local surfaces are called neighboring surfaces if they are non overlapping but their boundaries have non empty intersection. The boundary between two neighboring local surfaces or two neighboring ray space domains is called a *border*.

Definition (Sandwich Domain, Regular Domain, Side of a Contour) A *sandwich domain* of a contour C in a 2D manifold is a connected open set S in the 2D manifold such that $C \subseteq S$, and $S \backslash C = S_1 \cup S_2$, S_1 and S_2 non-empty non-overlapping domains. C is called the border between S_1 and S_2. S_1 and S_2 are called *side domains* of C within S. Given a family of sandwich domains of a contour C, side domains of C of this family form equivalence classes according to the intersection relation: $S_i \sim S_j$ *iff* $S_i \cap S_j \neq \varnothing$. The equivalence class of connected side domains is called a *side class of C*. With a given perspective, a domain in the ray space is called a *regular domain* if all its points are *regular values* of the perspective. If both S_1 and S_2 are *regular domains* of the perspective, S is called a *regular sandwich domain of C*. If all members of a side class of a contour are regular domains, the side class is called a *side of the contour*.

Definition (Spatially Separated Surfaces, Contour of Surface Separation) Two local surfaces are called *spatially separated* if the intersection of their closures is a null set. A contour in a ray space is called a *contour of surface separation* if it has a regular sandwich domain such that the preimage surfaces of its two side domains are spatially separated.

A surface is a 2D manifold in the 3D Euclidean space. The perspective image of a visible surface is in the 2D ray space. Connectivity is an invariant topological property under homeomorphisms. The set of regular values in a ray space and its preimage, the set of regular points of visible surfaces, are homeomorphic under natural perspective projection. The sets of regular values in the ray space provide a local representation of sets of regular points of environment surfaces. The following theorem shows that the same holds true of surface connectivity.

Theorem (**Pre-image Surface Connectivity**) The preimage of a *connected* open set of regular values of a perspective is a connected open set of an environment surface.

Proof: Let S be a connected open set of regular values of a perspective. Consider two local surfaces of the preimage surface $f^{-1}(S)$, with images D_i and D_{i+k}. We show they are chain connected. Since S is connected, it is *E*-chain connected. For any two domains D_i and D_{i+k} in the covering there is a chain of

domains D_i, D_{i+1}, … D_{i+k} such that each consecutive pair has nonempty intersection: $D_l \cap D_{l+1} \neq \emptyset$, where $i \leq l < k$. Then for any two domains $f^1(D_i)$ and $f^1(D_{i+k})$ in the covering of the preimage surface $f^{-1}(S) = \bigcup_i f^{-1}(D_i)$, the chain of local surfaces $f^1(D_i)$, $f^1(D_{i+1})$, … $f^1(D_{i+k})$ has also nonempty intersections: $f^1(D_l) \cap f^1(D_{l+1}) \supseteq f^1(D_i \cap D_j) \neq \emptyset$, where $i \leq l < k$, for each consecutive pair. Therefore $f^{-1}(S)$ is a connected surface. *QED*.

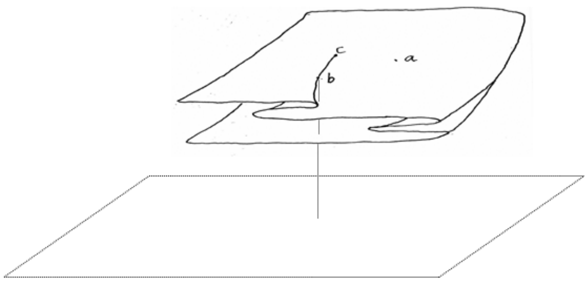

Figure SM10. In the projection from a surface to the plane below, point *b* is a fold point, *c* is a cusp point, and *a* is a regular point of this projection.

In a given perspective, the topological division of the set of regular points of the visible surfaces into connected components is represented by the components of regular values in the ray space. The visible surfaces also contain critical points and the image of a natural perspective contains critical values. The next question is: what information of the surface connectivity is carried by the set of critical values?

The structure of singularities of surface projection was described in a theorem proved by Whitney: Every singularity of a smooth mapping of a plane to a plane after an *appropriate small perturbation* splits into *folds* and *cusps* (12, 13). Knowing that surface occlusion is related to the *fold singularity* of perspective projection, the problem of surface extension across the occlusion contours can be reduced to and resolved by the topology of the surface *fold*. **Fig. SM10** shows the critical points in a projection to a plane consist of regular points, folds, and cusps.

The occluding contours are commonly described as an *intensity gradient contour* caused by *depth discontinuity* of nearby visible surfaces (14) and treated as a subset of "real contours," contours of *intensity discontinuities* in images. To define the *occluding contour* with its original meaning of *occlusion* requires distinguishing two sides of the contour. From the pre-image connectedness theorem of regular domains, we can easily deduce the following statement relating the sides of a contour in the ray space to a definite surface in the environment.

Proposition (Preimage of a Side) The preimage of a regular side domain of a contour is a local surface. In terms of connectedness, the preimage surfaces of all members of a side of the contour form an equivalence class of surfaces.

It is important to note that in the vicinity of a fold contour, two sides of the fold contour project to one side of the image contour of the fold. Only one side of the surface can be visible. The surface at the other side of the fold is occluded.

Theorem (One Side Projection) Every fold contour of an environment surface has a sandwich domain such that the images of its two side domains under point projection to the ray space are located on one side of the image of the fold contour.

Proof: Let *p* be a fold point of the environment surface. In a neighborhood of *p*, there is a parameter representation for the surface: $\begin{cases} \rho = \rho(\xi,\eta) \\ \theta = \theta(\xi,\eta) \\ \phi = \phi(\xi,\eta) \end{cases}$, and the projection to the ray space can be expressed as the mapping $f : (\xi,\eta) \mapsto (\theta(\xi,\eta), \phi(\xi,\eta))$. From the *normal form theorem* ((9), Theorem 4.5), there is a coordinate system *u* for the $\xi\eta$-plane at the fold point *p* and a coordinate system *v* for the $\theta\phi$-plane at the occluding contour point *f*(*p*) such that in this coordinate system the projection mapping has the form: $f : (u_1, u_2) \mapsto (u_1, u_2^2)$, namely $(v_1, v_2) = f(u_1, u_2) = (u_1, u_2^2)$. The sandwich domain is divided by the fold contour $u_2 = 0$ into two side domains $\{(u_1,u_2) \mid u_2 > 0\}$ and $\{(u_1,u_2) \mid u_2 < 0\}$. The image of the fold contour in the ray space is the contour $\{(v_1,v_2) \mid v_2 = 0\}$. And through the mapping *f*, which assigns $v_2 = u_2^2$, both the two side domains $\{(u_1,u_2) \mid u_2 > 0\}$ and $\{(u_1,u_2) \mid u_2 < 0\}$ are projected onto one side of the contour $\{(v_1,v_2) \mid v_2 = 0\}$, the domain $\{(v_1,v_2) \mid v_2 > 0\}$. *QED.*

The occluding contour is the image of the surface fold. The fact that the surface at the vicinity of its fold projects to one side of the occluding contour singles out this side as the "owner" of the occluding contour. The occluding contour as the image of a fold has no orientation, but we can assign an occluding contour an orientation so that the owner is on the left side. This way a border has two possible orientations, each representing a different owner side.

Definition (**Occluding Contour, *T*-junction, Owner Side**) In a given perspective, an oriented contour is called an *occluding contour* if it is the image of a fold contour of a visible surface, and the preimage of its left side is a side of the fold of the visible surface. The intersection point of two occluding contours is called a *T-junction*. The left side of the occluding contour is also called the *owner* side.

While the concept of occluding contour thus defined is directly related to the phenomenon of *occlusion* itself, it has a "drawback" compared with intensity edges and figure/ground segregations. These concepts are supported by "proximal" stimuli: the retinal image contains intensity contours and figure qualities such as symmetry and convexity. Our concept of occluding contour explicitly refers to the *environment surfaces*, which are "distal stimuli." How can the visual system directly pick up the information about occluding contours, particularly their *owners,* from the ambient lights reaching the eyes?

The points on occluding contours are critical values of perspective projection. We already know that a regular value is characterized by having some stable neighborhood. Can a critical value be characterized by some sort of unstable behavior under small perturbations of the point of observation? Indeed, as we show next, the theorem of local stability of a regular value set has an inverse: A locally stable set is a set of regular values.

Theorem (**Local Stability Criteria of the Regular Value Set**) A set is locally stable under the perspective mapping structure if and only if it is a set of regular values of natural perspective.

Proof: We only need prove the "only if" part to show all points in a locally stable open set are regular values of natural perspective projection. The set is locally stable. Therefore each point in this set has a stable

neighborhood under the perspective mapping structure. Each perspective mapping from this stable neighborhood to some other ray space defines a 2D manifold in the respective transition space which corresponds to an invariant environment surface. The stable neighborhood is a set of regular values of perspective projection from the environment surface to the ray space because the $det(Dh) = det(Dg \circ Df^{1})$, where h is a perspective mapping of the stable neighborhood between two ray spaces, and f, g are perspective projections to two ray spaces. This is true for any point in the locally stable set. The set is a set of regular values. *QED*.

This theorem says no neighborhood of an occluding contour is stable under perspective mapping. Actually, the occluding contours can be more specifically characterized in terms of accretion: Given a sandwich domain of an occluding contour, if a transition mapping maps its two side domains into another ray space, this is always accompanied by an accretion. The extraction of occluding contours from accretion between two perspectives is called da Vinci stereopsis (**Fig. SM11**).

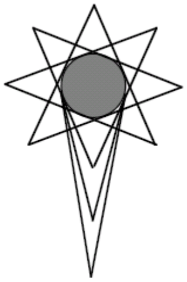

Figure SM11. Diagram from Leonardo's notebook illustrating the fact that the light rays leaving an object's surface may be considered to form a collection of cones (which Leonardo calls "pyramids"), each cone constituting an image that would be taken by a pinhole camera at a given location. The diagram shows that different cones cover different parts of an object.

The non-symmetric relation of an occluding contour to its two sides was noticed and studied in Gestalt psychology. Gestalt psychologists call the side of the surface with fold the figure side and noticed its assignment affects our perception of an image. **Fig. SM12** shows the same contour represents two different surface shapes depending on which side owns it.

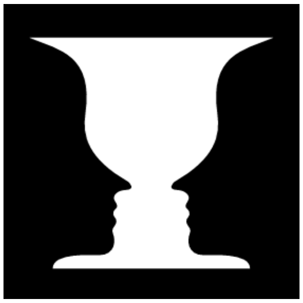

Figure SM12: Different assignments of the owner of the occluding contour affect our perception of the shape of the contour as of human face or of a vase.

We have included the *owner* assignment as part of the definition of an occluding contour. With owner assignment, the occluding contours are able to specify the surface connectivity between what is currently visible and what is occluded. As the famous Kanizsa triangle illusion shows, an occluding contour can be perveived without an accompanying intensity gradient contour, and the defining characteristic of an occluding contour is border ownership, not the intensity gradient.

The one-sided projection justifies the "owner" concept for the occluding contour. There would be a problem if the same contour had two owners, two surfaces which each had a fold contour projecting in a perspective to the same occluding contour. This would result in a border dispute! Fortunately, in general this does not happen. To show this we first define a surface created for the first owner. We show generically there would be no second owner.

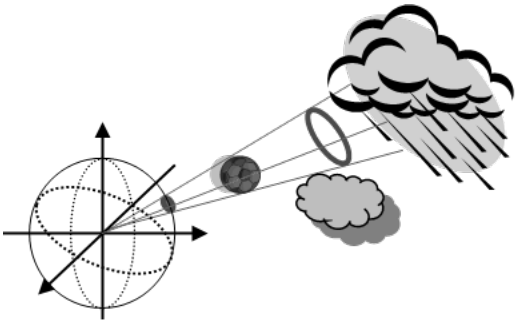

Figure SM13. A fold cone surface is the cone of lines passing fold points, which are also the rays on the occluding contour. The upper cloud surface is in a generic position with this cone, while the lower cloud surface is not.

Definition (**Fold Cone Surface**) Given an environment surface with fold in a perspective, the surface formed by lines passing the center of the perspective projection and points on the fold contour is called a *fold cone surface* for the environment surface.

The law governing interrelations between smooth surfaces in 3D Euclidean space and contours on a 2D manifold is *transversality*. In Guillemin and Pollack (15), it is defined:

Definition (**Transversality**) Let i be the inclusion map of a submanifold $X \subset Y$, and $Z \subset Y$ be another submanifold of Y. Map i is transversal to Z if and only if for every $x \in X \cap Z$

$$T_x(X) + T_x(Z) = T_x(Y),$$

where $T_x(X)$ is the tangent space of submanifold X at x, $T_x(Z)$ is the tangent space of submanifold Z at x, and $T_x(Y)$ is the tangent space of the manifold Y at x. Notice that this equation is symmetric in X and Z. When it holds, we shall say that the two submanifolds are *transversal.*

It can be proven that two surfaces in 3D space are generically transverse, i.e., they are not in general (generically) tangent to another surface if they are not so made, and similarly contours in 2D space are generically transverse, i.e., a contour in a 2D manifold is not in general (generically) tangent to a different contour if it is not so made. Surfaces and curves either keep away from each other or intersect with a non-zero angle.

As shown in **Fig. SM13**, a fold contour is always accompanied by a fold cone surface. Intersections of the fold cone surface with environment surfaces other than the one containing the fold contour are transversal.

Theorem (One Owner) An occluding contour in a perspective has one and only one owner.
Proof: From the one-side projection theorem, one of two sides of an occluding contour is its owner, and we assign the orientation of the occluding contour to make the owner at the left. Now we show that there is

a surface extension from the right side which has no singularity on the preimage of the occluding contour. In general we can assume the occluding contour contains no T-junction points. Otherwise, we can separate the occluding contour to several open segments without the T-junctions and prove the result for each segment. For an occluding contour containing no T-junctions, there always is a regular sandwich domain in the ray space. With the fold contour of the preimage surface of the left side regular domain, we can construct a *fold cone surface* S_c. Let S_r be a surface continuation of the preimage surface of the right side regular domain. Two surfaces S_r and S_c intersect transversally. Because all points on the surface S_c project to the occluding contour, the intersection points also project to the occluding contour. We claim these intersection points are *regular points* of S_r for the projection to the ray space. Denote the unit sphere around the perspective center O by S_o. The derivative of the projection map $\varphi : S_r \rightarrow S_o$ at an intersection point x is the natural linear map between the corresponding tangent planes: $d\varphi_x : T_xS_r \rightarrow T_{\varphi(x)}S_o$. This linear map is degenerate only when the normal vector to the plane $T_{\varphi(x)}S_o$, which is along a line of the fold cone passing intersection point x, is on the tangent plane T_xS_r. Due to the transversality condition of the two surfaces, this cannot happen: The tangent vector of the curve of intersection of S_r and S_c is located in both the tangent planes T_xS_c and T_xS_r. The line $\overline{Ox}$ is on the tangent plane T_xS_c. If it is also on the tangent plane T_xS_r, the two tangent planes are the same. $T_xS_r + T_xS_c = T_xS_r \neq T_xE^3$, the 3D tangent space of the 3D Euclidean space E^3 at point x. Therefore $d\varphi_x$ does not degenerate and the point x is not a fold point of the right side preimage surface. Therefore the right side of the contour is not the owner of the occluding contour. *QED*.

Theorem (Separated Surface Continuation) The preimage surfaces of the side domains of the sandwich domain of an occluding contour have separated continuations.

Proof: If an open interval of an occluding contour contains T-junctions, we can divide the interval into open intervals without T-junction. Assuming an occluding contour contains no T-junction point, it has a regular sandwich domain. Denote the left and right regular side domains as D_l and D_r and their preimages S_l and S_r. The natural perspective to a ray space is a one-to-one mapping from the visible part of environment to the ray space. Therefore, because D_l and D_r do not intersect, the two local surfaces S_l and S_r have no intersection. If $\overline{S_l} \cap \overline{S_r} = \emptyset$, the occluding contour will be a contour of surface separation: since their closures are compact, there is a minimum Euclidean space distance δ between $\overline{S_l}$ and $\overline{S_r}$. An open covering of both $\overline{S_l}$ and $\overline{S_r}$ by open sets of environment surfaces with diameter smaller than δ will give a surface continuation of both S_l and S_r with no intersections. If $\overline{S_l} \cap \overline{S_r} \neq \emptyset$, the projection image of an intersection point in $\overline{S_l} \cap \overline{S_r}$ must be on $\overline{D_l} \cap \overline{D_r}$, the occluding contour. From the one-owner theorem, each intersection point is a regular point of $\overline{S_r}$, but singular point of $\overline{S_l}$. A point of a surface cannot be both a critical point and a regular point. Therefore $\overline{S_l} \cap \overline{S_r} = \emptyset$. *QED*.

The preimage surface of the owner side has a continuation across the fold that becomes occluded behind the preimage surface of the owner side. The preimage surface of the other side has a continuation across the contour of its intersection with the fold cone surface and also becomes occluded by the preimage surface of the owner side.

2.4b. Ad hoc surface representation and the extraction of occluding contours: Border of Accretion Criteria for Occluding Contours Theorem

The above theorem means that through occluding contours, the vision system can see surfaces extended into the *occluded* area. The vision system can see an occluded surface that has no image on the retina! Gibson called this the most radical discovery of the ecological approach to vision. The perception of an

occluded surface near an occluding contour is not inferred (a guess with high probability) based on past experiences. It is directly picked up from the structure of ambient lights. The topological construction of the environment is directly coded in the structure of ambient lights.

Through extraction of occluding contours the vision system makes a topological division of the set of regular values in the ray space such that (1) the components delineated by occluding contours are homeomorphic to their preimage surfaces; (2) the assigned owner side specifies how the preimage surfaces are connected to the preimage of the occluding contour; and (3) the occluding contours further specify how visible surfaces are continuously extended to the occluded surfaces, and thereby connected to the global surfaces. The occluding contours therefore fully specify the topology of a visible surface, including its connection to a global surface which is never fully visible. It is quite clear that within the scope of binocular stereopisis, a main mechanism for surface representation, this computational goal is achieved by the da Vinci stereopisis, not Wheatstone stereopsis.

Definition (**Global Surface**) A connected component of the visual environment is called a *global surface*.

The initial surface representation takes the form of "image segmentation" by occluding contours. The segmentation turns a perspective (image) into a countable collection of components, a symbolic representation of the environment surfaces. Being tied to a particular point of observation, it is an ad hoc surface representation.

Definition (**Regular Component, ad hoc Surface Representation, Owner of an ad hoc Representation**) In a given perspective, a maximum connected open set of regular values with respect to the partial order of $\subseteq$ is called a *regular component*. A tuple $T = (C,B)$ of a regular component C and its surrounding occluding contours B is called an *ad hoc* representation of a global surface, or an *ad hoc surface representation*. The global surface containing the preimage of the regular component is called the *owner* of the *ad hoc* representation.

Definition (**Topological Segmentation of Perspective**) Given a perspective on a ray space S, let each $T_i = (C_i, B_i)$, $i \in I$, be an ad hoc surface representation of the perspective, I a set of indices, the sequence $\{(C_i, B_i) | i \in I\}$ such that $\bigcup_{i \in I} C_i \cup B_i = \mathrm{S}$ is called the *topological segmentation* of the perspective.

The topological segmentation of a perspective is a representation of components of visible surfaces. Each component of a visible surface can be further divided into areas, based upon color, texture, and other meaningful environmental invariants.

So far we have deduced properties of occluding contours based upon the definition that they are images of fold singularities. These properties explain why a vision system can "see" occluded parts of a surface--at least see their existence and their connection to the currently visible part. Now we deduce characteristic properties of the occluding contours based upon the perspective mapping structure. These properties explain how the vision system can *extract* the "remotely" determined occluding contours from directly accessible ambient lights.

Definition (Regular Point with respect to a Mapping Structure, *M*-regular) A ray in a ray space is called a *regular point with respect to a mapping structure* if it is in the source domain of a nontrivial transition mapping of this mapping structure. The regular points with respect to a mapping structure are called *M-regular points*. Since an *M*-regular point is also an *M*-regular value, and vice versa, we do not distinguish them.

Definition (*M*-Regular Component and *M*-border) A maximum element of the set of connected open sets of *M*-regular points in a ray space with the partial order of the subset relation "$\subseteq$" is called an *M-regular component*. The boundary of an *M*-regular component is called the *M-border* of the M-component.

We already deduced from Sard's Theorem that almost all points in a ray space are regular points with respect to the perspective mapping structure. This leads to an important property: the boundary of an *M*-regular set is also a border between two *M*-regular sets, i.e., it separates two *M*-regular sets.

Ray spaces are compact 2D manifolds with the metric topology of the unit sphere. When a sequence of inner points in the source domain approaches (Cauchy converges to) a boundary point, the sequence of their image points also approaches a boundary point of the target domain. Therefore a transition mapping always has a continuous extension to the boundary of its source domain.

Definition: (Boundary Image of Transition Mapping, Side Image of an *M*-Border) The image of the boundary of the source domain of a transition mapping under its continuous extension is called the *boundary image* of the transition mapping. If a side domain of an *M*-border is the source domain of some transition mapping, the boundary image of the border is called a *side image of the M-border* under the transition mapping.

Now we characterize the *M*-borders in terms of their side images by transition mappings from one ray space to another. *M*-regular components are 2D open sets. Their boundaries are contours.

Definition (**Border of Accretion of a Transition, Border of Infinitesimal Accretion**) Given a mapping structure and an *M*-border in a ray space surrounded by a sandwich domain, if a transition mapping in the mapping structure maps both side domains of the sandwich domain to another ray space, such that the two side images of the *M*-border are *M-regular and have no intersection point*, we call it a *border of accretion of the transition*. The transition is called an *accretion transition* for the border. If for any positive real number ε, there is an accretion transition for the border with distance less than ε, we call the border a *border of infinitesimal accretion*.

Definition (Border of Accretion of a Mapping Structure) Given a mapping structure in the visual space, if for every point p on a border B in a ray space there is an open neighborhood segment S_p of p, $S_p \subset B$ and $p \in S_p$, that is a border of accretion of some transition, the border B is called a *border of accretion of the mapping structure*. If at every point on a border, there is an open segment neighborhood of the point that is a border of infinitesimal accretion, the border is called a *border of infinitesimal accretion of the mapping structure*.

Definition (**Deletion**) A *P*-transition is said to have a *deletion* towards a border in the target ray space, if its inverse *P*-transition is an accretion transition for the border.

Definition (Accretion Bordered Mapping Structure) A mapping structure of the visual space in which every *M*-border is a border of *accretion* of the mapping structure is called an *accretion bordered mapping structure*. It is called an *infinitesimal accretion bordered mapping structure* if every *M*-border is an infinitesimal accretion border.

Theorem (Border of Accretion Criteria for Occluding Contours) A contour in a perspective is an occluding contour if and only if it is a border of infinitesimal accretion of the perspective mapping structure. The transition pole of each accretion transition for the occluding contour is at the right side of the occluding contour, namely the opposite side of the owner of the occluding contour.

Proof: We first show that an *occluding contour* is locally a *border of infinitesimal accretion*. We do this by first showing that any *point* on an occluding contour has two side images after some transition with arbitrarily small distance which are *M*-regular and different. Since the mappings to side images are continuous along a border of two regular domains, the point has a neighborhood segment on the occluding contour such that every point within the neighborhood also has two different *M*-regular side images in the target ray space. Thus the border is an infinitesimal accretion border.

Let *b* be a point (ray) on the occluding contour in the ray space centered at *O*, and take a small segment σ of the occluding contour containing *b*, and a regular sandwich domain of this contour segment. This always can be done by avoiding the *T*-junctions along the occluding contour. Move the observation point *O* to a nearby position *O'* of distance less than some positive number ε. The transition plane determined by points *b, O, O'* intersects with the environment surfaces with curves *AB* and *CD* (**Fig. SM14**) where *A* and *D* are on rays of the boundaries of two regular side domains. We assume *C is* a regular value of projection to the ray space with base point at *O*, although *C* is occluded by point *B* (this is true generically). *B* and *C* are on the same ray *b*. *B* is the fold point of the surface. The occluding contours are contours of surface separation, so that $B \neq C$.

The tangent line $\overline{BC}$ of curve *AB* at point *B* divides the transition plane into two half planes, right half plane $H_r(b)$ and left half plane $H_l(b)$. The owner side is in the left half plane $H_l(b)$. We now show that when the transition pole *e* is on the opposite side of the owner in the right half plane $H_r(b)$, a small transition to perspective center *O'* will map ray *b* of the ray space *O* to two side images, ray *O'B* and ray *O'C*, in the ray space based at *O'*. And both are *M*-regular rays. This will finish the proof that an occluding contour is an infinitesimal accretion border.

We note that near the fold point *B*, the intersection curve of the environment surface with the transition plane has positive curvature (Koenderink & van Doorn 1976 describes local geometry around fold points). Without loss of generality, we assume between points *A* and *P* as shown in **Fig. SM14**, the curve has positive curvature. Therefore, we can limit the transition vector to a neighborhood of *O*, such that the tangent lines from the new perspective center to the curve *AP* has tangent points located within the curve *AP*. This is possible because the location of the tangent point depends continuously on the position of *O'*, and *B* is inside the curve *AP*.

Both curve *AB* and curve *CD* are visible from *O*. Therefore there is no environment surface inside the triangle-like area *OAB* bounded by line *OA* and line *OB* with base curve *AB*, and the triangle-like area *OCD* bounded by line *OC* and *OD* with base curve *CD*. *O'* is inside the area. Point *B* is visible from *O'*. Also because *O'* is not on the line segment *OB*, *C* is visible.

B is not a fold point in projection to *O'*, for otherwise line *O'B* would be equal to line *OB*. Using tangent line t_A of the curve *AB* at *A* as the base line, because of the positive curvature of the curve *BB'*, line *O'B'* is tangent to the curve *AP*, its angle with t_A is larger than that of ray *b*. Because the curve has positive curvature, the tangent point *B'* must be further away from the point *A* along the curve *AP* and located in the occluded area of perspective *O*. *B* is visible from *O'* and regular. This means ray *O'B* is a regular value of perspective projection. Because C is a regular value of projection to O, by local stability, it is also a regular value of projection O'. Both *B* and *C* are regular visible points in the perspective to *O'*. Ray *O'C* is regular value of perspective projection. Both side images of *b* are now located in the *M*-regular domain in the perspective centered at *O'*. Thus we proved the occluding contour is an accretion border of the perspective mapping structure. The above proof holds for any such *O'* in the ε neighborhood of *O*, where choice of ε is made to guarantee the tangent lines from *O'* to the curve *AP* is inside of the curve *B P*. Therefore an occluding contour is locally a border of infinitesimal accretion.

Now we show a border of accretion in some *perspective transition* is an occluding contour. We first show every point in a border of accretion is a critical value of the perspective projection. Suppose the contrary is true, then there is a point on the border of accretion that is regular value of the perspective projection. The set of regular values are open. There must be a connected neighborhood of this regular value consisting of regular values. Any transition mapping that maps this neighborhood into another ray space is a homeomorphism, and the two side images of the border segment within this regular domain are the same. This shows a border containing a regular value cannot be an accretion border of a *P*-transition. The singularities of a projection from a surface to the ray space are either fold points or cusp points. Cusp points are at the ends of the fold contours. The points of an open segment of a contour of critical values must be the values of the folds. Therefore, the border of accretion is an *occluding contour*.

The transition pole of the start ray space in an accretion transition of the occluding contour is always located on the right side of the occluding contour, namely the opposite side of the owner of the occluding contour. Suppose in a transition, the transition pole of the start ray space is located on the side of the owner. Referring to **Fig. SM14**, *b* is a point on the occlusion contour in the ray space centered at *O*. Let $\overrightarrow{OO''}$ be a transition vector of a transition in which the transition pole of the start ray space is located on the owner side of *b*. In order for the transition pole, namely the ray from *O* passing *O''*, to be on the owner side, the left side of *b*, the point *O''* of the transition vector must be in the left half plane $H_l(b)$. We claim such an transition cannot be an accretion transition of an occlusion contour containing *b*. Suppose this is not true, and a transition with transition vector ending in the left half plane $H_l(b)$ is an accretion transition of the occluding contour. Then there must be a small neighborhood of *B* on the surface that is regular in the perspective projection to *O''*. Therefore we can pick a point *P* on the intersection curve of the surface and the transition plane from *O* to *O''* containing *b* such that *P* is occluded in the perspective to *O* but visible to *O'',* and all points on the curve *AP* on the surface are regular in the perspective at *O''*. *P* is beyond the curve *AB* because it is not visible from *O*. We show this is impossible.

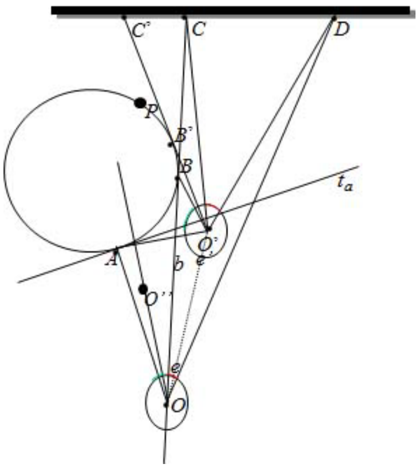

Figure SM14. Occluding contours are borders of accretion. *B* is a fold point for the perspective at *O*, but is regular for the perspective at *O'*.

All points on the curve *AP* on the surface are regular in the perspective at *O''*. Now the tangent line of curve *AP* passing *O''* has a smaller angle with t_a than that of *b,* and the curve *AB* has positive curvature, thus the tangent line from *O''* must be tangent to the curve *AB* at some point between *A* and *B*. Points *B* and *P* are behind this tangent point and are occluded in the perspective at *O''*. The contradiction shows *O''* cannot be the center of an accretion transition for *b*. Therefore for any accretion transition of an occluding contour,

the transition pole of the start ray space must be located on the side opposite the owner. *QED.*

2.4c. Specifying a persistent global surface: Global Surface from the Union Surface of a Complete Sample Theorem, Fixed Owner Theorem

Now we demonstrate the global surfaces of the visual environment can be specified by continuations of visible surfaces through accretion transitions. This means the distal global surfaces can be perceptually accessed through sequences of local surfaces coded in the form of perspective mapping triples.

Definition (Set of Regular Visible Surfaces): Let $\tau(P, Q)$ be the P-transition from a point P to another point Q, $\Sigma(\tau(P,Q))$ the set of *perspective surfaces* recovered from the correspondent domains of $\tau(P,Q)$. We call $\Sigma(\Omega) = \bigcup_{(P,Q)\in\Omega\times\Omega}\Sigma(\tau(P,Q))$ the set of *perspective surfaces* of the observation domain Ω. When Ω is the observation space, $P\in\Omega$, $\Sigma(P, \Omega) = \bigcup_{Q\in\Omega}\Sigma(\tau(P,Q))$ is called the set of *regular visible surfaces* at P. It consists of open sets of regular points on the environment surfaces visible from the perspective center P. In addition to these points, the fold points are also visible.

During locomotion or head/eye movement, the sampled local surface of a global surface is changing. Now we show that from the changing local surfaces, the vision system can see the invariant global surfaces of the environment.

Definition (the Complete Sample of a Surface) The equivalence class $C(\sigma_0) = \{\sigma \mid \sigma \sim_c \sigma_0 , \sigma\in\Sigma(\Omega)\}$ is called a *complete sample of surface* σ_0 in $\Sigma(\Omega)$, or simply a complete sample in $\Sigma(\Omega)$. Each element of a complete sample is called a *sample* surface.

Given a perspective transition $\tau(P,Q)$, the set $\Sigma(P,Q) = \Sigma(P)\cap\Sigma(Q)$ (= $\Sigma(\tau(P,Q))$) contains the surfaces visible from both P and Q. We call $D\Sigma(\tau(P,Q)) = \Sigma(P)\backslash\Sigma(\tau(P,Q))$ the set of *out-of-sight surfaces* of $\tau(P,Q)$, and $A\Sigma(\tau(P,Q)) = \Sigma(Q)\backslash\Sigma(\tau(P,Q))$ the set of *into-sight surfaces* of $\tau(P,Q)$. Obviously, from $\tau^{-1}(P,Q) = \tau(Q,P)$, and $\Sigma(\tau(P,Q)) = \Sigma(\tau(Q,P))$, we have $A\Sigma(\tau^{-1}(P,Q)) = D\Sigma(\tau(P,Q))$, and $D\Sigma(\tau^{-1}(P,Q)) = A\Sigma(\tau(P,Q))$.

Proposition Let σ_0 be a perspective surface, there is only one complete sample of the surface of σ_0 in $\Sigma(\Omega)$.

Proof: Suppose $C'(\sigma_0)$ is another complete sample of σ_0, any element $\sigma\in C'(\sigma_0)$ implies $\sigma \sim_c \sigma_0$, hence $\sigma\in C(\sigma_0)$. Therefore $C'(\sigma_0) \subseteq C(\sigma_0)$. Similarly, we have $C(\sigma_0) \subseteq C'(\sigma_0)$. Therefore $C'(\sigma_0) = C(\sigma_0)$. *QED.*

Thus the *complete sample of a surface* defines a partition of $\Sigma(\Omega)$, i.e., is an equivalence class.

Theorem (Global Surface from the Union Surface of a Complete Sample) Let Ω be the observation space. The surface of the union of samples in a complete sample in $\Sigma(\Omega)$ is a global surface.

Proof: A global surface is a compact connected component of a 2D manifold embedded in the 3D Euclidean space. The proof is in two steps. We first show that the maximum element is a global surface. Then we show that the union of complete samples is a maximum element. Therfore the union of complete samples must be a global surface. Given a surface σ_0, let σ_m be its maximum continuation, we claim σ_m is a global surface. Certainly σ_m is a an open, connected surface because it is a union of an open, connected cover of σ_0. It is also closed. For if it is not then its closure must be different from it, and further continuation can be made. This contradicts the fact that it is a maximum element with respect to surface continuation. The maximum element of such a set is unique. For if there are two such maximum elements, they have non-

empty intersection σ_0, so their union will be a continuation of σ_0 containing both as subsets. This contradicts the fact that they are maximum elements.

Now we show that the union surface from a complete sample is also a maximum element with respect to the partial order of surface continuation. The union surface from a complete sample must be an open subset of the maximum element. We show it is also closed. If it is not closed, its closure must contain a point that is on the maximum element but not in the union of the members of the complete sample. Let *p* be a point of the closure of a perspective syrface σ of the sample that is not in the union of members of the complete sample. We now prove such a point *p* cannot exist. Point *p* must be a boundary point of σ, for otherwise it is already a point of the union of the complete sample. If *p* is a regular point, its image has an *M*-regular domain *s*. The preimage of *s* is a neighborhood of p. The union of the preimage of *s* with σ is a new sample because σ and *s* have non-empty intersection. This contradicts the assumption that the complete surface includes all samples within the equivalence class. If *p* a singular point, its image under perspective projection must be on an occluding contour. An occluding contour is a border of infinitesimal accretion. The transition that makes the side images of the image of point *p* into *M*-regular domains will reduce this situation to the previous one: either side image of such a *P*-transition will turn the singular preimage point into an *M*-regular point. Therefore, such a point *p* cannot exist. This shows the union of the complete sample of a surface is a global surface. *QED*.

Definition (Perspective Inspection Path and Perspective Inspection Groupoid) A perspective inspection path is defined recursively. A perspective transition is a perspective inspection path with two ordered footprints: the start point and the end point. Two perspective inspection paths are consecutive if the end point of one is the start point of the other. In this case, we call the first one the prior and the second one the posterior. The composition of two consecutive perspective inspection paths is a path with footprints the concatenation of the footprints of the two consecutive perspective inspection paths such that the start point of the prior is the start point and the end point of the posterior is the end point. The groupoid formed from perspective inspection paths is called the perspective inspection groupoid, or *PI*-groupoid. An element of the *PI*-groupoid is called a *PI*-path.

Definition (**Persistent *ad hoc* representations through *PI*-paths, *PI*-path equivalent *ad hoc* Representations**) If the preimage surface of a regular component Δ of a perspective is not in the out-of-sight part of a *P*-transition, there is a correspondent visible component in the target perspective of the *P*-transition such that their pre-image surfaces overlap. The two pre-image surfaces belong to the same complete sample. We call the first pre-image surface persistent in the *P*-transition and the second pre-image surface a persistence of the first one. In this case, there is a mapping triple in the perspective mapping structure that maps a subset of Δ to a subset of a regular component Δ' in the second perspective. The *ad hoc* surface representation of Δ' and its borders in the second perspective is called a persistence of the *ad hoc* representation of Δ and its borders in the first perspective. Let $\{\tau(P_i, P_{i+1}) | P_1, P_{i+1} \in \Omega; i = 1, ..., n\}$ be a *PI*-path starting from position P_1 and ending at position P_n. Let $\sigma(\Delta_i) = (\Delta_i, B_i)$ be the *ad hoc* representation of a surface, where $\Delta_i \in S(P_i)$ a regular component, B_i its border, $i = 1, ..., n, n+1$. If $\sigma(\Delta_{i+1})$ is a persistence of $\sigma(\Delta_i)$, $i = 1, ..., n$, we call the *ad hoc* representations of surface $\sigma(\Delta_i)$, $i = 1, ..., n$, persistent through the *PI*-path. Persistence through *PI*-paths is an equivalence relation of *ad hoc* representations of a surface. Two *ad hoc* rperesentations are called *PI-path equivalent* if there is a *PI*-path such that one is a persistence of the other through the *PI*-path.

Theorem (**Fixed Owner**) The owner of the *ad hoc* representations persistent through a *PI*-path is fixed.

Proof: The preimage surface of the regular component of the persistence of an *ad hoc* representation has non-empty overlap with the preimage surface of the regular component of its prior *ad hoc* representation. They belong to one equivalence class with respect to the relation $\sim_c$. The global surface of the union of the

members of the complete sample is fixed. *QED*.

Persistence of the *ad hoc* representations of a surface through *P*-transitions maintains *invariance* of the owner under variations of the samples. Gibson made a careful distinction between the perception of *persistent surfaces* and the *persistent perception* of surfaces. Persistent perception of a surface implies some kind of memory mechanism to link different samples. Perception of a persistent surface is the extraction of the fixed owner from its persistent *ad hoc* representation through various *PI*-paths.

3. Extracting transition vectors from perspective transitions

In perspective transition, vision system does not see any change of the environment. Instead, it generates a sense of its own movement. *Visual kinesthesis* is "a fact of psychology" that is ignored in the egocentric depth map formulation of distance perception. There is a critical difference between perceiving an object moving toward you, and perceiving that you are moving toward an object. The latter is what is actually perceived during ego motion through an invariant 3D environment. Information about the movement of the eye in space underlies all significant visual processes for extraction of invariant surfaces. Perspectives are structured through *P*-transitions in a way isomorphic to eye movements. It is from the structured perspectives, the groupoid of perspective transitions, that the invariant environment surfaces can be extracted. Without the groupoid structure, a sequence of perspectives is only a pile of highly redundant 2D images, from which no invariant surface can be extracted. The transition vector is what makes two perspectives a *P*-transition.

How does the vision system extract information about the movement of the eye when there is a *P*-transition? Poincarè thought motion information is derived from the motor control signal accompanying the body, head, and eye movements. Gibson pointed out a visual channel of ego motion information must exist for the simple reason that a person sitting in a vehicle still has ego motion information.

The mathematical relation between transition vector and the perspective transition can be demonstrated with a pinhole camera model and an image plane coordinate system associated with the camera (**Fig. SM7**). In such a system, camera movement includes a translation from one point in space to another as well as rotation around the point of projection.

Suppose a surface point *P*: (*X, Y, Z*) is projected to two correspondent points *p*, *p'* on the image plane in two views and measurements are made: *p*: (*x, y*) and *p'*: (*x' y'*). Let $R(\alpha,\beta,\gamma)$ be the rotation operator in terms of the three angles of rotation (yaw, pitch, and roll) around the *z*-axis, *y*-axis and *x*-axis, and (t_x, t_y, t_z) the translation of the camera. The *coplanar constraint* for the correspondent points on the image plane in the two views can be written as:

$$F_f(\alpha,\beta,\gamma,t_x,t_y,t_z) = ((x,y,f)\times(t_x,t_y,t_z))\bullet((R(\alpha,\beta,\gamma)\circ(x',y',f)) = 0$$

where *f* is the focal length of the camera.

The operation of $(t_x,t_y,t_z)\times\vec{v}=\vec{w}$ provided by the vector (t_x,t_y,t_z) using the cross product is a linear transformation on the 3D vector space and can be expressed by a 3 by 3 matrix:

$$T(t_x,t_y,t_z) = \begin{bmatrix} 0 & -t_z & t_y \\ t_z & 0 & -t_x \\ -t_y & t_x & 0 \end{bmatrix}.$$

And the coplanarity condition can be expressed as:

$$F_f(\alpha,\beta,\gamma,t_x,t_y,t_z) = (T(t_x,t_y,t_z)\circ(x,y,f))\bullet(R(\theta,\varphi,\psi)\circ(x',y',f)) = 0\,.$$

The coplanarity constraint is a system of homogeneous equations and consequently its solution carries an undetermined *scale* in motion vector. This can also be seen from the "motion-and-structure from image motion" system of equations:

$$x_i' = \frac{(r_{11}(\rho_i x_i - t_x) + r_{12}(\rho_i y_i - t_y) + r_{13}(\rho_i f - t_z))}{(r_{31}(\rho_i x_i - t_x) + r_{32}(\rho_i y_i - t_y) + r_{33}(\rho_i f - t_z))}, \text{ and}$$

$$y_i' = \frac{(r_{21}(\rho_i x_i - t_x) + r_{22}(\rho_i y_i - t_y) + r_{23}(\rho_i f - t_z))}{(r_{31}(\rho_i x_i - t_x) + r_{32}(\rho_i y_i - t_y) + r_{33}(\rho_i f - t_z))},$$

where ρ_i is the distance of the *i*-th point P_i from the origin *O*, representing the 3D geometric structure of the rigid environment, and $r_{kl} = r_{kl}(\alpha,\beta,\gamma)$ is the *k*-th row *l*-th column element of the rotation matrix $R = R(\alpha,\beta,\gamma)$. The system of equations will not change if (t_x,t_y,t_z) is multiplied by a scale factor *s*, provided that all ρ_i in this system of equations are also multiplied by the same scale factor.

From a *P*-transition, there is an equivalence class of 3D representations of the transition vector and the vectors from the point of observation to the points of the environment surface points. The ratio between the scale of transition vector and the scale of distances between points in the rigid environment is invarfiant, not the *absolute scale* of the transition vector or the distance between points of the rigid environment, define a *rigid structure* of the environment. That is to say, the invariant "length" of the eye movement is the ratio of measurements of the magnitude of th transition vector and the distance of a fixed pair of ground points, or some rigid body of the envirornment, such as a standard "meter" or a ruler, or any two fixed points on the ground,etc.

By taking the transition vector as the unit for measuring distances between points on rigid bodies of the environment, the transition vector can be represented in a polar coordinate system with two angles: $(t_x,t_y,t_z) = (\sin(\theta)\cos(\phi), \sin(\theta)\sin(\phi), \cos(\theta))$, and the matrix

$$T(\theta,\phi) = \begin{bmatrix} 0 & \cos(\theta) & \sin(\theta)\sin(\phi) \\ -\cos(\theta) & 0 & -\sin(\theta)\cos(\phi) \\ -\sin(\theta)\sin(\phi) & \sin(\theta)\cos(\phi) & 0 \end{bmatrix}$$

has two variables. The coplanar constraint equation has five parameters to be determined:

$$F_f(\alpha,\beta,\gamma,\theta,\phi) = (T(\theta,\phi)\circ(x,y,f))\bullet(R(\theta,\varphi,\psi)\circ(x',y',f)) = 0\,.$$

There needs five correspondent points between two consecutive frames to fully constrain the *orientation* of the transition vector and the 3D rotation of the camera.

Although the *scale* of movement is not an *environment invariant*, the *direction* is. If *T* is part of the solution of the coplanarity equation, -*T* also is. However, only one is true. There needs a check on whether two *coplanar rays* intersect to determine the *direction* of movement.

In above formulation, the rigid motion of a pinhole camera is specified by five parameters: two parameters for the direction of camera translation, and three parameters for camera rotation around its focal point. The equation is non-linear. The computer and robotics vision studies of egomotion are mostly based upon two popular concepts: essential matrix (16) and fundamental matrix (17). In the essential matrix the five rigid motion parameters are mixed into eight substantial parameters. By imposing a minimization problem within a 5D manifold as a minimization within a 8D manifold, the essential matrix method effectively gave up the most essential geometric constraint of rigid motion, seems unlikely to be able to achieve the very goal of computing rigid motion.

From a *P*-transition, there is an equivalence class of 3D representations of the transition vector and the vectors from the point of observation to the points of the environment surface points. What is *invariant* is the ratio between the scale of transition vector and the scale of distances between points in the rigid environment, not the *absolute scale* of the transition vector or the distance between points of the rigid environment, define a *rigid structure* of the environment.

4. Extracting perspective mappings from perspective transitions

Having shown that in vision surface information is represented by *mapping triples* of the visual space (section 2.3), we now address the problem of how to pick up these *triples* from the structure of ambient lights, in particular the perspective transitions. A detailed description of these ideas is given in (18, 19).

The images are the samples taken from optical arrays. They form a subset of the Hilbert space of real-valued functions defined on 2D domains. Our goal is to pick up the information of *perspective mappings* in the form of the *transformations* between stereo pairs of images. By using the term "information" we mean (1) the *qualitative* information about *whether there* is a space-like coplanar transformation between image patches of different perspective; and (2) *numerical descriptions for* such transformations.

Our computational approach to *perspective transformation* is through *Taylor approximation*. The derivatives are a sufficient tool to judge if there is a diffeomorphism between small domains around a pair of correspondent points. Guillemin and Pollack made the following comment on the role of the determinant of the derivative of a mapping: "a truly remarkable and valuable fact. …… tells us that the seemingly quite subtle question of whether *f* maps a neighborhood of *x* diffeomorphically onto a neighborhood of *y* reduces to a trivial matter of checking if a single number – the determinant of df_x – is nonzero!"

A perspective mapping is a mapping between two ray spaces by way of coincidences on environment surfaces. An exact numeric description of a diffeomorphism by a Taylor expansion requires infinitely many real numbers. However, we can make the following compromises: (1) working on only small domains of neighborhoods of a set of grid points, and (2) tolerating certain levels of error. What we gain from this compromise are: (1) by using the first order Taylor expansion we need *only three numbers* to provide a local description of the perspective mapping; (2) the approximation provided by the three numbers covers a truly 2D domain nearby the grid point. Therefore although the number of the grid points may be large, it is a *finite set* in each bounded 2D area. In practical situations, the approximations based upon these grid points may be sufficiently accurate.

An image can be regarded as a linear summation of Hilbert space components. Even from a small image patch, we can pick up plenty of different Hilbert space components. By selecting a set of *linear independent*

basis elements, we can obtain a set of *coefficients* of these basis elements through linear space projections. The perspective transformations act on images as linear operators on a Hilbert space, acting on each component of an image in the same way as the image. The role that perspective transformations play to change images into different images is different but related to the original role of perspective transformations for mapping an open set to another open set as originally defined. The transformations as linear operators on the Hilbert space are *representations* of the original perspective transformations. In vision, we extract the perspective transformations from their representations as linear operators on images (for discussion of the representation of transformation groups, see (20)).

Suppose $A(S(O))$ is an ambient optic array defined on the ray space S(O), and $U \subset S(O)$ is a domain in the ray space. The part of the optic array on U is denoted by $A_O(U)$. Similarly, we have $A_{O'}(U')$, $U' \subset S(O')$. The action of the transformation g can be expressed by the equation

$$A_O(U) = A_{O'}(g(U)). \tag{1}$$

Sampled into images from two locations O and O', image matching between "image patches" $f_O(U)$ and $f_{O'}(U')$ *gives:*

$$f_O(U) = f_{O'}(g(U)). \tag{2}$$

This gives the transformation g the proper representation in the Hilbert space of images.

The neurons in the primary visual cortex of monkeys have receptive fields. The response signal to an image patch $f(U)$ can be treated as the inner product of the receptive field functions (*rf*-functions) $F_i(U)$, $i = 1, 2, ..., n$ of the neuron on the patch with $f(U)$:

$$\gamma^i(U) = < F_i(U), f(U) >, \ i = 1, \dots, n.$$

Equation (2) implies

$$< F_i(U), f_o(U) > = < F_i(U), f_{o'}(g(U)) >, \ i = 1, \dots, n. \tag{3}$$

The system of equations (3) is a "weak" form of relation (2) as it checks only n "response" signals, while the single equation (2), the *true equation* for images, represents infinitely many of these component equations. Our original formulation of picking up transformation from *image pairs* now turns into the formulation of picking up transformation from some *Hilbert space components* of the sampled image pairs. The change raises two questions: (1) Will this equation be mathematically solvable; and (2) will the solution of this "weak" form equation still yield the transformation we are looking for?

With reference to **Figs. SM7 and SM8**, when the image plane makes a parallel shift along the y-axis, at each point the first order Taylor expansion of the coplanar diffeomorphism g is an affine transformation with three parameters. At point p_0, we use $A\left(\left.\frac{\partial y'}{\partial x}\right|_{p_0}, \left.\frac{\partial y'}{\partial y}\right|_{p_0}, \delta_y(p_0)\right)$ as an approximate representation of g and measure the *degree of matching* within the scope U. Using $a_{21} = \left.\frac{\partial y'}{\partial x}\right|_{p_0}$, $a_{22} = \left.\frac{\partial y'}{\partial y}\right|_{p_0}$ to simplify notation, and letting $t = \delta_y(p_0)$ represent the "disparity" of center p_0 when O moves to O', we have the following system of equations:

$$< F_i(U), f_o(U) >=< F_i(U), f_{o'}(A(a_{21}, a_{22}, t) \circ U) >, \ i = 1, \ldots, n.$$

The equations for the three variables a_{21}, a_{22}, and t form a nonlinear system of equations. We can use a gradient method to solve for the three unknowns. However, to compute the gradients requires taking *Lie derivatives* on the components against the parameters of transformation. Because the images are assemblages of measurements of the intensity of incident lights impinging on the sensor units, they have no analytical form that we can compute derivatives from.

From the Hilbert space representation concept, we can view the affine transformation on U as a linear transformation on the function, denoted by the same symbol, so that we have:

$$< F_i(U), f_o(U) >=< F_i(U), A(a_{21}, a_{22}, t) \circ f_{o'}(U) >, \ i = 1, \ldots, n.$$

Let $A^*(a_{21}, a_{22}, t)$ be the conjugate transformation of $A(a_{21}, a_{22}, t)$ with respect to the inner product. Finally we have derived the system of equations that we can solve:

$$< F_i(U), f_o(U) >=< A^*(a_{21}, a_{22}, t) \circ F_i(U), f_{o'}(U) >, \ i = 1, \ldots, n. \qquad (4)$$

The process for picking up the transformation can now be formulated as minimizing the differences between the components of the image and its transformation. The objective function for minimization, the energy E with three undetermined parameters t, a_{21}, and a_{22}, is the following:

$$E(a_{21}, a_{22}; t, U, o, o') = \sum_{i=1}^{n} \left\| < F_i(U), f_o(U) > - < A^*(a_{21}, a_{22}, t) \circ F_i(U), f_{o'}(U) > \right\|^2 .$$

Equation (4) has important operational consequences. We are seeking a mechanism for the visual system to pick up the 2D to 2D transformations, the *numeric description* of the perspective mapping. The input from ambient optic arrays is in the form of a pair of similar images. The transformation itself is implicit in the data. It is up to the vision system, with its processing mechanisms, to pick this up. In terms of inner products and conjugate transformations, and with a set of *analytical rf*-functions, a gradient dynamical system (21) for determining $dg(p_0)$ in terms of a_{21} and a_{22}, and the shift t is fully specified, in which the Lie differentiations against the parameters t, a_{21}, and a_{22} are represented by a type of *linear operator* derived from *rf-functions* known as *Lie germs*.

In Equation (3), the perspective transformation represents a physical fact resulting from different perspective projections. In Equation (4), however, the transformation becomes the “duty” of receptive fields of neurons in the vision system to make the Hilbert space components match! Nature has made a mapping resulting in a changed image when the eye moves from one point of observation to another, it is up to the brain to respond to this mapping with its own transformation. Equation (4) means the *responsibility is* given to the receptive fields of neurons. By so responding, the perspective mapping is picked up by the neurons, in the form of the *conjugate* transformation. It is conceivable that since nature supplies transformations that can be approximated with only three real parameters, the dynamical receptive fields of the neuron may also controlled by three real parameters.

Does the weak form of equation (1) discard useful information and lead to a distorted version of the true perspective mapping? Lights emitted or reflected from the same source or surface point may not have the

exact same optical characteristics when seen at different points of observation. Furthermore, they must pass the medium and lens to be sensed. Thus noise, disturbances, and geometric distortion are inevitable. The visual system model, like any meaningful physical system model, must assume certain stability criteria: percepts (system prediction) should be stable under perturbations on measurements (22).

In equation (3), many aspects of the ambient lights $f_o(U)$ and $f_{o'}(g(U))$ must be "ignored" and others emphasized by properly choosing $F_i(U)$ ($i= 1, 2, ..., n$), in terms of frequency, scale, and orientation, such as band pass 2D Gabor functions, so that both *high frequency random noise* and DC components (constant intensity shifts) will not affect the measurement results. Instead, the measurements will specifically respond to the stable characteristics of the ambient light, such as orientation specific contrasts associated with textures of the surfaces.

Picking up *perspective transformation* is equivalent to picking up the *surface patches*. Detecting *the accretion border of the perspective transformation* is equivalent to picking up the *occluding contours*. The computation of perspective transformation is the very *core* of *surface perception*. A detailed description of the algorithm can also be found in (19).

5. Summary

Unlike taste and touch, the function of vision is to allow an animal to know the environment without immediate body contact. Psychologists call objects distant from the body of an animal the "distal stimulus," and those immediately contacting the sensory system the "proximal stimulus." In vision, the link between the distal and proximal stimulus is given by lights reflected from environment surfaces. Gibson called the laws governing the light rays reflected from environment surfaces the "ecological optics."

In this article, we first introduced two topological spaces: one for describing the 3D objects in the environment, and one for describing the light rays reflected from the environment objects. The first space is the familiar 3D Euclidean space. The second space is the set of ray spaces assigned to each observation point in the medium, and the ambient optic array is the particular pattern of light rays residing in this space.

The concept of ambient optic array is the key to understanding how visual surface perception works. Eyes are often modeled as pinhole cameras. The ambient optic array converging to the potentional pinholes constitutes the physical reality within which the vision system evolved.

With the proper spatial concepts, we show how the 3D objects and their surfaces, contours, domain-connected components, etc., are able to impose structures of rays in the visual space through perspective projections and create the two most important structures in ecological optics, the *perspective mapping structure*, and the *occluding contours* at each perspective. The first has the form of a *pseudogroup*, and the second is a very peculiar mathematical structure, the critical values of perspective projections in each ray space. With these concepts we developed a new type of local surface representation, *ad hoc surface representation*, which encodes all the contiguous surfaces and their overlap relationships at a single perspective, and a new type of global surface representation, *persistent surface representation*, which encodes the globally invariant surfaces in the environment through overlaps between different ad hoc surface surface representations.

A major puzzle in vision research is how the objects and their surfaces are perceived as invariant in outer space, from changing perspective images (e.g., see (23)). We reformulate this problem as one of *how an equivalence class* of topological objects in the visual space represents a unique object in the 3D Euclidean space. The persistent surface representation is specified by the equivalence relation of *chain connectedness*.

The mathematical theory presented in Sections 2 and 3 concerns only the air and the surfaces in the

environment. It has nothing to do with the vision system. It describes the topologic and geometric structure present in the animal's optical environment. Because we are dealing with optics, everything can be addressed in a pure and exact sense. Now, if the light carries the information about the topologic and geometric structure, and the eye is able to see this information in the lights, this makes vision possible. Here approximation and ambiguity may occur. In Section 4, we describe one possible algorithm by which the visual system may extract perspective mappings computationally in order to perceive invariant surfaces.

The concept of topological information processing as a major component of vision has far reaching consequences for our understanding of how biological vision functions and how computational vision algorithms should be designed.

References

1. Helmholtz H (1925) *Physiological Optics* (Optical Society of America).
2. Gibson JJ (1979) *The Ecological Approach to Visual Perception* (Houghton Mifflin, Boston).
3. Poincarè H (1905) *Science and Hypothesis* (Dover Publications, New York).
4. Dunford E & Schwartz JT (1964) *Linear Operators Part I: General Theory* (Interscience Publishers, New York).
5. Brown R (1968) *Elements of Modern Topology* (McGraw Hill, Maidenhead).
6. Brandt W (1926) Über eine Verallgemeinerung des Gruppengriffes. *Math. Ann.* 96:360-366.
7. Higgins PJ (1971) *Categories and Groupoids* (Van Nostrand Reinhold Company, London).
8. Warner FW (1983) *Foundations of Differentiable Manifold and Lie Groups* (Springer-Verlag, New York).
9. Golubitsky M & Guillemin V (1973) *Stable Mappings and Their Singularities* (Springer-Verlag, New York-Berlin-Heidelberg-Tokyo).
10. Kobayashi S & Nomizu K (1963) *Foundations of Differential Geometry* (John Wiley & Sons, New York).
11. Klingenberg W (1977) *A Course in Differential Geometry*.
12. Whitney H (1955) On Singularities of Mappings of Euclidean Spaces. I. Mappings of the Plane into Plane. *The Annals of Mathematics* 62(3):374-410.
13. Arnold VI (1981) *Singularity Theory* (Cambridge University Press Cambridge, MA).
14. Marr D (1982) *Vision* (Freeman, New York).
15. Guillemin V & Pollack A (1974) *Differential Topology* (Prentice-Hall, Englewood Cliffs, New Jersey).
16. Longuet-Higgins H (1981) A computer algorithm for reconstructing a scene from two projections. *Nature* 293:133–135.
17. Luong QT & Faugeras OD (1996) The fundamental matrix: Theory, algorithms and stability analysis. *The International Journal of Computer Vision* 1(17):43–76.
18. Tsao T & Tsao D (1997) Lie group model neuromorphic geometric engine for real-time terrain reconstruction from stereoscopic aerial photos. *Applications and Science of Artificial Neural Networks III,* Proceedings of SPIE, ed Rogers SK), Vol 3077, pp 535-544.
19. Tsao T & Kanal L (1999) A Scene Registration Method based on a Dynamical Receptive Field Model of Biological Vision. *Pattern Recognition Letters* 20:1423-1430.
20. Naimark MA & Štern AI (1982) Theory of Group Representation. (Springer-Verlag, New York, Heidelberg, Berlin), p 158.
21. M.W. H & Smale S (1974) *Differential Equations, Dynamical Systems, and Linear Algebra* (Academic Press, San Diego, New York).
22. Abraham R (1967) *Foundations of Mechanics* (W.A. Benjamin, New York).
23. McCulloch W & Pitts W (1947) On how we know universals: The perception of auditory and visual forms. *Bulletin of Mathematical Biophysics*:127-147.
